# Changes in soil organic carbon in croplands subjected to fertilizer management: a global meta-analysis

**DOI:** 10.1038/srep27199

**Published:** 2016-06-02

**Authors:** Pengfei Han, Wen Zhang, Guocheng Wang, Wenjuan Sun, Yao Huang

**Affiliations:** 1 LAPC, Institute of Atmospheric Physics, Chinese Academy of Sciences, Beijing 100029, China; 2 University of Chinese Academy of Sciences, Beijing 100049, China; 3 State Key Laboratory of Vegetation and Environmental Change, Institute of Botany, Chinese Academy of Sciences, Beijing 100093, China

## Abstract

Cropland soil organic carbon (SOC) is undergoing substantial alterations due to both environmental and anthropogenic changes. Although numerous case studies have been conducted, there remains a lack of quantification of the consequences of such environmental and anthropogenic changes on the SOC sequestration across global agricultural systems. Here, we conducted a global meta-analysis of SOC changes under different fertilizer managements, namely unbalanced application of chemical fertilizers (UCF), balanced application of chemical fertilizers (CF), chemical fertilizers with straw application (CFS), and chemical fertilizers with manure application (CFM). We show that topsoil organic carbon (C) increased by 0.9 (0.7–1.0, 95% confidence interval (CI)) g kg^−1^ (10.0%, relative change, hereafter the same), 1.7 (1.2–2.3) g kg^−1^ (15.4%), 2.0 (1.9–2.2) g kg^−1^ (19.5%) and 3.5 (3.2–3.8) g kg^−1^ (36.2%) under UCF, CF, CFS and CFM, respectively. The C sequestration durations were estimated as 28–73 years under CFS and 26–117 years under CFM but with high variability across climatic regions. At least 2.0 Mg ha^−1^ yr^−1^ C input is needed to maintain the SOC in ~85% cases. We highlight a great C sequestration potential of applying CF, and adopting CFS and CFM is highly important for either improving or maintaining current SOC stocks across all agro–ecosystems.

Cropland soil organic carbon (SOC) sequestration is crucial for both food security and climate change mitigation. It has been reported that increasing soil carbon (C) pool can help to enhance not only crop productivity but also yield stability^1^,^2^. Furthermore, soil is the largest reservoir of C in the terrestrial ecosystems, and a slight variation in this pool can result in substantial changes in the atmospheric CO_2_ concentration, thus potentially affecting global climate change^3^.

Globally, agricultural soil covers a total area of ~1370 million hectares (Mha) distributed across diverse climatic and edaphic conditions, as well as complex cropping systems and management practices^4^,^5^,^6^. Thus, the C input and SOC turnover are highly variable across spatial-temporal distributions, making the assessment of cropland SOC change more complicated^7^. Although the recommended management practices (RMPs) have been widely suggested to benefit the soil health^8^, the direction and the magnitude of such RMPs to affect SOC dynamics have exhibited large disparities across time and space. For example, field experiments on a one-hundred-year scale reported that the adoption of farmyard manure application increased the SOC by 200% at the Rothamsted site in the UK^9^ but decreased the SOC by 26% at Sanborn Field in the USA^10^. In another example, it has been documented that the adoption of straw retention continuously increased the SOC in Ultuna (Sweden) after 54 years^11^, while the SOC at Fukuoka (Japan) increased in the first 28 years but decreased during the later periods^12^. These spatiotemporal variations in the ability of RMPs to promote SOC sequestration can be attributed to the complex interactions among climate, soil and agronomic management practices that regulate SOC dynamics^13^. However, accounting for such effects of heterogeneity in climate and soil conditions and management practices over time and space on SOC at a global scale has rarely been reported so far.

The adoption of fertilizer management practices, e.g., chemical fertilization, manure application and straw retention, has been recognized to be the most efficient and effective manner to either promote SOC accumulation or reduce the rate of SOC loss. In areas with nutrient deficiency, chemical fertilizers increase the crop yield and biomass and thus the crop residue and root C input to soil^11^. Manure application and stubble retention are among the most predominant management practices driving SOC changes because they directly add C into soil^14^,^15^. Long-term field experiments have demonstrated that these RMPs significantly enhanced the SOC content^16^,^17^,^18^,^19^,^20^. Furthermore, with the expansion of conservation tillage^21^ and organic farming^22^, the residue incorporation and manure application rates are certain to increase in the areas where such RMPs are adopted, consequently promoting soil C sequestration^23^,^24^.

On one hand, the potential C sink of the world’s agricultural and degraded soils is tremendous, approximately 50–66% of the historic C loss of 42–78 Pg C (1 Pg = 10^15^ g)^1^; on the other hand, unbalanced chemical fertilizer applications and the absence of organic matter additions remain common worldwide according to the FAO statistical data (http://faostat.fao.org/site/575/default.aspx#ancor, accessed 12 January 2015), especially in developing countries^1^,^25^,^26^. Using Africa as an example, the average nitrogen (N) application rate from 2002–2012 was only ~14 kg ha^−1^ yr^−1^, which severely hampers the crop production. In addition, complete crop residue removal for fodder and fuel is a norm in South Asia and Africa^1^, making soils in these areas lack of organic matter input and liable to be C sources. Thus, there is a pressing need for a comprehensive evaluation of all fertilizer managements on SOC sequestration.

Previous studies mostly concentrated on a specific management practice on SOC change^14^,^15^,^27^ or on regional scales^28^,^29^,^30^,^31^,^32^,^33^, which can hardly present a complete perspective on the effects of various fertilizer managements on SOC dynamics across various climatic regions, edaphic conditions and complex cropping systems. Here, we conducted a global meta-analysis of published data on the responses of SOC to fertilizer managements in 1741 paired field experiments. Our objectives were to 1) quantify the SOC changes under different fertilizer managements and 2) identify and quantify the factors (i.e., environmental and management conditions) with the most influence on SOC changes.

## Results

### Natural logarithm of the response ratios (lnRR) of SOC to fertilizer management

**Figure 1:**
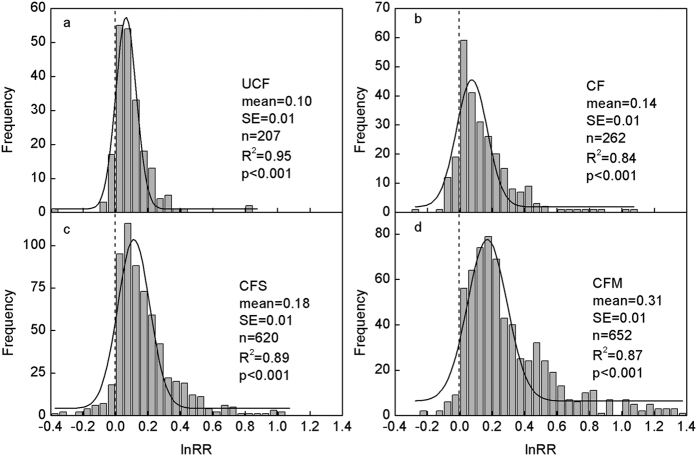
Frequency distributions of response ratios (lnRRs) for SOC under four treatments. UCF, CF, CFS and CFM represent the unbalanced application of chemical fertilizers, balanced application of chemical fertilizers, straw return and application of chemical fertilizers, and application of manure and chemical fertilizers. The solid curve is a Gaussian distribution fitted to frequency data. The vertical dashed line is at lnRR = 0.

All four of the fertilizer management practices increased SOC concentrations (Fig. 1). Straw retention and manure application induced greater increases in carbon (Fig. 1c,d) than that under the chemical fertilizer application (Fig. 1a,d). The mean lnRR of SOC was 0.10, 0.14, 0.18 and 0.31 under unbalanced application of chemical fertilizers (UCF), balanced application of chemical fertilizers (CF), chemical fertilizers with straw application (CFS), and chemical fertilizers with manure application (CFM), respectively. The lnRR differed largely among different treatments and sites, ranging from −0.4–1.4. The frequency distributions of lnRR under the four treatments could each be fitted by a Gaussian normal distribution, with R^2^ ranging from 0.84–0.95 and p < 0.001.

### Fertilization effects on C sequestration

**Figure 2:**
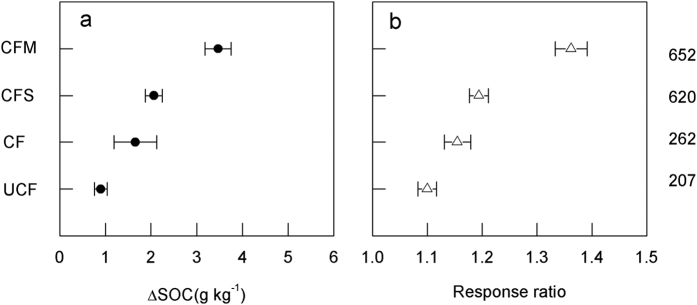
Mean difference in SOC (g kg^−1^) (**a**) and the relative change (**b**) compared with CK. Dots and bars represent the mean and range at 95% confidence intervals. The sample size for each group is shown on the right side of the figure.

Compared with the control (CK), all four of the fertilizer management practices significantly increased the SOC content at the end of the experiments (Fig. 2). The bootstrap 95% confidence intervals (CI) of the mean difference in SOC were greater than 0 and those of the response ratio (RR) were greater than 1, demonstrating a significant positive effect on C sequestration in the cropland topsoil (i.e., 0–20 cm) following fertilization and straw retention. The largest SOC increase occurred in the CFM treatment, with an average mean difference between SOC under treatment and CK (ΔSOC) of 3.5 (3.2–3.8, the lower and upper limit of the 95% CI, hereafter the same) g kg^−1^, followed by CFS (2.0, 1.9–2.2 g kg^−1^), CF (1.7, 1.2–2.3 g kg^−1^), and UCF (0.9, 0.7–1.0) g kg^−1^ (Fig. 2a). The average ΔSOC differed significantly among the four treatments (between-class homogeneity (Q_B_) = 1423.5, p < 0.001, Table S3). Regarding the relative change, the RR was 1.10, 1.15, 1.19 and 1.36 under UCF, CF, CFS and CFM, respectively (Fig. 2b).

### Effects of climate, experiment duration and C input on SOC changes

**Figure 3:**
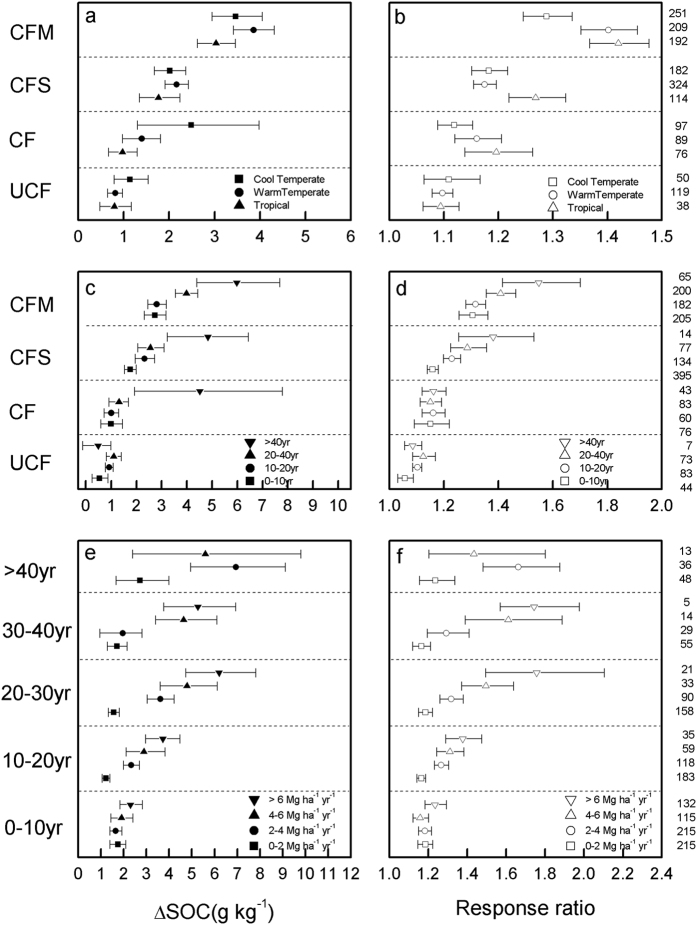
Effects of climate zone, experiment duration and C input on mean difference in SOC (g kg^−1^) (**a,c,e**) and the relative change (**b,d,f**) comparing with CK.

Under a given fertilizer management strategy, the average ΔSOC generally increased from tropical to cool temperate regions (Fig. 3a). The difference in ΔSOC was significant only under CF treatment across climate regions (Q_B_ = 5.47, p < 0.05), and the mean ΔSOC was 1.0, 1.4 and 2.5 g kg^−1^ in tropical, warm temperate and cool temperate regions, respectively. The CFS treatment followed the same trend as the CF. For the UCF group, the average ΔSOC was low in tropical and warm temperate regions (0.8 g kg^−1^) and high in cool temperate regions (1.1 g kg^−1^). For the CFM group, the average ΔSOC values were 3.0, 3.9 and 3.5 for tropical, warm temperate and cool temperate regions (Fig. 3a), respectively. Unlike the ΔSOC trends, the response ratios were generally low in the cool temperate and high in the tropical regions (Fig. 3b) due to initial SOC differences.

The mean values for ΔSOC and RR increased with experiment duration under most treatments (Fig. 3c). Our results showed that the CF, CFS and CFM treatments could sequester C for at least 40 yr, with the ΔSOC being 4.5, 4.8 and 6.0 g kg^−1^ in the respective subgroups for >40 yr (Fig. 3c). However, in the UCF group, the ΔSOC remained approximately 1.0 g kg^−1^ after 20 yr, indicating that the SOC tended to level off over time. Consistent with the ΔSOC trend, the response ratios were largest for the >40 yr subgroup and lowest for the 0–10 yr subgroup. At the end of the experiments, the SOC under UCF, CF, CFS and CFM increased to 1.09, 1.16, 1.38 and 1.55 times that under CK, respectively (Fig. 3d).

Increasing C input is one of the most efficient methods for soil C sequestration. As illustrated in Fig. 3e,f, the ΔSOC and RR increased with increasing C input levels in the same fertilization duration group. The average ΔSOC increased from 1.7–2.3 g kg^−1^ (1.18–1.24 times that in CK, hereafter the same), 1.2–3.7 g kg^−1^ (1.16–1.38 times), 1.6–6.2 g kg^−1^ (1.19–1.76 times), 1.7–5.3 g kg^−1^ (1.16–1.74 times) as C input increased from 0–2 Mg to over 6 Mg ha^−1^ yr^−1^ in the 0–10 yr, 10–20 yr, 20–30 yr and 30–40 yr groups, respectively. However, this trend could not be detected in the >40 yr group due to the limited sample size (n = 13) for the 4–6 Mg ha^−1^ yr^−1^ subgroup. In addition, the sequestration efficiency of high C input (e.g., >6 Mg ha^−1^ yr^−1^) gradually declined with experiment duration (Fig. 3e).

### SOC change rates and C sequestration duration

**Figure 4:**
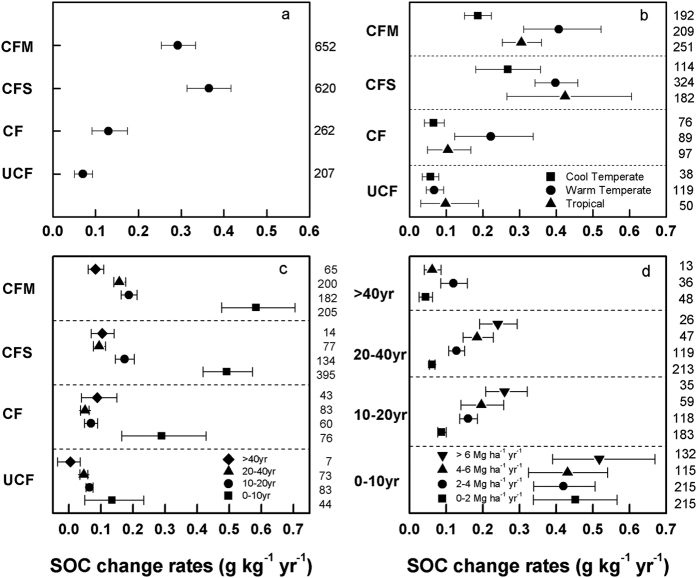
Rates of SOC change with different impact factors. The letters (**a–d**) denote fertilization group, climate zone, experiment duration and C input, respectively.

The relative rates of SOC change are significantly influenced by fertilization type, climate zone, fertilization duration and C input. The rate of SOC change was significantly higher under CFM and CFS (0.29 and 0.37 g kg^−1^ yr^−1^) than under CF and UCF (0.13 and 0.07 g kg^−1^ yr^−1^) (Q_B_ = 19.12, p < 0.001, Fig. 4a). The cropland soil sequestered C faster in tropical and warm temperate regions than in cool temperate regions under the same treatment (Fig. 4b). Moreover, the rates of SOC change vary significantly across climate zones under CF and CFM (Q_B_ = 1.20 and 5.60, p = 0.005 and 0.000, respectively). The rates of SOC change decreased significantly with increasing fertilization duration (Fig. 4c). Specifically, the greatest SOC change rates occurred during the first 10 yr for all treatments, ranging from 0.13 g kg^−1^ yr^−1^ under UCF to 0.58 g kg^−1^ yr^−1^ under CFM, and gradually diminished to less than 0.10 g kg^−1^ yr^−1^ after 40 yr. Generally, the rates increased synchronously with increasing amounts of C inputs in the same duration group (Fig. 4d).

**Figure 5:**
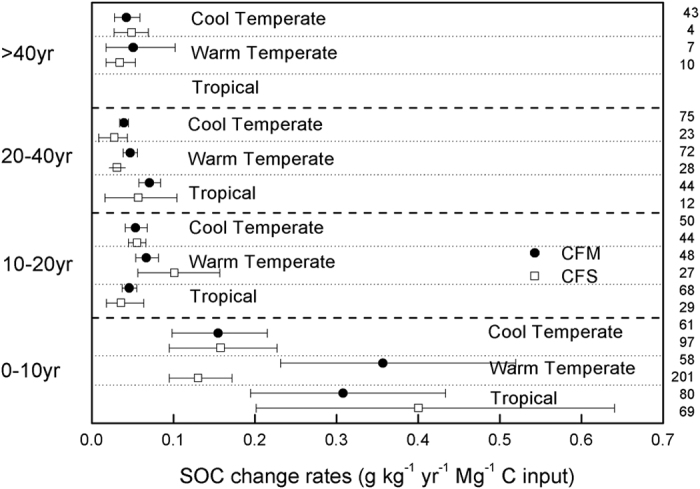
Comparisons of rates of SOC change per Mg C input of manure and straw.

In terms of the C sequestration efficiency, manure is usually more efficient than straw in most regions, especially in warm temperate regions during the first 10 yr, with C sequestration rates of 0.36 under manure application and 0.13 g kg^−1^ yr^−1^ under straw retention per Mg C input (Fig. 5). Notably, these differences slowly diminished after 40 yr.

**Figure 6:**
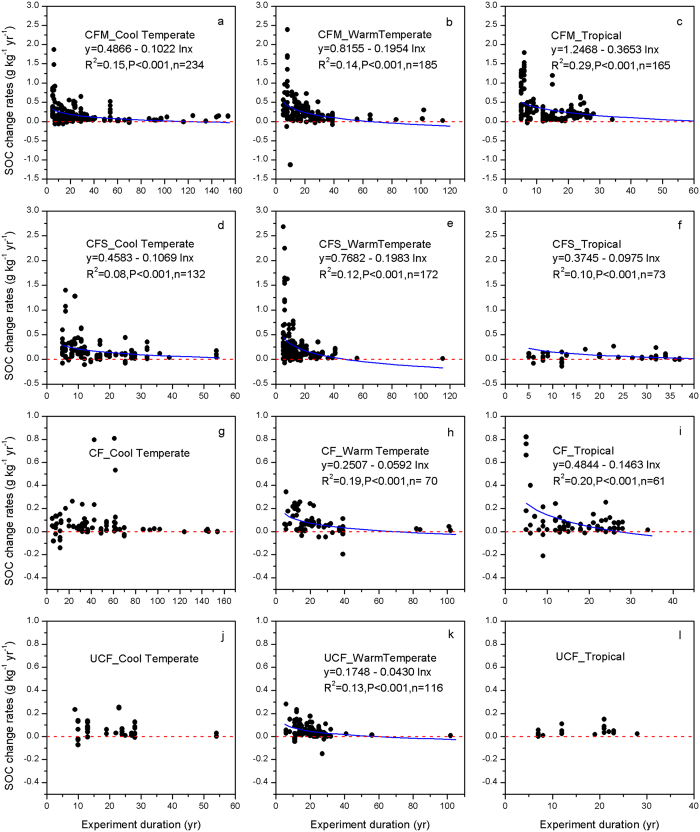
Relationships between the relative rates of SOC change and the duration (in years) in different climate regions. Experiments lasting more than 5 years were used to reduce the instabilities at the beginning of the experiment.

The rates of SOC change decreased with experiment duration, and thus the rates would reach zero or a lower limit that could be considered an equilibrium state. A logarithmic function was used to fit the rates of SOC change and the experiment duration. The C sequestration duration was calculated by setting the rates of SOC change to 0 and 0.05 g kg^−1^ yr^−1^ for the nine regression formulas in Fig. 6. Generally, the time needed to reach equilibrium was longer in cool temperate than in tropical regions. The duration of C sequestration under CFM was 72–117 yr in cool temperate, 50–65 yr in warm temperate and 26–30 yr in tropical regions (Table 1, Table S2). Following the same trend, the duration of C sequestration under CFS was 46–73 yr in cool temperate, 37–48 yr in warm temperate and 28–47 yr in tropical regions. As for the chemical fertilizer applications, C sequestration duration was estimated as 30–69 yr in warm temperate and 19–27 yr in tropical regions under CF treatment and 18–58 yr in warm temperate regions under UCF treatment (Table 1, Table S2). Nevertheless, for some climate regions, no apparent relationships were observed between rates of SOC change under CF or UCF treatment and experiment duration due to the limited sample size and large variability in sequestration rates.

## Discussion

It is well known that the loss of information caused by the exclusion of multiple results may become a more serious problem than that caused by violating the assumption of independence^31^,^34^. Thus, we also conducted a meta-analysis using multiple-year observations; these results are presented in supplementary information part 2. The magnitudes and directions of trends were generally consistent with the results using the last year observations across all groups (Figs 2, 3, 4, 5 and Figs S2–S10), but the significance levels may have been affected by the different sample sizes, which was consistent with previous studies^31^,^34^. Compared with the mean ΔSOC using the last year of observations (3.5 g kg^−1^, Fig. 2a), the result obtained using multiple-year observations was 4.2 g kg^−1^ under CFM (Fig. S3a), while Maillard and Angers^15^ estimated the ΔSOC was 1.0–4.1 g kg^−1^ under CFM. In contrast to the statistical insignificance in Fig. 3a for most groups (p = 0.08–0.29, Table S3), the differences of ΔSOC were significant under all four treatments across climate zones (p < 0.05, Fig. S4a). The cool temperate zone accumulated more C (Fig. 3a) due largely to the associated longer fertilization duration – 26 yr, compared with 12 and 16 yr for tropical and warm temperate regions, respectively. And this was supported by evidences from Maillard and Angers^15^ and Tian *et al*.^31^, both confirming that cool temperate accumulated more C than tropical. However, the relative changes under the four treatments were generally lowest in cool temperate and highest in tropical regions (Fig. 3b), which might be caused by different initial SOC conditions (i.e., 15.8, 11.6 and 7.8 g kg^−1^ in cool temperate, warm temperate and tropical regions, respectively). The RR was generally negatively correlated with initial SOC (Fig. S11b,e,h), which was also supported by Liu *et al*.^14^ and Maillard and Angers^15^, suggesting the effects of initial SOC on the SOC changes. Unlike the ΔSOC change, the rates of SOC change were higher in tropical and warm temperate regions than in cool temperate areas under all treatments (Fig. 4b), which could be attributed to the shorter durations^28^−^31^,^33^. The SOC change rate was negatively correlated with initial SOC but was not statistically significant during the 0–10 yr (Fig. S11c). For the 10–20 yr and 20–40 yr periods, however, the SOC change rate showed an increasing trend with initial SOC levels (Fig. S11c). This was partly because the effect of initial SOC on SOC changes declined with duration (Fig. S11c,f,i), and at the same time, there was positive correlation between the C input and the initial SOC levels in the dataset we collected. For example, in the 20–40 yr duration subgroup (Fig. S11i), the C input for the 0–5, 5–15 and >15 g kg^−1^ initial SOC subgroups was 4.4, 5.0 and 5.2 Mg ha^−1^ yr^−1^, respectively. Assuming that the mean SOC increased 1.7–3.5 g kg^−1^ as suggested by CF and CFM treatment, the C sink potential would be 9.1–21.4 Pg for 1370 M ha cropland soils to a depth of 30 cm (assuming a bulk density of 1.3 g cm^−3^).

Our results suggested that, on average, the relative SOC increase under CFM was 36.2% (Table 1) over 22 yr. Similarly, Maillard and Angers^15^ estimated it to be 26% for 18 yr (or 31.8% for 22 yr). While in Mediterranean cropping systems, Aguilera *et al*.^32^ reported a higher increase of 26–48% under organic amendments for 6–8 yr. The SOC increased the most under CFM not only because it directly adds organic matter (OM) into soil, but also it contains N and P, and the slow release of the nutrient may apply additional fertilizer and increase C input from crops^35^,^36^. The relative SOC increase under CFS was 19.4% for 11 yr in the present analysis, while Liu *et al*.^14^ reported it as 12.8% without providing the duration. Zhao *et al*.^29^ reported increases of 10.0% and 17.0% after 3–5 yr and >15 yr of straw incorporation in China. Similarly, Powlson *et al*.^33^ estimated an increase of ~17.0% for 13 yr in the Indo-Gangetic Plains and ~34.2% for 6 yr in Sub-Sharan Africa under CFS. These differences were probably caused by differences in the durations (from 6 yr to >15 yr), initial SOC concentrations (from 6.1 g kg^−1^ in Sub-Sharan Africa to 10.8 g kg^−1^ for the mean global croplands) and climatic conditions resulting from different site distributions^14^,^30^,^33^. Chemical fertilizers only increased SOC by 10.0–15.5% in this study. Because chemical fertilizers did not directly incorporate organic matter into soil as CFM and CFS did, they only enhanced C input by increasing root and stubble biomass retention, thus resulting in a lower increase than CFM and CFS^28^−^32^. Recent estimates of the relative SOC change rates for manure application and straw retention were 0.17–0.34 and 0.20–0.30 g kg^−1^ yr^−1^, respectively^28^,^31^, while chemical fertilizer application yielded the much slower rate of 0.07–0.15 g kg^−1^ yr^−1^ 29,31. Our results agreed well with their estimates (Table 1).

We found a weak but significant correlation between the rate of SOC change and the experiment duration (Fig. 6), with R^2^ ranging from 0.08–0.29. Similar results have been reported in other studies^14^,^28^,^29^,^30^,^31^. These results were mainly due to large disparities in rates of SOC change caused by differences in rates of C input (from 0.3 to over 10 Mg ha^−1^ yr^−1^, Table S1), climate conditions (MAT ranges from 3.6 °C to 27.0 °C, and MAP ranges from 120 mm to 1698 mm) and initial SOC concentrations (2.0–105.0 g kg^−1^)^14^,^15^,^32^ (Table S1, Fig. 6, Fig. S11). We obtained the C sequestration durations across climate regions under four treatments (Table 1, Table S2). For the manure treatment, the duration of C sequestration was 50–65 yr for warm temperate regions in this study. Similarly, it was estimated as 45–64 yr in paddy fields in China^29^,^31^, and for cool temperate croplands, it was estimated as much longer (72–117 yr), which is supported by long-term experiments. The Rothamsted experiment showed that SOC was still increasing after 150 yr of manure application^9^. Experiments from another site in Ultuna, Sweden, also showed an increase of SOC after 54 yr of manure application^11^. The longer C equilibrium time was because of the slow C turnover rates in cool temperate regions (Fig. 4b and Fig. S7). Straw return followed the same trend as the manure application. Here, we reached an estimate of 28–73 yr under straw return on a global scale (Table 1). Powlson *et al*.^33^ estimated C sequestration duration in the Indo-Gangetic Plains and Sub-Sharan Africa under CFS to be 15–30 yr, which overlapped with our estimates in tropical (Table S2).Tian *et al*.^31^ reported the duration of C sequestration as 35–40 yr in China, which falls into the range of our results. Zhao *et al*.^30^ reported a similar 15–30 yr under straw incorporation in China. Two long-term experiments showed that C sequestration lasted 20–54 yr^11^,^12^, whereas Liu *et al*.^14^ suggested a much shorter sequestration time (12–15 yr) based on linear regressions. The duration of C sequestration under chemical fertilizer applications was estimated as 20–46 yr in temperate China^28^,^30^, while we identified the wider range of 19–69 yr for temperate and tropical regions (Table 1, Table S2). Our global synthesis suggests that SOC models need to treat soil C sequestration duration differently depending on the climatic regions.

**Figure 7:**
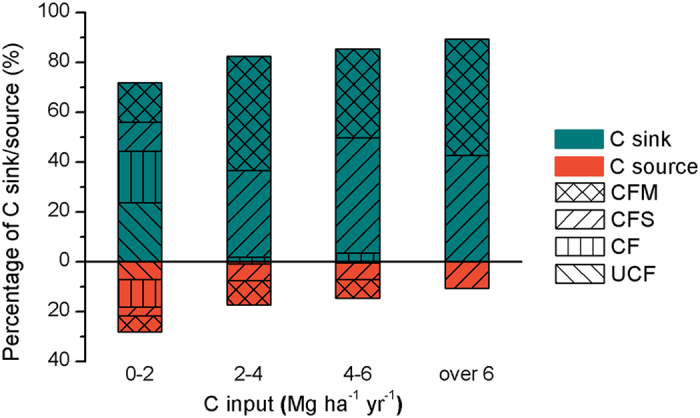
Percentages of treatments producing C sinks and C sources among the total treatments in different C input groups. When the difference between SOC in the final year and SOC in the initial year is bigger than 0, the soil is a C sink, and the opposite is a C source.

Changes in management practices to increase SOC must increase organic matter inputs, decrease decomposition of SOM, or use a combination of these approaches^8^. Both the quantity and the quality of added OM significantly influenced the efficiency of C sequestration^37^. Both linear^15^,^38^,^39^,^40^ and nonlinear^41^,^42^ relationships were found between changes in SOC and the amount of organic C added in long-term field experiments and meta-analyses. In this study, the ΔSOC increased linearly with C input in the 10–20 yr, 20–30 yr and 30–40 yr groups (Fig. 3a). Meanwhile, in the 0–10 yr and >40 yr groups, nonlinear increases were observed. When C input increased from 0–2 to over 6 Mg ha^−1^ yr^−1^, the percentage of C sinks increased from 72–89% (Fig. 7). Under medium soil fertility (mean SOC of 12.0 g kg^−1^ in this study), at least 2.0 Mg ha^−1^ yr^−1^ C input is needed to maintain the SOC in most cases (~85%), which coincided with a recent estimation using the RothC model to maintain current SOC in global wheat systems^43^. The efficiency of soil C sequestration by manure was higher than that for straw, but this difference was not significant across most climate regions (Fig. 5). This result might be due to the limited samples, and it became significant when multiple-year observations were used (Fig. S9).

N addition is another significant factor that influences SOC changes (Fig. S12). Intensive use of N fertilizers in the Green Revolution is motivated by the economic value of high grain yields^44^−^46^ and is generally considered to sequester SOC by increasing the crop residues input^14^,^30^,^33^. As expected, ΔSOC, RR and the relative SOC change rate (RCR) were higher under N treatment than without N addition (p = 0.22, 0.03 and 0.38, Fig. S12). In contrast, the phosphorous alone treatment yielded the smallest ΔSOC, RR and SOC change rates (Fig. S12). N addition increased aboveground litter input by 20% while decreased microbial respiration by 8% in natural ecosystems, and both of them contributed to SOC sequestration^47^. While it was difficult to predict the response of long term C sequestration in natural ecosystems under N addition^47^, it was proved to increase SOC by 8.0–15.4% in the agro-ecosystems in this study (Fig. 3d, Fig. S12b). Similarly, N addition resulted in a significant 3.5% increase in SOC in agricultural ecosystems, but no significant change in forests and grasslands^27^. The corresponding increase was 8.0% under N addition in this study (Fig. S12b). Chemical fertilizers and straw return also interacted with each other. Straw mulching significantly enhanced nitrogen use efficiencies and yields of maize and wheat, and thus indirectly enhanced the C input^48^.

Publication bias was evaluated using the Kendall’s tau rank and Spearman rank^48^−^50^ (Table S4). It should be noted that publication bias was detected under CFM treatment in cool temperate and in the 0–10 yr subgroup. Therefore, such estimates should be used cautiously when applying them to estimate the real soil C sequestration.

The findings reported here might be useful to improve cropland surface models in terms of SOC changes subjected to chemical fertilization, straw and manure application, e.g., the C sequestration duration across climate zones should be treated differently in models. The implications were also important for the cropland management, especially for the developing areas where soils are suffering C loss. Our synthesis suggested that the RMPs should last more than decades to achieve the potential C sink and could not go back to the old routines, or the sequestered C might be lost again^1^. The future research should pay more attention to SOC changes in the developing areas where observations of long-term SOC dynamics are insufficient^24^,^51^, and the improvement of SOC models based on observed SOC trends is highly needed^52^,^53^.

We conducted a global meta-analysis of the effects of fertilization on changes in SOC and RR, but there were a few unavoidable limitations. First, ~41% of the total experiment sites were in China, which reduced the spatial representativeness to a certain degree. We kept these sites because China covers a wide variety of climatic, edaphic and management conditions. China is a country with a long history of agriculture emphasizing manure applications, and straw return is becoming increasingly popular with the promotion of straw return and ban on straw burning by the government. Therefore, incorporating these data into the analysis was necessary and would compensate for the lack of data in other developing countries. Second, meta-analyses include some basic assumptions, such as the normal distribution of data and the independence of the effect size measures. Resampling methods relax some of these assumptions^54^,^55^. We excluded the multiple-year data from individual sites for ΔSOC and RR analyses. However, our analyses were not completely independent because many papers reported data for multiple variations of one fertilizer treatment (e.g., different application rates of chemical fertilizer, straw or manure, or different crop rotations). Additionally, in many experiments for a given site, data from various kinds of fertilizers and one fertilizer at different doses were kept, and all of them were compared to a single control. However, the effects of fertilizations did not change: the 95% CI are smaller when all data are used compared with the results obtained using only a part of the data^31^,^56^, and this was also confirmed by this study (Table S5). Third, the lack of SD, SE or sample size makes it impossible to calculate the weight for each RR, and thus an unweighted meta-analysis was used. Additionally, the SOC stock change cannot be estimated due to the lack of bulk density in most of the literature (~70%), which seriously hampered the understanding of different fertilization effects on SOC changes. Therefore, further experimental studies should provide sufficient data on such information.

## Methods

### Data collection

We searched the ISI Web of Science (http://apps.webofknowledge.com/) and China National Knowledge Infrastructure (http://www.cnki.net/) for articles with the terms “soil AND carbon AND (cropland OR agricultural OR fertilization OR chemical fertilizer OR straw OR residue OR manure)” in the title, abstract or keywords. The total papers found by such search were refined using the following criteria:

1. The study was conducted in the field and had a detailed description of the study site and the fertilization treatments.
2. The literature reported the SOC or soil organic matter (SOM) under control (i.e., with no fertilization) and treatment (i.e., with chemical fertilizer, manure application and/or residue retention) at the end of the experiment. All other experimental conditions were identical for the control and the treatment.
3. The literature recorded the experiment duration, and the duration was longer than one year.

In total, we obtained 238 published literatures comprising of 1741 comparisons at 298 sites (see Fig. S1 and Table S1 for details). We categorized the management practices into five groups: 1) no fertilization (CK); 2) unbalanced application of one or two types of chemical fertilizers, i.e., nitrogen, phosphorous (P) and potassium (K) (UCF); 3) balanced chemical fertilization with N, P and K (CF); 4) straw retention and application of chemical fertilizers (CFS); 5) application of manure and chemical fertilizers (CFM). The locations of different experimental sites were categorized into three geographical groups according to the climate conditions (i.e., cool temperate, warm temperate and tropical) using the world map of IPCC climate zones^57^. The experiment durations were divided into four groups: 1) 0–10 years (yr); 2) 10–20 yr; 3) 20–40 yr; 4) >40 yr. For studies that only reported the SOM data, it was converted to SOC using the van Bemmelen factor of 0.58^58^. The C input data was either extracted directly from the literature or calculated using documented straw/manure application rates and their dry matter and C fractions.

Because studies used in a meta-analysis should be independent^54^, if an individual study consisted of multiple years of observations for the same plot, we selected only the last sample^27^,^31^,^47^. If more than one level of fertilizer application or straw retention was conducted at the same site, measurements from different application rates were considered independent observations to evaluate the effects of fertilizer management on C dynamics^27^,^47^.

### Data analysis

We selected two indexes to estimate the effects of fertilizer managements on SOC dynamics: the mean difference between SOC under treatment and that under CK (ΔSOC, g kg^−1^, i.e., absolute change), and the response ratio of SOC between treatment and CK (RR, i.e., relative change). These two indexes have both been widely used as measures of experimental effect^14^,^15^,^24^,^47^,^55^. They were calculated as follows:


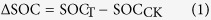



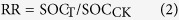


where SOC_T_ and SOC_CK_ are mean SOC contents (in g kg^−1^) under a treatment and CK in an experiment.

It is preferable to conduct analyses in the form of the natural logarithm of the response ratio (lnRR) for two reasons^55^. One is that the logarithm linearizes the metric, thus making the same deviations in the numerator and the denominator. The other is that the distribution of lnRR is more normal than that of RR, which complies better with the basic assumptions of meta-analysis^54^,^55^. The lnRR was calculated by equation (3)^55^. The lnRR was transformed back to RR in the results because its meaning could be easily understood.


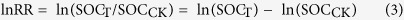


An accurate meta-analysis requires means, standard deviations (or standard errors) and the number of replicates^55^,^59^. However, only ~30% of independent treatments in the present dataset reported these values. To include as many studies as possible, an unweighted meta-analysis was used by assigning the weight for each study to be 1^48^,^49^,^60^,^61^. For each group (e.g., fertilization, climate zone and duration), the average ΔSOC and mean effect size (RR), as well as their 95% CI, were calculated using the bootstrapping (9,999 iterations) method on Metawin 2.1 software^49^,^62^. Random effects models in the meta-analysis were used to compare differences among groups^63^,^64^ (Table S3). The changes in ΔSOC and RR were considered significant if the 95% CI did not overlap 0 and 1, respectively^49^,^64^,^65^. We tested each of the fertilization groups separately using a chi-square test to determine whether total heterogeneity (Q_T_) among the effect sizes under fertilization was significantly larger than the expected sampling error^31^,^49^ (See Table S3 for details). For each group, Q_T_ was calculated as the sum of within-group heterogeneity (Q_W_) and Q_B_^55^. If the Q_B_ is significant according to the Q-statistic, the ΔSOC and RRs are considered significantly different among the groups^49^. We also performed the weighted analysis using a maximum SD (i.e., 10% of the observed SOC)^24^,^31^,^59^ as a substitute for the unknown SD based on the reported 30% SD samples, using the procedure reported by Hedges *et al*.^55^. The weighted 95% CI were very similar to the unweighted ones, and their mean differences of RR and ΔSOC were 0.47% and 1.53%, respectively (See Table S6).

The relative SOC increase in percentage (ΔSOC_P_) was calculated using equation (4), and this is another index that is widely used and can be easily calculated from RR:


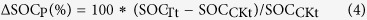


where SOC_Tt_ and SOC_CKt_ represent the mean SOC under treatment and CK in year t.

The relative SOC change rate can represent the SOC dynamics as affected by a certain treatment over time^24^,^28^,^29^,^30^,^31^,^32^,^33^, which was calculated using equation (5):


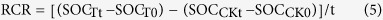


where SOC_Tt_ and SOC_CKt_ are the same as in equation (4), SOC_T0_ and SOC_CK0_ represent the initial SOC under treatment and CK, respectively, and t is the duration of fertilization.

To quantify and compare the C sequestration efficiency of manure application and straw return over time, we calculated the SOC change rate per Mg of C input (RCRC):


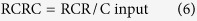


where RCR is the relative SOC change rate and C input is the quantity of C input.

Following Tian *et al*.^31^, we estimated the C sequestration duration under a certain treatment using a regression function (7):


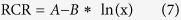


where RCR is the relative rate of SOC change, x is the duration of fertilization, and A and B are regressed constants. When RCR is equal to zero or a lower limit (set to 0.05 g kg^−1^ yr^−1^, ~0.1 Mg C ha^−1^ yr^−1^) that is difficult to detect in field experiments, the calculated x is considered the duration of C sequestration.

## Additional Information

**How to cite this article**: Han, P. *et al*. Changes in soil organic carbon in croplands subjected to fertilizer management: a global meta-analysis. *Sci. Rep.* **6**, 27199; doi: 10.1038/srep27199 (2016).

## Supplementary Material

Supplementary Information

## Figures and Tables

**Table 1:** Comparison with other studies on SOC change and durations of C sequestration.

**Treatment**	**Study region**	**Sample size**	**ΔSOC**_**P**_ **(%)**	**ΔSOC (g kg**^**−1**^)	**RCR (g kg**^**−1**^ **yr**^**−1**^)	**Experiment duration (yr)**	**C sequestration duration (yr)**	**Reference**
CFM	Global	130	26.0	5.0	0.28	18	–	Maillard and Angers^15^
	Mediterranean croplands	93	26–48	–	–	7.4	–	Aguilera *et al*.^32^
	China	201	–	–	0.34	–	45–51	Tian *et al*.^31^
	South China	145	–	5.1	0.31	16	55–64	Zhu *et al*.^29^
	Yangtze Delta Plain, China	27	–	–	0.17	–	40	Rui and Zhang^28^
	Global	652	36.2	6.5	0.29	22	26–117	This study
CFS	Global	343	12.8	–	–	–	12-15	Liu *et al*.^14^
	Mediterranean croplands	13	16.8	–	–	10.6	–	Aguilera *et al*.^32^
	the Indo–Gangetic Plains	19	17.0	1.2–3.7		12.8	–	Powlson *et al*.^33^
	Sub–Sharan Africa	21	34.2	1.0–3.7		5.7	–	Powlson *et al*.^33^
	China	105	–	11.0	0.30	37	34–40	Tian *et al*.^31^
	China	159	10.0–17.0	–	–	–	20–40	Zhao *et al*.^30^
	Yangtze Delta Plain, China	52	–	–	0.20	–	20	Rui and Zhang^28^
	Global	620	19.4	3.8	0.36	10.2	28–73	This study
CF	South China	85	–	–	0.15	–	46	Zhu *et al*.^29^
	China	163	–	4.3	0.14	31	28–34	Tian *et al*.^31^
	Global	262	15.5	3.3	0.12	27	19–69	This study
UCF	South China	44	–	1.3	0.08	17	23–28	Zhu *et al*.^29^
	China	113	–	2.4	0.10	24	20–28	Tian *et al*.^31^
	Global	340	3.5	–	–	–	–	Lu *et al*.^27^
	Global	207	10.0	1.3	0.07	18	18–58	This study

UCF, CF, CFS and CFM represent the unbalanced application of chemical fertilizers, balanced application of chemical fertilizers, straw return and application of chemical fertilizers, and application of manure and chemical fertilizers. “–” denotes not available.
