# Corrigendum: Changes in soil organic carbon in croplands subjected to fertilizer management: a global meta-analysis

**DOI:** 10.1038/srep29330

**Published:** 2016-07-08

**Authors:** Pengfei Han, Wen Zhang, Guocheng Wang, Wenjuan Sun, Yao Huang

Scientific Reports 6: Article number: 27199; 10.1038/srep27199Published online: 06022016; Updated: 07082016

In the original version of this Article, Yao Huang was incorrectly affiliated with ‘University of Chinese Academy of Sciences, Beijing 100049, China’. The correct affiliation is listed below.

State Key Laboratory of Vegetation and Environmental Change, Institute of Botany, Chinese Academy of Sciences, Beijing 100093, China.

This error has now been corrected in the PDF and HTML versions of the Article.

