# Supplementary material for: Changes in soil organic carbon in croplands subjected to fertilizer management: a global meta-analysis
Source: Sci Rep. 2016 Jun 2;6:27199. doi: 10.1038/srep27199 (PMC4890177; doi:10.1038/srep27199)
Supplement: Supplementary Information [file srep27199-s1.pdf]

# Changes in soil organic carbon in croplands subjected to fertilizer management: a global meta-analysis

Pengfei Han[1,3], Wen Zhang[1*], Guocheng Wang[1], Wenjuan Sun[2] & Yao Huang[2*]

[1] LAPC, Institute of Atmospheric Physics, Chinese Academy of Sciences, Beijing 100029, China

[2] State Key Laboratory of Vegetation and Environmental Change, Institute of Botany, Chinese Academy of Sciences, Beijing 100093, China

[3] University of Chinese Academy of Sciences, Beijing 100049, China

[*]Corresponding author:

Wen Zhang, Institute of Atmospheric Physics, Chinese Academy of Sciences, Beijing 100029, China. E-mail: zhw@mail.iap.ac.cn

Yao Huang, Institute of Botany, Chinese Academy of Sciences, Beijing 100093, China. E-mail: huangyao@ibcas.ac.cn

# 1. The information of experiment sites that used in this meta-analysis

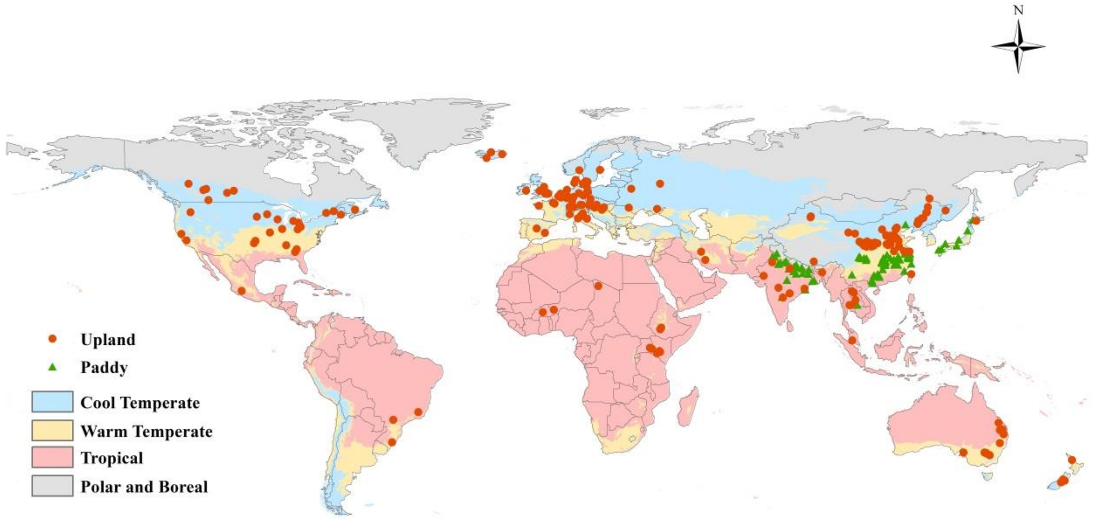


**Figure S1** Site distributions of the four management practices used in the meta-analysis (99 sites in Cool Temperate; 132 sites in Warm Temperate and 67 sites in Tropical). Map constructed in ESRI ArcMAP 10.0. Base image is obtained from the European Soil Data Center (ESDAC) (http://eusoils.jrc.ec.europa.eu/projects/RenewableEnergy/, accessed 15 July 2015).

**Table S1** The information of experiment sites that used in this meta-analysis. The minus sign before latitude and longitude denote south and west, while the others denote north and east. CT, WT and TR represent cool temperate, warm temperate and tropical regions, respectively. UCF, CF, CFS and CFM represent the unbalanced application of chemical fertilizers, balanced application of chemical fertilizers, straw and chemical fertilizers application, and manure and chemical fertilizers application, respectively, hereafter the same.

| Country | Latitude | Longitude | Climatic Zone | Initial SOC (g kg$^{-1}$) | Duration of the experiment (years) | Category of fertilization | C input (Mg C ha$^{-1}$ yr$^{-1}$) | Reference |
|---|---|---|---|---|---|---|---|---|
| Australia | -34.50 | 146.21 | WT | | 8 | CFM | | Kirkby (2006) |
| Australia | -34.58 | 146.36 | WT | | 8 | CFS | | Kirkby (2006) |
| Australia | -34.42 | 146.29 | WT | | 8 | CFS | | Kirkby (2006) |
| Australia | -34.29 | 145.80 | WT | | 8 | CFS | | Kirkby (2006) |
| Australia | -28.22 | 152.10 | WT | | 28 | UCF,CFS | | Page *et al.* (2014) |
| Australia | -24.39 | 150.51 | TR | 17.6 | 27 | CFS | | Page *et al.* (2014) |
| Australia | -26.65 | 151.84 | WT | 15.3 | 17 | CFS | | Page *et al.* (2014) |
| Australia | -35.08 | 147.33 | WT | 9.2 | 26 | UCF,CFS | | Chan *et al.* (2011) |
| Australia | -26.80 | 150.90 | TR | 6.8 | 8 | CFS | 2.0 | Wang *et al.* (2013) |
| Australia | -31.11 | 150.93 | WT | 12.8 | 24 | CFS | 1.6 | Coleman *et al.* (1997) |
| Australia | -34.30 | 138.80 | WT | 12.6 | 18 | CFS | | Wang *et al.* (2013) |
| Australia | -35.20 | 147.50 | WT | 8.0 | 11 | CFS | | Wang *et al.* (2013) |
| Austria | 47.96 | 14.06 | CT | | 21,37 | CF,CFM | 0.4,1.4 | Dersch and Böhm (2001) |
| Austria | 47.21 | 15.95 | CT | | 21,37 | CF,CFM | 1.7,2.8 | Dersch and Böhm (2001) |
| Austria | 48.23 | 16.57 | WT | | 10,17 | CF,CFS | 0.6,4.8 | Dersch and Böhm (2001) |
| Austria | 48.34 | 14.18 | CT | 11.0 | 13 | CFM | 2.8 | Ros *et al.* (2006) |
| Belarus | 53.50 | 28.10 | CT | 12.3 | 14 | CFM | 2.6 | Franko *et al.* (2007) |

| | | | | | | | | |
|---|---|---|---|---|---|---|---|---|
| Republic | | | | | | | | |
| Belgium | 50.98 | 3.82 | WT | 15.6 | 4 | CFM | 3.8 | Nevens (2003) |
| Brazil | -20.80 | -42.90 | TR | 13.7 | 26 | CFS | | Leite *et al.* (2004) |
| Brazil | -23.38 | -51.18 | TR | | 21 | CFS | | Machado *et al.* (2003) |
| Brazil | -30.90 | -51.60 | TR | 14.9 | 19 | CFS | 6.9 | Bayer *et al.* (2006) |
| Burkina Faso | 12.42 | -1.35 | TR | 4.7 | 2 | UCF,CFS,CFM | 0.5,4.2,1.5 | Ouédraogo *et al.* (2006) |
| Canada | 52.15 | -106.58 | CT | 20.1 | 10 | CF,CFS | 2.3,4.8 | Campbell *et al.* (1998) |
| Canada | 45.50 | -73.58 | CT | 24.4 | 17 | CFS | 3.9 | Halpern *et al.* (2010) |
| Canada | 52.87 | -104.33 | CT | 29.0 | 4 | UCF,CFS | 0.3,1.7 | Malhi and Lemke (2007) |
| Canada | 53.12 | -114.47 | CT | 13.1 | 28 | UCF,CFS | 1.1,2.1 | Malhi *et al.* (2011) |
| Canada | 53.42 | -113.55 | CT | | 27 | CFS | 2.1 | Chung *et al.* (2010) |
| Canada | 46.60 | -63.96 | CT | 60.9 | 28 | UCF | 1.3,1.9 | Malhi *et al.* (2011) |
| Canada | 55.22 | -119.40 | CT | 33.5 | 12 | CF,CFS,CFM | | Soon (1998) |
| Canada | 46.08 | -71.03 | CT | | 20 | CFM | 3.6 | Angers *et al.* (2010) |
| Canada | 49.70 | -112.80 | CT | 14.8 | 80 | CFS | | Monreal and Janzen 1993 |
| China | 45.70 | 126.60 | CT | 12.9 | 29 | CF,CFM | 0.9,1.5 | Xu *et al.* (2012) |
| China | 35.20 | 107.70 | WT | 6.5 | 18 | UCF,CFM | 2.0,5.8 | Guo *et al.* (2008) |
| China | 35.00 | 114.40 | WT | 4.5 | 19 | UCF,CF,CFM | 1.0,1.2,6.5 | Yu *et al.* (2012) |
| China | 29.80 | 106.40 | TR | 12.8 | 12 | UCF,CF,CFS,CFM | 0.9,1.5,3.5,4.9 | Pan *et al.* (2011) |
| China | 28.60 | 121.40 | WT | 18.7 | 26 | CF,CFM | 2.9,5.8 | Wang *et al.* (2002) |
| China | 28.30 | 116.30 | TR | 16.2 | 26 | CF,CFM | 0.5,3.2 | Bi *et al.* (2009) |
| China | 28.27 | 112.82 | WT | 20.1 | 25 | UCF,CF,CFS,CFM | 1.0,1.2,2.8,2.4 | Jun *et al.* (2007) |
| China | 28.27 | 115.84 | TR | 14.8 | 21 | UCF,CF,CFM | 0.8,1.2,3.0 | Pan *et al.* (2011) |
| China | 30.50 | 105.60 | WT | 9.2 | 17 | UCF, CF,CFM | 0.9,1.2,4.1 | Sun *et al.* (2006) |
| China | 30.50 | 114.40 | WT | 15.9 | 22 | UCF, CF,CFM | 0.8,0.9,4.8 | Hu *et al.* (2010) |
| China | 34.28 | 117.29 | WT | 6.3 | 19 | UCF, CF,CFM | 1.1,1.4,7.3 | Zhang and Zhang (2001) |

| | | | | | | | | |
|---|---|---|---|---|---|---|---|---|
| China | 38.93 | 100.45 | CT | 12.1 | 23 | UCF, CF,CFM | 0.8,1.4,3.7 | Jiang *et al.* (2014) |
| China | 45.67 | 126.58 | CT | 15.4 | 24 | UCF, CF,CFM | 0.9,0.9,2.9 | Xu *et al.* (2012) |
| China | 37.97 | 113.11 | CT | 13.8 | 10 | UCF,CFS,CFM | 0.6,3.3,3.3 | Jia *et al.* (2008) |
| China | 37.88 | 115.70 | WT | 6.7 | 26 | UCF,CFS,CFM | 0.8,1.4,1.3 | Du *et al.* (2009a) |
| China | 34.27 | 108.05 | WT | 6.7 | 21 | UCF,CFS,CFM | 2.1,11.9,6.9 | Chen *et al.* (2012) |
| China | 35.27 | 107.50 | WT | 6.2 | 30 | UCF,CFS,CFM | 0.4,2.2,1.6 | Liu *et al.* (2010) |
| China | 34.78 | 113.67 | WT | 6.7 | 18 | UCF,CF,CFS,CFM | 0.9,1.4,4.7,4.1 | Jiang *et al.* (2014) |
| China | 35.07 | 113.17 | WT | 5.8 | 1 | UCF,CF,CFM | | Chen *et al.* (2010) |
| China | 40.03 | 116.29 | WT | 9.2 | 12 | UCF,CF,CFM | 0.4,1.0,2.6 | Chen *et al.* (2010) |
| China | 31.45 | 120.42 | WT | 14.0 | 28 | CF,CFS,CFM | 1.2,3.5,3.0 | Ma *et al.* (2008) |
| China | 35.32 | 110.07 | WT | 6.5 | 29 | CFS | 0.8,5.1,5.9 | Wang *et al.* (2012) |
| China | 34.60 | 105.64 | CT | 5.3 | 27 | UCF | 0.6 | E *et al.* (2012) |
| China | 34.07 | 108.03 | WT | 6.8 | 26 | UCF,CF,CFM | 0.7,0.9,3.4 | Yang *et al.* (2011) |
| China | 35.22 | 107.76 | WT | 6.1 | 23 | UCF,CFM | 0.2,6.2 | Wei and Hao (2011) |
| China | 50.25 | 127.46 | CT | 24.5 | 29 | CF,CFM | 0.4,2.0 | Jiao *et al.* (2011) |
| China | 34.27 | 108.08 | WT | 8.1 | 11 | UCF,CFS,CFM | 0.4,9.1,15.1 | Sun *et al.* (2005) |
| China | 30.43 | 106.43 | WT | 12.8 | 12 | UCF,CF,CFS,CFM | 0.4,0.6,3.0,4.1 | Pan *et al.* (2011) |
| China | 30.43 | 119.65 | WT | 15.4 | 11 | UCF,CF,CFM | 0.4,0.9,7.7 | Pan *et al.* (2011) |
| China | 40.13 | 116.14 | WT | 7.1 | 20 | UCF,CF,CFM | 1.2,2.1,3.5 | Dong *et al.* (2010) |
| China | 35.59 | 109.26 | WT | | 3 | CFS | 4.6 | Liu *et al.* (2009) |
| China | 37.71 | 115.80 | WT | 7.0 | 7 | CFS | 4.0 | Fan and Liu (2005) |
| China | 31.92 | 119.52 | WT | 10.7 | 2 | CF,CFS | 1.3,5.9 | Zhang *et al.* (2008) |
| China | 36.16 | 117.15 | WT | 7.2 | 3 | CF,CFS | 0.6,6.9 | Tian *et al.* (2010) |
| China | 34.60 | 108.87 | WT | 9.0 | 4 | CF,CFS | 0.6,6.9 | Ding *et al.* (2014) |
| China | 34.25 | 106.50 | CT | 6.6 | 2 | CF,CFS | 0.9,6.4 | Cai *et al.* (2011) |
| China | 22.85 | 108.30 | TR | 18.2 | 2 | CFS | 2.7 | Sun *et al.* (1987) |

| | | | | | | | | |
|---|---|---|---|---|---|---|---|---|
| China | 32.63 | 120.83 | WT | 23.1 | 8 | UCF,CFM | 0.7,4.9 | Hou *et al.* (2014) |
| China | 32.72 | 118.97 | WT | 6.4 | 2 | CFS | | Wu *et al.* (2013) |
| China | 32.30 | 119.46 | WT | 8.5 | 4 | CF,CFS | 1.3,3.9 | Liu *et al.* (2006) |
| China | 37.85 | 113.08 | CT | 10.2 | 5 | CF,CFS | 0.5,1.4 | Yan *et al.* (2010) |
| China | 28.12 | 112.30 | WT | 17.9 | 21 | CF,CFS,CFM | 1.0,4.4,5.4 | Du *et al.* (2009b) |
| China | 32.01 | 120.87 | WT | 10.8 | 3 | CF,CFS | 0.7,5.7 | Duan *et al.* (2009;2012) |
| China | 31.60 | 113.28 | WT | 13.8 | 1 | CF,CFS | 0.6,2.2 | Wang *et al.* (2013) |
| China | 42.25 | 123.82 | CT | 14.9 | 7 | CFS,CFM | 2.3,2.7 | Wang *et al.* (2000) |
| China | 36.95 | 116.63 | WT | 4.6 | 2 | CFS | 3.4 | Chen *et al.* (2004) |
| China | 43.67 | 124.80 | CT | | 5 | CF,CFS | 0.7,2.2 | Zheng *et al.* (2006) |
| China | 29.92 | 115.50 | WT | 17.9 | 4 | CF,CFS | 1.4,3.4 | Li *et al.* (2011) |
| China | 35.97 | 106.66 | CT | 9.0 | 4 | CFS | 4.3 | Zhang *et al.* (2011) |
| China | 29.30 | 88.90 | CT | 11.3 | 2 | CFS | 2.3 | Cai *et al.* (2003) |
| China | 35.63 | 114.48 | WT | 8.9 | 7 | CF,CFS | 0.8,5.5 | Gao *et al.* (2012) |
| China | 45.57 | 126.37 | CT | 25.3 | 1 | CFS,CFM | 4.3,3.3 | Liu *et al.* (2014) |
| China | 30.42 | 104.55 | WT | | 4 | CFS | | Chen *et al.* (2008) |
| China | 28.77 | 115.92 | WT | 20.1 | 2 | CFS | 2.4 | Sun *et al.* (2012) |
| China | 35.48 | 107.75 | WT | 10.1 | 3 | CF,CFS | 0.3,3.6 | Wu *et al.* (2012) |
| China | 35.90 | 104.08 | CT | 12.3 | 4 | CF,CFS | 0.4,3.3 | Wu *et al.* (2012) |
| China | 42.83 | 123.73 | CT | 9.0 | 3 | CF,CFS | 0.6,4.4 | Xu *et al.* (2015) |
| China | 47.45 | 126.92 | CT | 27.5 | 8 | CF,CFS | 0.4,2.8 | Hao *et al.* (2013) |
| China | 34.33 | 108.40 | WT | 7.1 | 2 | CF,CFS | 1.0,4.0 | Zhang *et al.* (2010) |
| China | 46.31 | 132.82 | CT | 24.4 | 1 | CF,CFS | 0.7,4.8 | Dong *et al.* (2010) |
| China | 43.80 | 125.40 | CT | 18.0 | 1 | CF,CFS | 0.5,4.1 | Jiao *et al.* (2015) |
| China | 28.92 | 111.55 | WT | 13.5 | 18 | CF,CFS | | Ma *et al.* (2011) |
| China | 31.05 | 104.19 | WT | 18.2 | 6 | CF,CFS | 1.2,9.5 | Liu *et al.* (2014) |

| | | | | | | | | |
|---|---|---|---|---|---|---|---|---|
| China | 29.99 | 115.62 | WT | 16.9 | 6 | CF,CFS | 0.3,2.5 | Su (2014) |
| China | 32.50 | 119.47 | WT | 16.1 | 2 | CF,CFS | 1.4,5.4 | Han *et al.* (2012) |
| China | 35.40 | 106.42 | CT | 7.0 | 9 | CF,CFS | 0.9,3.9 | Mu *et al.* (2011) |
| China | 30.85 | 120.16 | WT | 7.0 | 4 | CF,CFS | 1.5,3.6 | Lin *et al.* (1997) |
| China | 29.98 | 117.62 | WT | 19.8 | 7 | CFS | | Liu *et al.* (2015) |
| China | 30.58 | 112.07 | WT | 15.6 | 3 | CFS | | Liu *et al.* (2015) |
| China | 30.90 | 120.74 | WT | 24.5 | 2 | CFS | 1.1,3.0 | Zhong *et al.* (2003) |
| China | 31.97 | 119.30 | WT | 4.8 | 2 | CF,CFS | 1.2,3.3 | Ma *et al.* (2010) |
| China | 39.95 | 111.65 | CT | 6.0 | 4 | CFS | 2.2 | Sun *et al.* (2009) |
| China | 34.30 | 108.02 | WT | 9.9 | 4 | CF,CFS | | Mulati *et al.* (2012) |
| China | 37.88 | 114.68 | WT | | 6 | CF,CFS ,CFM | 0.4,5.8,3.7 | Dong *et al.* (2009) |
| China | 35.33 | 110.08 | WT | 7.3 | 2 | CF,CFS ,CFM | 1.0,4.8,7.7 | Wang *et al.* (2010) |
| China | 41.91 | 123.45 | CT | 8.5 | 3 | CF,CFS ,CFM | 1.0,4.0,3.8 | Gong *et al.* (2008) |
| China | 42.93 | 123.81 | CT | 9.2 | 3 | CF,CFS ,CFM | 0.4,4.1,3.9 | Wu *et al.* (2002) |
| China | 36.58 | 114.51 | WT | 7.9 | 3 | CF,CFS ,CFM | 1.0,3.4,1.8 | Dong *et al.* (2010) |
| China | 41.82 | 123.57 | CT | 8.6 | 12 | CF,CFS ,CFM | 0.6,1.9,1.9 | Yan *et al.* (2004) |
| China | 31.46 | 120.43 | WT | 10.3 | 5 | CF,CFM | 0.8,1.8 | Wang *et al.* (2014) |
| China | 40.56 | 116.56 | CT | 9.9 | 9 | CF,CFS ,CFM | 0.6,2.5,4.3 | Zhang *et al.* (2007) |
| China | 44.28 | 87.93 | CT | 4.7 | 20 | UCF,CF,CFS ,CFM | 0.4,0.4,1.5,1.2 | Li *et al.* (2013) |
| China | 32.73 | 115.64 | WT | 9.2 | 4 | CF,CFS ,CFM | 1.2,3.6,1.2 | Li (2001) |
| China | 28.26 | 116.92 | TR | 5.6 | 16 | CF,CFS ,CFM | 1.1,2.2,6.0 | Li *et al.* (2010) |
| China | 38.62 | 102.67 | CT | 9.5 | 19 | UCF,CFS ,CFM | 0.5,3.5,4.7 | Zeng *et al.* (2008) |
| China | 38.10 | 113.00 | CT | | 12 | CFS | 2.0 | Chen *et al.* (2009) |
| China | 30.70 | 103.83 | WT | 12.3 | 5 | CFS | 3.4 | Fan *et al.* (2005) |
| China | 31.08 | 120.77 | WT | | 15 | CF,CFS ,CFM | 0.5,2.2,2.7 | Li *et al.* (2007) |

| | | | | | | | | |
|---|---|---|---|---|---|---|---|---|
| China | 41.67 | 119.47 | CT | 9.8 | 11 | CFS | 4.4 | Lou *et al.* (2011) |
| China | 36.85 | 115.02 | WT | 4.1 | 17 | UCF,CFS | 1.5,2.6 | Niu *et al.* (2011) |
| China | 26.75 | 111.87 | WT | 4.2 | 13 | CF,CFS | 0.3,1.0 | Qin *et al.* (2010) |
| China | 31.55 | 120.70 | WT | 14.0 | 25 | UCF CF,CFS,CFM | 0.9,1.0,4.0,3.0 | Shen *et al.* (2007) |
| China | 35.02 | 114.53 | WT | 6.5 | 4 | CF,CFS | 0.6,5.9 | Wang *et al.* (2011) |
| China | 31.55 | 120.63 | WT | 19.6 | 4 | CF,CFS,CFM | 0.8,1.7,1.4 | Yang *et al.* (2005) |
| China | 35.46 | 104.75 | CT | | 5 | CF,CFS | 0.5,3.0 | Bi *et al.* (2009) |
| China | 35.57 | 106.68 | CT | 5.6 | 25 | UCF,CFS,CFM | 0.3,2.1,4.2 | Zhao *et al.* (2009) |
| China | 29.23 | 111.52 | WT | 6.2 | 31 | CF,CFS | 1.2,6.3 | Zhao *et al.* (2009) |
| China | 25.10 | 121.38 | TR | 14.0 | 5 | CF,CFM | 0.6,7.7 | Lee *et al.* (2006) |
| China | 44.00 | 87.80 | CT | 8.8 | 18 | UCF,CF,CFS,CFM | 0.4,0.7,2.3,4.8 | Jiang *et al.* (2014) |
| China | 43.50 | 124.80 | CT | 13.2 | 19 | UCF,CF,CFS,CFM | 0.3,1.0,2.9,3.8 | Jiang *et al.* (2014) |
| China | 37.91 | 114.52 | WT | | 6 | CFS | 5.0 | Dong *et al.* (2009) |
| China | 37.90 | 115.20 | WT | 10.0 | 21 | CFM | 5.0 | Lei *et al.* (2005) |
| China | 35.53 | 105.07 | CT | 8.8 | 4 | CF,CFS | 0.4,4.0 | Wu *et al.* (2012) |
| China | 35.35 | 106.88 | CT | 9.3 | 4 | CF,CFS | 0.4,4.0 | Wu *et al.* (2012) |
| China | 35.08 | 105.25 | CT | 9.1 | 4 | CF,CFS | 0.4,3.6 | Wu *et al.* (2012) |
| China | 32.53 | 120.09 | WT | 5.6 | 3 | CF,CFS | 0.5,1.4 | Qian *et al.* (1998) |
| China | 32.41 | 120.11 | WT | 5.5 | 3 | CF,CFS | 0.5,1.7 | Qian *et al.* (1998) |
| China | 30.43 | 120.40 | WT | | 1 | CF,CFS | 1.3,4.7 | He *et al.* (2014) |
| China | 29.51 | 112.86 | WT | 10.2 | 2 | CF,CFS | 0.5,1.4 | Liu *et al.* (2003) |
| China | 29.20 | 112.80 | WT | 26.0 | 14 | CFM | 8.2 | Huang *et al.* (2009) |
| China | 28.80 | 111.80 | WT | 17.1 | 14 | CFM | 7.7 | Liu *et al.* (2007) |
| China | 28.25 | 116.33 | TR | 17.6 | 25 | CF,CFM | 1.1,4.7 | Yuan *et al.* (2008) |
| China | 26.23 | 119.07 | TR | 12.5 | 28 | CF,CFS,CFM | 1.1,5.6,2.8 | Li (2011) |
| China | 25.21 | 110.21 | TR | 18.4 | 1 | CFS | 3.7 | Yan (2013) |

| | | | | | | | | |
|---|---|---|---|---|---|---|---|---|
| China | 25.00 | 101.60 | WT | 31.4 | 12 | UCF,CF,CFM | 0.8,0.9,5.3 | Wang (2000) |
| China | 24.78 | 108.91 | TR | 18.8 | 1 | CFS | | Yan (2013) |
| China | 24.64 | 110.66 | TR | 18.9 | 1 | CFS | | Yan (2013) |
| China | 24.47 | 109.76 | TR | 14.7 | 1 | CFS | | Yan (2013) |
| China | 24.16 | 110.81 | TR | 18.1 | 1 | CFS | | Yan (2013) |
| China | 23.19 | 108.22 | TR | 18.4 | 1 | CFS | 2.8 | Yan (2013) |
| China | 22.14 | 108.45 | TR | 19.5 | 1 | CFS | | Yan (2013) |
| China | 30.72 | 116.86 | WT | 13.0 | 1 | CF,CFS | 1.4,2.8 | Li (2015) |
| China | 31.02 | 117.10 | WT | 19.5 | 1 | CFS | 5.8 | Tian (2012) |
| Czech Republic | 50.10 | 14.50 | CT | 10.0 | 21 | CFM | 2.1 | Coleman *et al.* (1997) |
| Denmark | 55.18 | 11.93 | CT | 17.2 | 70 | CF,CFM | | Bruun *et al.* (2003) |
| Denmark | 55.18 | 11.93 | CT | 8.5 | 66 | CF,CFM | | Bruun *et al.* (2003) |
| Denmark | 56.50 | 9.57 | CT | 23.8 | 12 | CFM | 3.4 | Chirinda *et al.* (2010) |
| Denmark | 55.46 | 9.10 | CT | | 93 | CF,CFM | | Anderson and Domsch (1989) |
| Ethiopia | 7.30 | 38.08 | TR | 3.9 | 3 | CF,CFS | 0.4,3.1 | Zeleke *et al.* (2004) |
| Ethiopia | 6.78 | 37.72 | TR | 5.5 | 3 | CF,CFS | 0.4,3.1 | Zeleke *et al.* (2004) |
| France | 48.90 | 2.00 | WT | 11.8 | 115 | CFS,CFM | 1.6,4.0 | Houot *et al.* (1989) |
| France | 47.88 | -2.73 | WT | 25.0 | 8 | CFS,CFM | 3.0,4.4 | Viaud *et al.* (2011) |
| France | 48.55 | 2.55 | WT | 12.1 | 32 | CFS | | Balesdent *et al.* (1990) |
| Germany | 51.30 | 12.10 | CT | 15.1 | 87 | CF,CFM | 2.8,3.9 | Kelly *et al.* (1997) |
| Germany | 50.90 | 6.88 | CT | | 32 | CF,CFS,CFM | 0.6,2.4,2.5 | Marschner *et al.* (2003) |
| Germany | 52.57 | 13.63 | CT | | 60 | UCF,CFM | | Anderson and Domsch (1989) |
| Germany | 52.03 | 10.63 | CT | | 25 | CF,CFS | | Anderson and Domsch (1989) |
| Germany | 50.77 | 7.16 | WT | | 14 | CF,CFS ,CFM | | Anderson and Domsch (1989) |
| Germany | 50.76 | 8.89 | CT | | 24 | UCF,CFS ,CFM | | Anderson and Domsch (1989) |
| Germany | 50.61 | 7.01 | CT | | 28 | UCF,CFS | | Anderson and Domsch (1989) |

| | | | | | | | | |
|---|---|---|---|---|---|---|---|---|
| Germany | 49.43 | 8.39 | WT | | 17 | CF,CFM | | Anderson and Domsch (1989) |
| Germany | 48.19 | 11.22 | CT | | 18 | UCF,CF,CFS,CFM | | Anderson and Domsch (1989) |
| Germany | 50.40 | 6.55 | CT | | 61 | CF,CFM | 0.8,4.4 | Körschens *et al.* (1998) |
| Germany | 52.26 | 13.27 | CT | | 54 | UCF,CF,CFS,CFM | 0.6,0.7,2.6,2.1 | Körschens *et al.* (1998) |
| Germany | 48.17 | 11.67 | CT | | 3 | CFM | 4.8 | Ebertseder *et al.* (2001) |
| Hungary | 47.32 | 19.00 | WT | 29.0 | 36 | CFM | 1.8 | Falloon and Smith (2002) |
| Hungary | 46.54 | 18.31 | WT | 25.7 | 29 | CF | 2.0 | Falloon and Smith (2002) |
| Iceland | 65.03 | -14.95 | CT | | 43 | CF | | Gudmundsson *et al.* (2008) |
| Iceland | 65.68 | -18.60 | CT | | 62 | CF | | Gudmundsson *et al.* (2008) |
| Iceland | 63.73 | -20.10 | CT | | 61 | CF | | Gudmundsson *et al.* (2008) |
| India | 23.20 | 80.00 | TR | 5.8/6.9 | 28 | UCF,CF,CFS, CFM | | Yadav et al. (2000); Hati *et al.* (2007) |
| India | 30.90 | 75.90 | TR | 3.6 | 12 | UCF,CFS,CFM | 0.8, 3.7,2.8 | Aulakh *et al.* (2001); Bhattacharyya *et al.* (2007) |
| India | 22.80 | 88.40 | TR | 7.1 | 34 | CF,CFM | | Bhattacharyya *et al.* (2007) |
| India | 23.00 | 89.00 | TR | 8.8 | 19 | CF,CFS,CFM | 0.8,4.9,4.9 | Majumder *et al.* (2008) |
| India | 30.90 | 75.13 | TR | 4.8 | 8 | UCF,CFS,CFM | 1.0,4.1,7.2 | Benbi and Senapati (2010) |
| India | 20.42 | 85.92 | TR | 4.9 | 4 | UCF,CFS,CFM | 0.5,2.4,2.8 | Bhattacharyya *et al.* (2012) |
| India | 21.15 | 17.12 | TR | 4.1 | 5 | CFS | 1.0 | Blaise and Ravindran (2003) |
| India | 29.07 | 77.77 | TR | 4.0 | 4 | UCF, CF,CFS | 0.9,1.0,3.5 | Gangwar *et al.* (2006) |
| India | 30.93 | 75.87 | TR | 3.0 | 6 | CF,CFS | 0.5,3.3 | Ghuman and Sur (2001) |
| India | 29.03 | 75.08 | TR | 4.2 | 12 | UCF, CFS,CFM | 0.6,3.7,1.2 | Goyal *et al.* (1999) |
| India | 25.30 | 83.02 | TR | 7.9 | 2 | CFS | 6.0 | Kushwaha *et al.* (2000) |
| India | 28.48 | 77.21 | TR | 7.5 | 4 | CFS | 0.6,3.3 | Prasad *et al.* (1999) |
| India | 17.30 | 78.60 | TR | 3.7 | 7 | UCF, CFS,CFM | 0.2,1.0,1.0 | Sharma *et al.* (2005) |
| India | 28.63 | 77.18 | TR | 5.9 | 4 | CF,CFS | 1.3,8.4 | Sharma *et al.* (2010) |

| | | | | | | | | |
|---|---|---|---|---|---|---|---|---|
| India | 17.53 | 78.27 | TR | 9.2 | 3 | UCF,CFS,CFM | 1.9,4.9,5.1 | Surekha *et al.* (2003) |
| India | 32.10 | 76.55 | WT | 11.4 | 5 | CFS,CFM | 2.2,3.2 | Verma and Bhagat (1992) |
| India | 29.00 | 79.08 | TR | 14.2 | 15 | CF,CFS,CFM | 0.7,6.7,1.8 | Yadav *et al.* (2000) |
| India | 26.97 | 80.57 | TR | 2.9 | 13 | CF,CFS,CFM | 0.7,4.1,1.9 | Yadav *et al.* (2000) |
| India | 26.07 | 82.13 | TR | 3.7 | 14 | CF,CFS,CFM | 0.6,4.8,1.8 | Yadav *et al.* (2000) |
| India | 25.23 | 87.07 | TR | 4.6 | 14 | CF,CFS,CFM | 0.6,4.2,2.9 | Yadav *et al.* (2000) |
| India | 25.50 | 85.25 | TR | 5.3 | 5 | CF,CFM | 0.4,2.4 | Singh *et al.* (2001) |
| India | 25.68 | 91.63 | TR | | 6 | CF,CFM | | Saha et al. (2010a) |
| India | 20.25 | 85.83 | TR | | 4 | CF,CFM | 0.4,5.1 | Saha *et al.* (2010b) |
| India | 26.93 | 80.87 | TR | 3.2 | 2 | CF,CFM | 2.9,6.7 | Singh *et al.* (2007) |
| India | 29.60 | 79.67 | WT | 5.1 | 3 | UCF,CF,CFM | 1.1,1.9,4.6 | Saha *et al.* (2008) |
| India | 20.56 | 77.14 | TR | | 19 | UCF,CFM | | Sharma *et al.* (2011) |
| India | 18.69 | 80.85 | TR | 5.6 | 3 | CF,CFM | 4.7,8.1 | Dass *et al.* (2008) |
| India | 20.00 | 86.00 | TR | 7.5 | 21 | UCF CF,CFM | 0.6,0.7,1.6 | Nayak *et al.* (2009) |
| India | 24.50 | 72.22 | TR | 3.1 | 18 | CF,CFM | 0.3,1.5 | Srinivasarao *et al.* (2014) |
| India | 28.30 | 83.05 | WT | | 9 | CFS,CFM | 2.2,1.6 | Sarkar *et al.* (2003) |
| Iran | 29.83 | 52.77 | WT | 12.5 | 4 | CFS | 2.0 | Bahrani *et al.* (2002) |
| Iran | 32.53 | 51.38 | WT | 5.0 | 8 | UCF,CFM | 0.4,11.5 | Hemmat *et al.* (2010) |
| Ireland | 52.86 | -6.94 | CT | 13.6 | 9 | UCF,CFS | 1.7,4.1 | van Groenigen *et al.* (2011) |
| Italy | 44.60 | 11.40 | WT | 7.7 | 35 | CFS,CFM | | Plaza *et al.* (2012) |
| Italy | 45.09 | 7.60 | WT | 11.6 | 11 | UCF,CFS,CFM | 0.9,6.6,3.5 | Monaco *et al.* (2008) |
| Italy | 45.35 | 11.97 | WT | 1.7-105.0 | 39 | CF,CFS,CFM | 2.1,3.4,5.6 | Morari *et al.* (2006) |
| Italy | 44.43 | 12.13 | WT | 7.0 | 6 | CFM | 3.4 | Bragato *et al.* (1998) |
| Italy | 43.67 | 10.32 | WT | | 25 | CFS | 3.3 | Mazzoncini *et al.* (2008) |
| Italy | 43.53 | 13.37 | WT | 12.4 | 45 | CFS | 6.5 | De Sanctis *et al.* (2012) |
| Japan | 36.62 | 137.23 | WT | 17.6 | 19 | CFS | 2.5 | Shirato and Yokozawa (2005) |

| | | | | | | | | |
|---|---|---|---|---|---|---|---|---|
| Japan | 35.33 | 132.73 | WT | 17.8 | 19 | CFS | 2.5 | Shirato and Yokozawa (2005) |
| Japan | 34.63 | 136.50 | WT | 19.0 | 24 | CFS | 2.3 | Shirato and Yokozawa (2005) |
| Japan | 33.53 | 131.38 | WT | 9.0 | 19 | CFS | 2.9 | Shirato and Yokozawa (2005) |
| Japan | 42.88 | 143.05 | CT | 34.0 | 5 | CF,CFS,CFM | 0.2,1.3,2.8 | Koga and Tsuji (2009) |
| Japan | 43.23 | 141.82 | CT | | 1 | CFS | 1.1 | Naser *et al.* (2007) |
| Japan | 33.25 | 130.48 | WT | | 41 | UCF,CFS | 0.5,3.2 | Tirol‐Padre *et al.* (2005) |
| Japan | 39.68 | 140.12 | WT | 21.4 | 16 | CF | 0.53 | Shirato and Yokozawa (2005) |
| Kenya | 0.57 | 34.33 | TR | 8.3 | 2 | CFS | 1.1 | Anyanzwa *et al.* (2010) |
| Kenya | -0.50 | 37.45 | TR | 29.3 | 4 | UCF,CFS | 0.3,2.1 | Gentile *et al.* (2010) |
| Kenya | -0.78 | 37.67 | TR | 5.3 | 4 | UCF,CFS | 0.3,2.1 | Gentile *et al.* (2010) |
| Kenya | 0.23 | 34.94 | WT | | 2 | CFM | 3.5 | Kimetu and Lehmann (2010) |
| Kenya | -1.25 | 36.68 | WT | 20.0 | 27 | UCF,CFS,CFM | 0.3,2.7,2.1 | Kibunja *et al.* (2010) |
| Malaysia | 3.03 | 101.70 | TR | 17.0 | 3 | CF,CFS,CFM | 0.6,2.2,5.2 | Mubarak and Rosenani (2003) |
| Mexico | 19.51 | -101.71 | WT | 17.2 | 6 | CFS,CFM | 3.1,4.0 | Roldán *et al.* (2003) |
| Mexico | 19.00 | 102.22 | TR | 7.0 | 9 | CFS | 1.8 | Salinas-Garcia *et al.* (2001) |
| Mexico | 19.42 | 101.60 | TR | 33.6 | 9 | CFS | 2.2 | Salinas-Garcia *et al.* (2001) |
| Nepal | 27.35 | 84.88 | TR | 6.5 | 20 | UCF,CF,CFS,CFM | 0.3,0.5,2.7,2.0 | Gami *et al.* (2001) |
| Nepal | 26.72 | 87.28 | TR | | 25 | CF,CFM | 0.4,3.6 | Gami *et al.* (2009) |
| Nepal | 27.52 | 83.42 | TR | | 25 | CF,CFM | 0.4,3.6 | Gami *et al.* (2009) |
| Nepal | 27.08 | 84.93 | TR | | 23 | CF,CFM | 0.4,4.0,3.6 | Gami *et al.* (2009) |
| Nepal | 27.65 | 82.35 | TR | 14.4 | 4 | UCF,CFM | 0.6,4.6 | Ghimire *et al.* (2012) |
| New Zealand | -43.63 | 172.50 | WT | 31.0 | 6 | CF,CFS | 0.5,2.6 | Curtin and Fraser (2003) |
| New Zealand | -43.78 | 172.02 | WT | | 8 | CFM | 1.8 | Murata and Goh (1997) |
| New Zealand | -43.65 | 172.48 | WT | | 5 | CFS | | Goh *et al.* (2000) |
| New Zealand | -43.31 | 171.93 | CT | | 8 | CFM | | Nguyen *et al.* (1995) |
| New Zealand | -43.54 | 172.47 | WT | | 8 | CFM | | Nguyen *et al.* (1995) |

| | | | | | | | | |
|---|---|---|---|---|---|---|---|---|
| New Zealand | -44.24 | 171.29 | WT | | 8 | CFM | | Nguyen *et al.* (1995) |
| New Zealand | -36.77 | 174.92 | WT | | 10 | CFM | | Reganold *et al.* (1993) |
| Niger | 13.25 | 2.30 | TR | 2.0 | 2 | CFS | 1.0 | Michels *et al.* (1995) |
| Norway | 59.67 | 10.77 | CT | | 49 | CF,CFM | 0.6,4.2 | Holeplass *et al.* (2004) |
| Romania | 47.22 | 27.25 | CT | | 13 | UCF,CFS, CFM | 0.4,3.1,3.5 | Ailincai *et al.* (2011) |
| Russia | 55.20 | 37.70 | CT | 6.4 | 36 | CF,CFM | 0.7,1.9 | Shevtsova *et al.* (2003) |
| Spain | 38.83 | -0.70 | WT | 11.6 | 2 | CF | 0.2,1.6 | García-Orenes *et al.* (2009) |
| Spain | 40.30 | -3.40 | WT | 5.6 | 17 | CFS | 1.2 | Hernanz *et al.* (2002) |
| Sweden | 59.82 | 17.65 | CT | 15.0 | 54 | CF,CFS,CFM | 0.8,2.2,2.8 | Kätterer *et al.* (2011) |
| Sweden | 55.98 | 12.87 | CT | 31.4 | 34 | CF,CFM | | Carlgren and Mattsson (2001) |
| Sweden | 55.82 | 13.50 | CT | 24.3 | 34 | CF,CFM | | Carlgren and Mattsson (2001) |
| Sweden | 55.63 | 13.43 | CT | 15.4 | 34 | CF,CFM | | Carlgren and Mattsson (2001) |
| Sweden | 54.40 | 13.23 | CT | 15.2 | 34 | CF,CFM | | Carlgren and Mattsson (2001) |
| Sweden | 55.88 | 12.87 | CT | 11.6 | 34 | CF,CFM | | Carlgren and Mattsson (2001) |
| Switzerland | 47.50 | 7.55 | CT | 16.3 | 29 | CF,CFM | 1.4,2.6 | Leifeld *et al.* (2009) |
| Switzerland | 47.48 | 8.91 | CT | 28.0 | 14 | CFS | 6.4 | Anken *et al.* (2004) |
| Thailand | 14.80 | 100.80 | TR | 6.5 | 28 | CF,CFS | 3.8,6.6 | Shirato *et al.* (2005) |
| Thailand | 14.87 | 101.65 | TR | 5.9 | 27 | CF,CFS | 0.7,4.5 | Shirato *et al.* (2005) |
| Thailand | 16.48 | 102.83 | TR | 5.0 | 26 | CF,CFS | 0.7,2.6 | Shirato *et al.* (2005) |
| Thailand | 14.92 | 103.42 | TR | | 12 | CF,CFS,CFM | 0.3,1.7,3.1 | Thuithaisong *et al.* (2011) |
| The Netherlands | 53.16 | 6.65 | CT | 8.6 | 20 | CFM | | Zwart (2003) |
| The Netherlands | 51.90 | 4.58 | CT | 18.6 | 29 | CFM | | Zwart (2003) |
| UK | 51.80 | 0.40 | CT | 25.5 | 116 | CF,CFM | 2.7,3.3 | Coleman *et al.* (1997) |
| UK | 52.18 | 0.11 | CT | 33.0 | 17 | CFM | 2.5 | Bhogal *et al.* (2011) |
| UK | 52.78 | -2.43 | CT | 15.0 | 17 | CFM | 2.2 | Bhogal *et al.* (2011) |
| UK | 54.13 | -0.99 | CT | 14.0 | 14 | CFM | 1.6 | Bhogal *et al.* (2011) |

| | | | | | | | | |
|---|---|---|---|---|---|---|---|---|
| UK | 53.53 | -1.28 | CT | 11.0 | 15 | CFM | 4.4 | Bhogal *et al.* (2011) |
| UK | 51.98 | -0.63 | CT | | 5 | CFM | 2.1 | Johnston *et al.* (2009) |
| UK | 51.80 | -0.37 | CT | | 124 | CF,CFM | | Haynes and Naidu (1998) |
| UK | 51.80 | -0.37 | CT | | 102 | CF,CFM | 0.3,3.7 | Powlson *et al.* (2011) |
| Ukraine | 46.80 | 36.70 | WT | 30.8 | 33 | CF,CFM | 1.1,1.8 | Franko *et al.* (2007) |
| USA | 40.10 | -88.23 | WT | | 51 | CFM | | Nafziger and Dunker (2011) |
| USA | 50.57 | 4.68 | CT | | 36 | CFS,CFM | 1.7,2.1 | Davis *et al.* (2003) |
| USA | 35.40 | -97.60 | WT | 15.0 | 12 | CFS | | Dao (1998) |
| USA | 40.00 | -83.02 | WT | | 9 | CFS | 4.3 | Duiker and Lal (1999) |
| USA | 36.34 | -120.12 | WT | | 6 | CFS | 2.3 | Veenstra *et al.* (2007) |
| USA | 33.37 | -83.40 | WT | | 12 | CFS | | Franzluebbers and Stuedemann (2009) |
| USA | 34.68 | -86.87 | WT | 11.5 | 10 | CFS,CFM | 6.3,8.2 | Sainju *et al.* (2008) |
| USA | 43.33 | -89.72 | CT | | 4 | CFM | 3.5 | Jokela *et al.* (2009) |
| USA | 45.70 | -118.78 | WT | | 56 | UCF,CFS,CFM | 1.8,2.0,3.6 | Rasmussen and Parton (1994) |
| USA | 44.17 | -96.68 | CT | 25.4 | 4 | UCF,CFM | 1.3,5.2 | Lee *et al.* (2007) |
| USA | 42.78 | -84.47 | CT | | 34 | CF,CFM | 0.8,5.2 | Edmeades (2003) |
| USA | 44.88 | -68.69 | CT | | 6 | CFM | 1.7 | Grandy *et al.* (2002) |
| USA | 38.94 | -92.31 | WT | | 101 | CF,CFM | 0.6,1.9 | Buyanovsky and Wagner (1998) |
| USA | 45.00 | -93.20 | CT | 28.5 | 14 | CFS | | Clapp *et al.* (2000) |
| USA | 42.22 | -82.73 | CT | 20.0 | 10 | CFS | | Jokela *et al.* (2009) |
| USA | 40.80 | -82.03 | CT | | 12 | CFS | | Dick *et al.* (1998) |
| USA | 38.50 | -121.80 | WT | 7.9 | 13 | CF,CFM | 1.1,5.2 | Doane and Horwáth (2004) |
| USA | 36.10 | -97.10 | WT | 20.8 | 111 | UCF,CF,CFM | 0.4,0.7,2.2 | Parham et al. (2002);Girma *et al.* (2008) |
| USA | 32.50 | -83.90 | WT | 8.6 | 6 | UCF,CFS | | Sainju *et al.* (2002) |

# 2. Results using multiple year observations

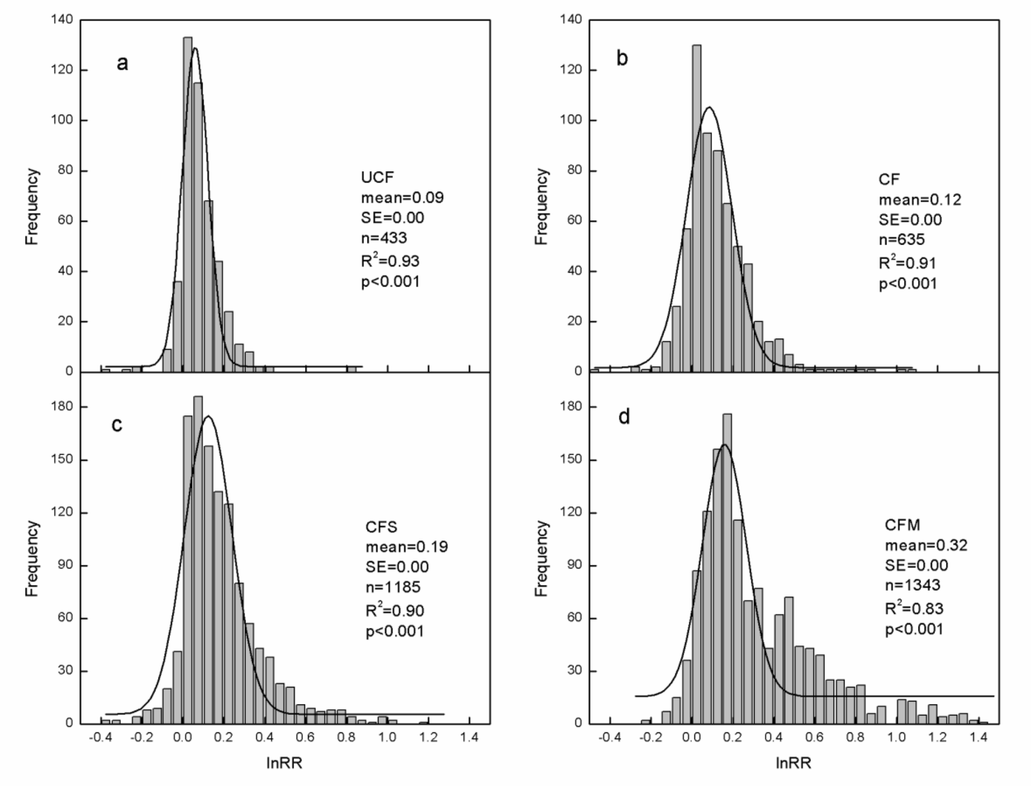


**Figure S2** Frequency distributions of response ratios (lnRR) for UCF, CF, CFS and CFM. UCF, CF, CFS and CFM represent the unbalanced application of chemical fertilizer, balanced application of chemical fertilizer, chemical fertilizer plus straw retention and chemical fertilizer plus manure, hereafter the same.

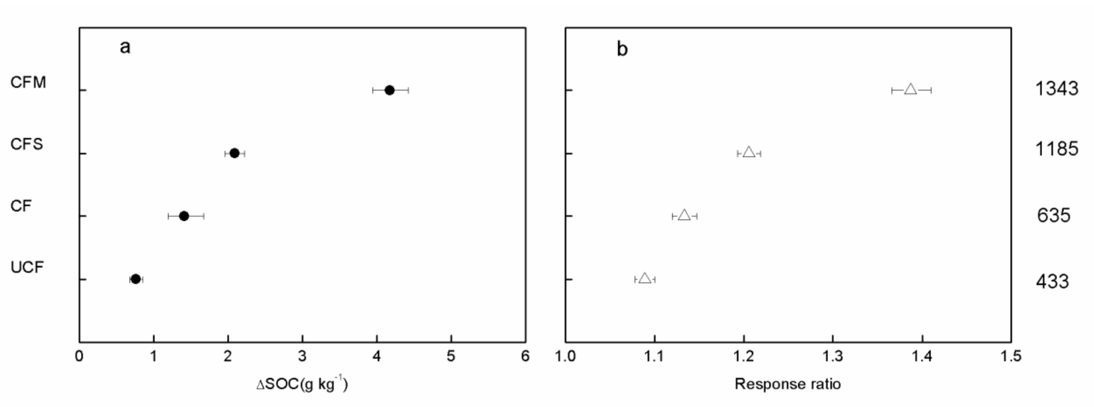


**Figure S3** Mean difference in SOC (g $kg^{-1}$) (a) and the relative change (b) comparing with CK.

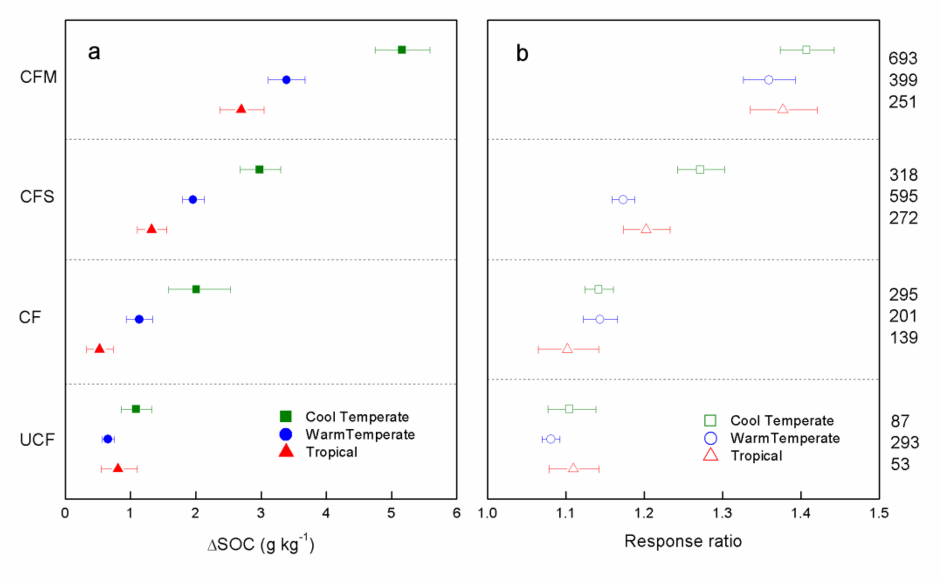


**Figure S4** Climate effects on mean difference in SOC (g $kg^{-1}$) (a) and the relative change (b) comparing with CK.

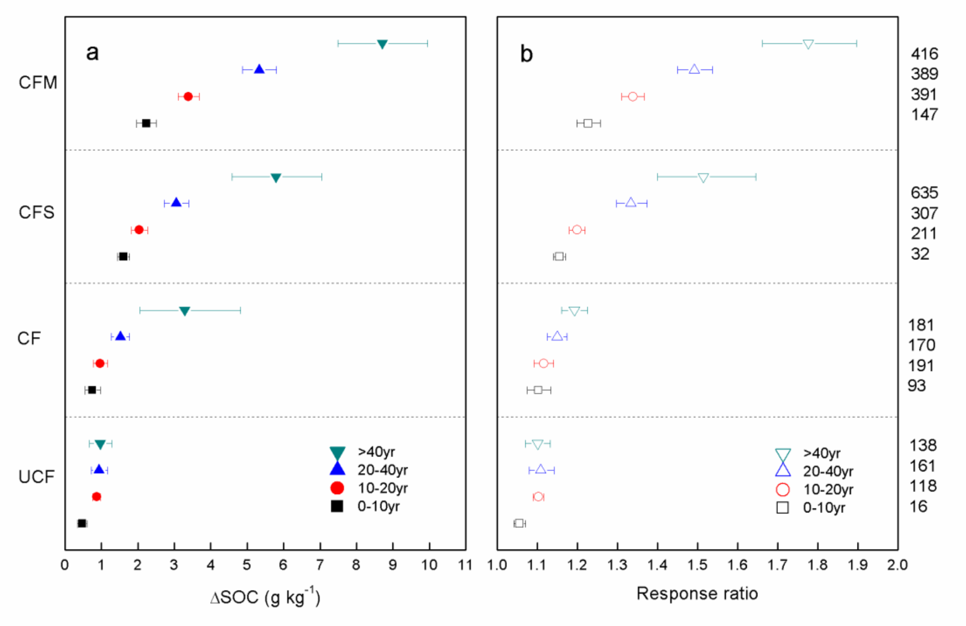


**Figure S5** Experimental duration effects on mean difference in SOC (g $kg^{-1}$) (A) and the relative change (B) comparing with CK.

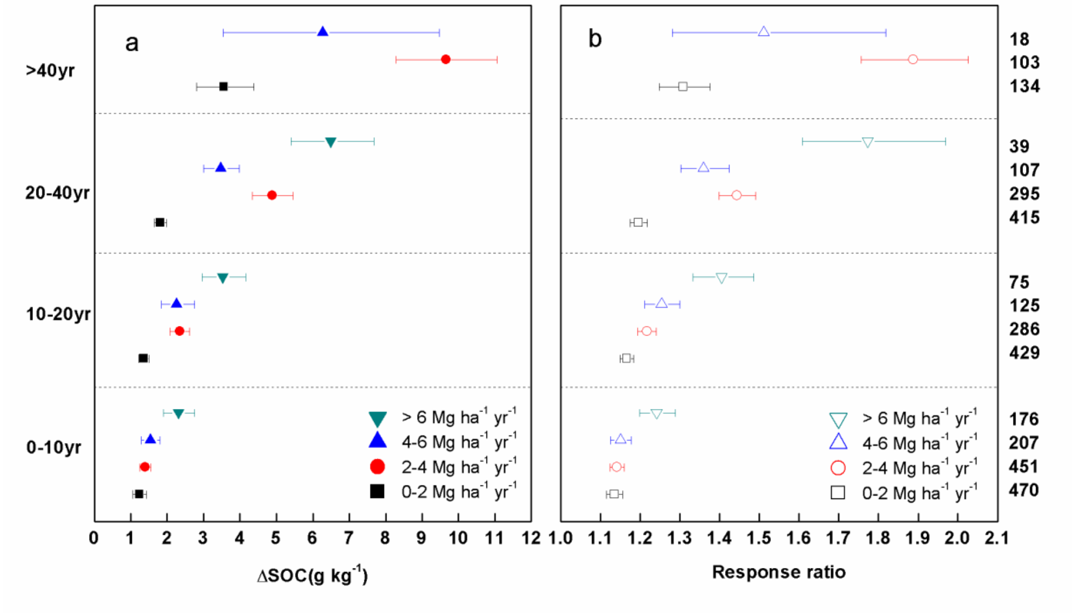


**Figure S6** C input effects on mean difference in SOC (g kg$^{-1}$) (A) and the relative change (B) comparing with CK.

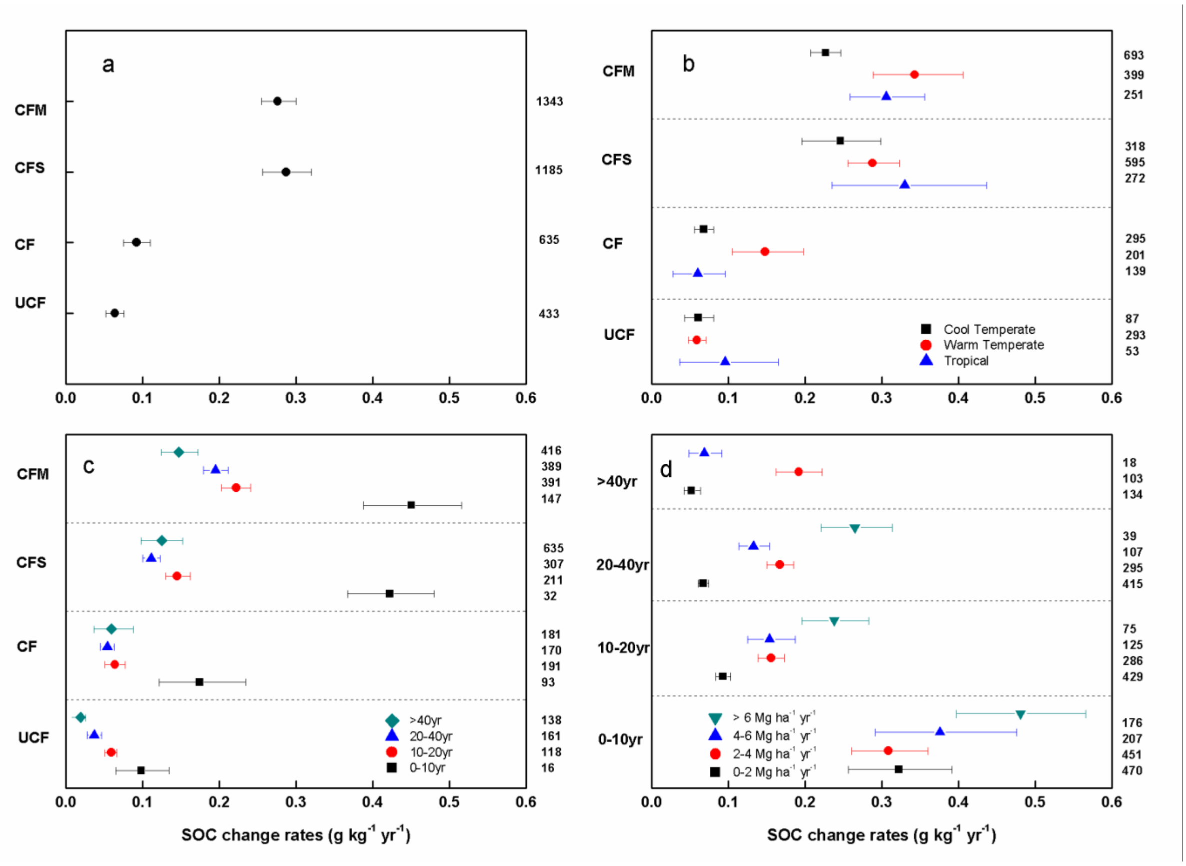


**Figure S7** Effects of main factors on SOC change rates.

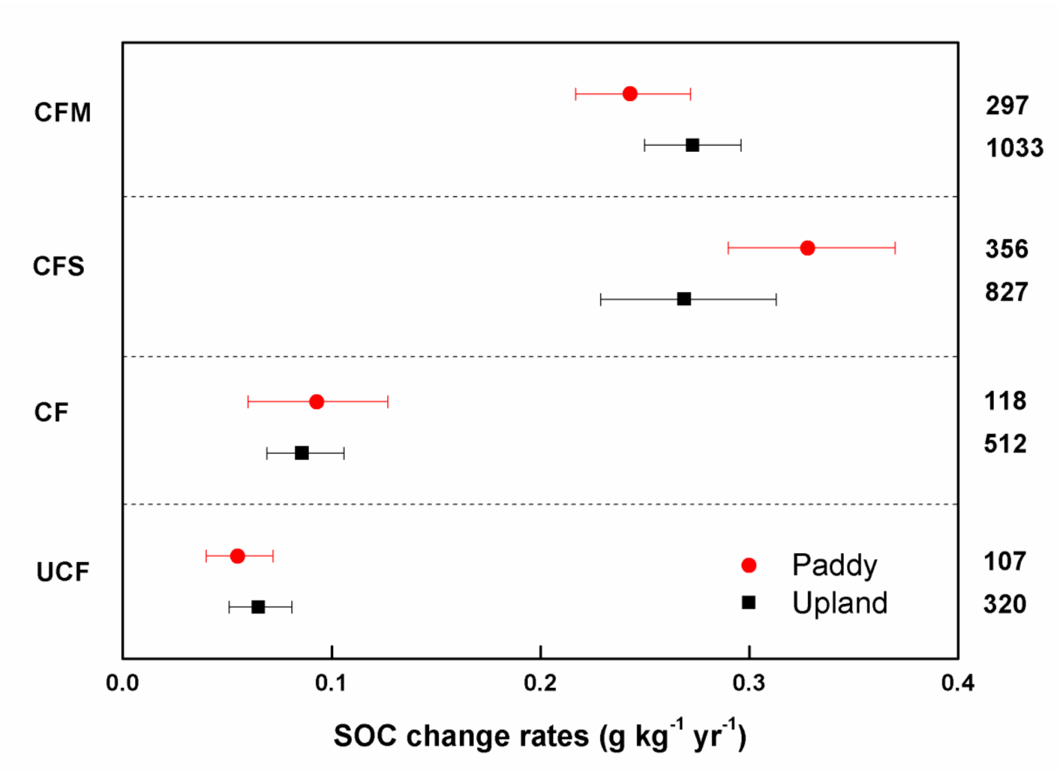


**Figure S8** Effects of upland and paddy on SOC change rates.

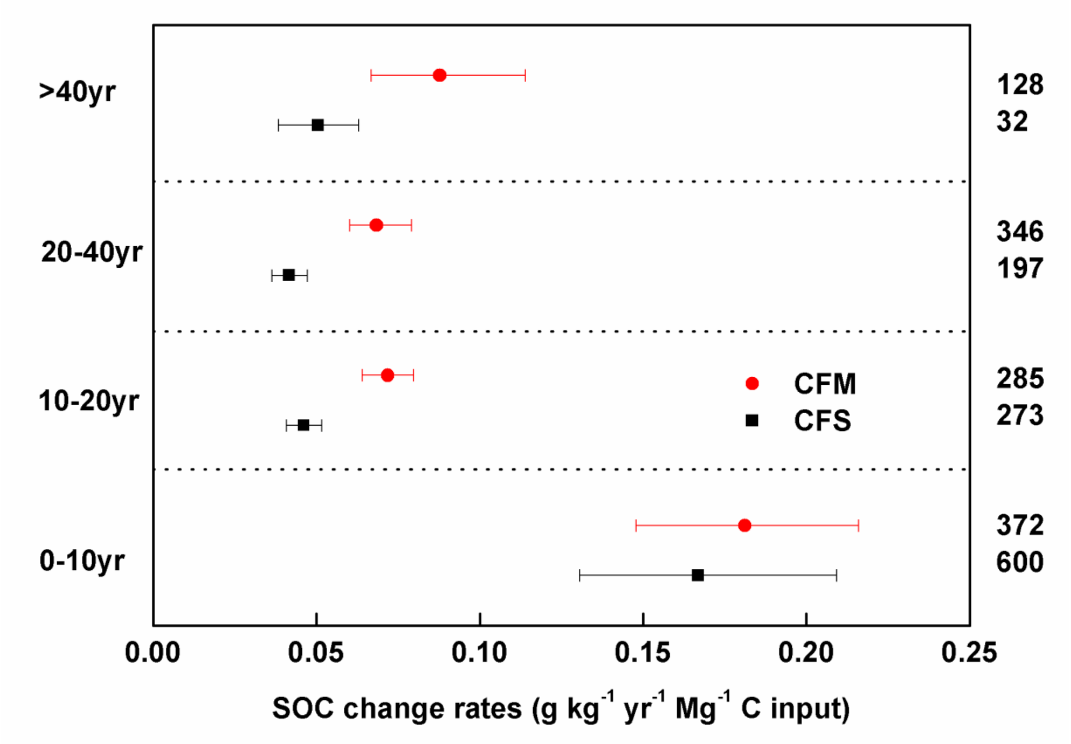


**Figure S9** C sequestration efficiency for manure and straw under different durations.

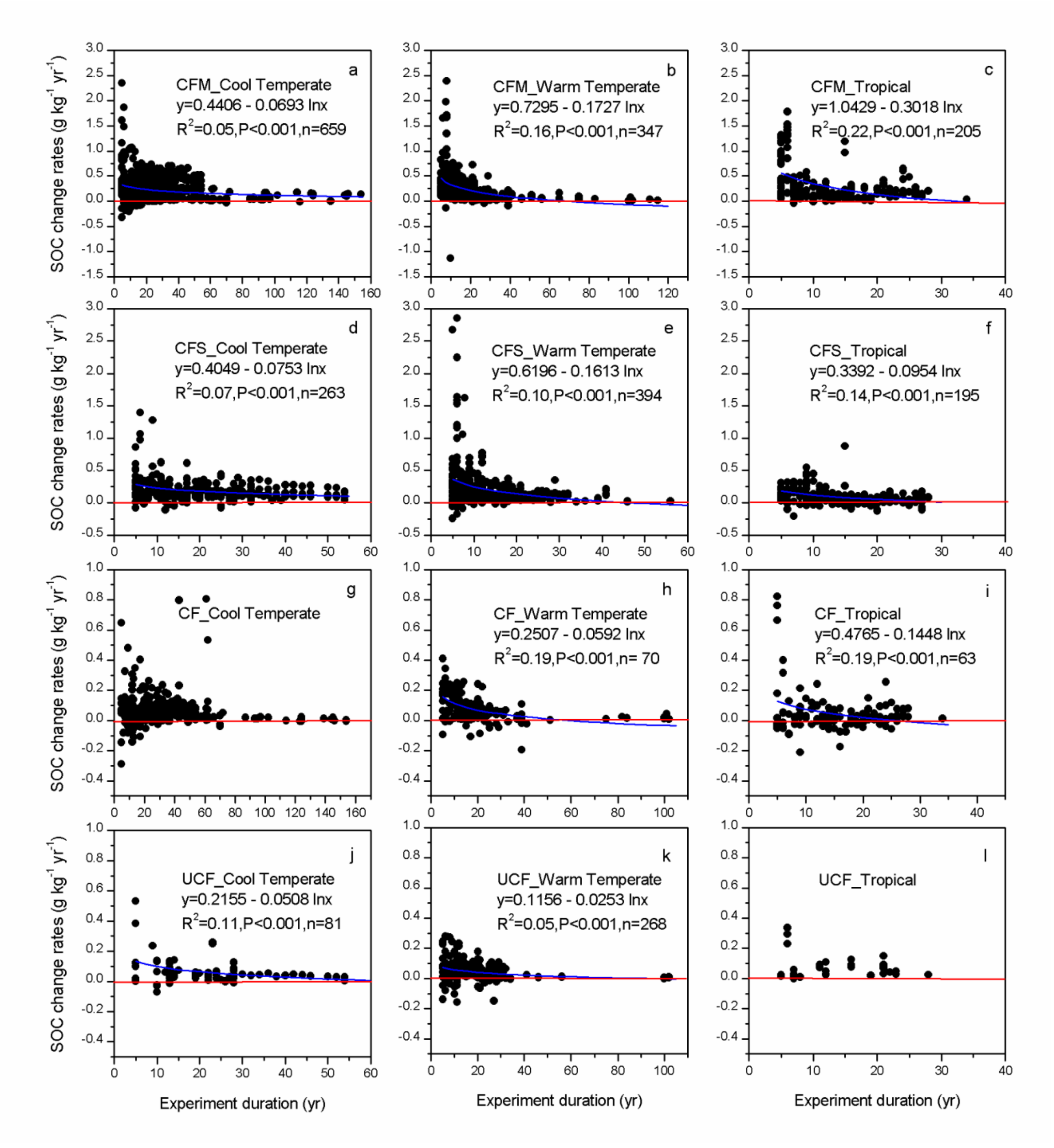


**Figure S10** Relationships between the relative SOC change rates and duration years using the multiple year observations. Experiments with over 5 years were used to reduce the instabilities at the beginning of the experiment.

# 3. The calculation of C sequestration durations under different treatments

A natural log form was used to fit the scattered points as the equation below:

$$RCR = A - B*\ln(x)$$

where x and RCR represent experiment durations and relative SOC change rates, respectively, and A and B are regressed constants. When RCR equals to zero or a lower limit (set to 0.05 g $kg^{-1}$ $yr^{-1}$) that is difficult to detect in field experiments, the calculated x is considered as C sequestration duration.

**Table S2** The C sequestration durations under different treatments. Experiments with over 5 years were used to reduce the instabilities at the beginning of the experiment, and "-" sign denotes no formula could be obtained through regression.

| Fertilization | The last year observations | | Multiple year observations | |
|---|---|---|---|---|
| | Lower | Upper | Lower | Upper |
| CFM_Cool | 72 | 117 | 275 | 564 |
| CFM_Warm | 50 | 65 | 51 | 68 |
| CFM_Tropical | 26 | 30 | 27 | 32 |
| CFS_Cool | 46 | 73 | 110 | 212 |
| CFS_Warm | 37 | 48 | 34 | 47 |
| CFS_Tropical | 28 | 47 | 21 | 35 |
| CF_Cool | - | - | - | - |
| CF_Warm | 30 | 69 | 27 | 60 |
| CF_Tropical | 19 | 27 | 14 | 26 |
| UCF_Cool | - | - | 26 | 70 |
| UCF_Warm | 18 | 58 | 13 | 96 |
| UCF_Tropical | - | - | - | - |

# 4. Initial SOC and N addition effects on SOC changes , differences among groups, publication bias and other analyses.

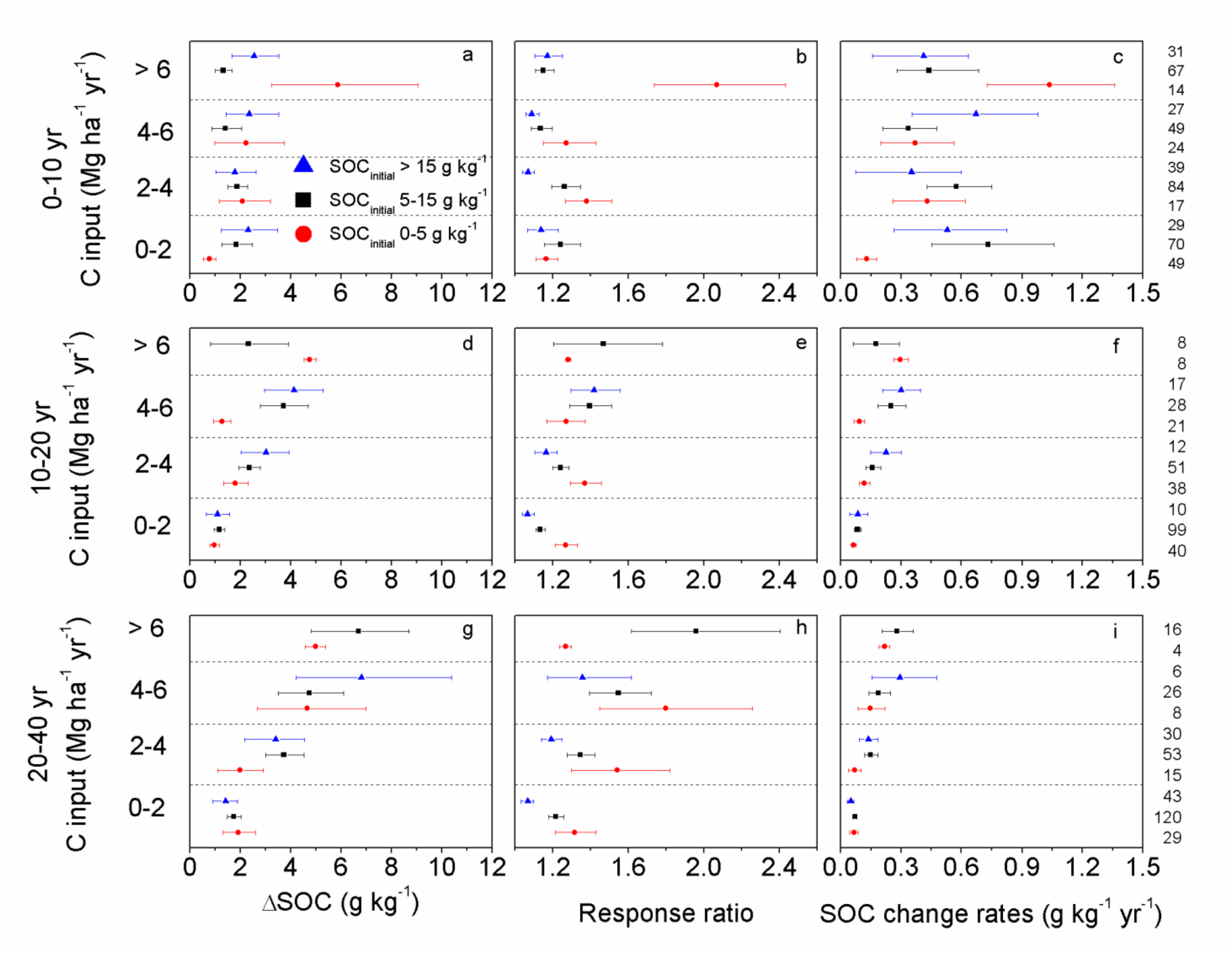


**Figure S11** Initial SOC effects on SOC changes.

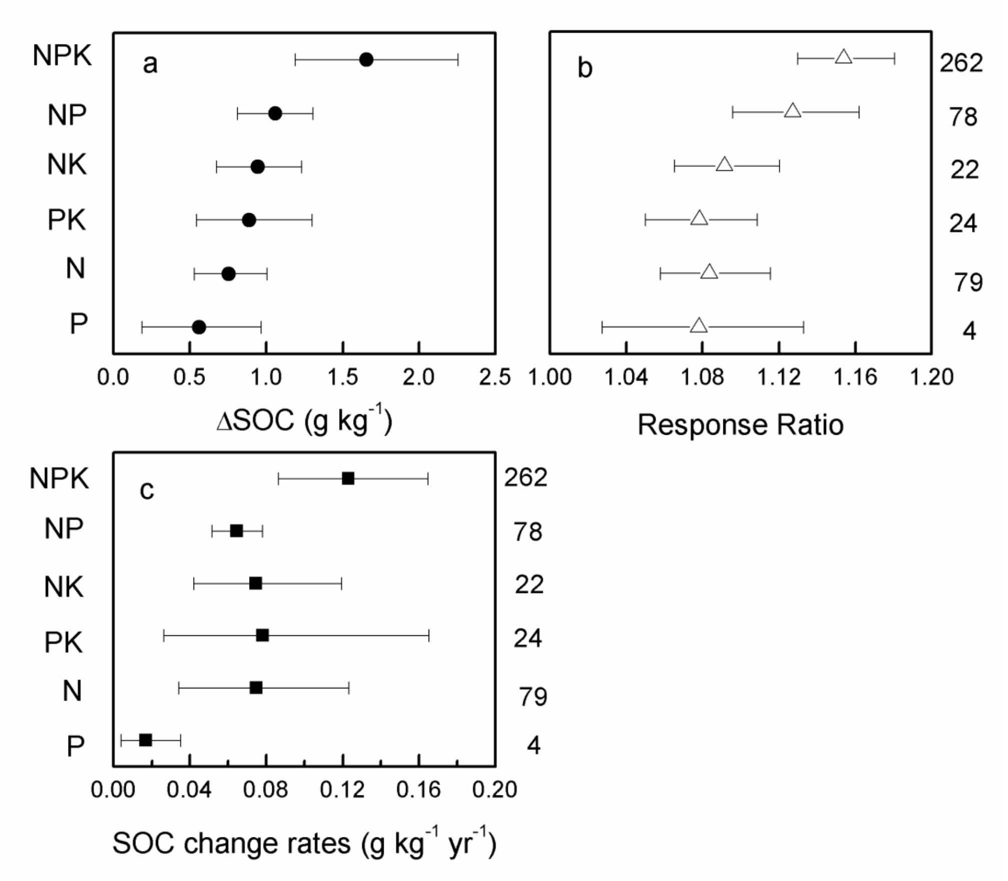


**Figure S12** N addition effects on SOC changes.

**Table S3** Effects of fertilization on between-group heterogeneity (Qb) in relation to the soil organic carbon changes.

| Group | Categorical variable | ΔSOC | | RR | | RCR | |
|---|---|---|---|---|---|---|---|
| | | Qb | P-value | Qb | P-value | Qb | P-value |
| Total | Fertilizer | 1423.50 | **0.000** | 11.07 | **0.000** | 19.12 | **0.000** |
| CFM | Climate zone | 4.93 | 0.081 | 1.28 | **0.000** | 5.60 | **0.000** |
| CFS | | 2.45 | 0.294 | 0.52 | **0.001** | 2.48 | 0.053 |
| CF | | 5.47 | **0.048** | 0.19 | 0.057 | 1.20 | **0.005** |
| UCF | | 3.70 | 0.159 | 0.00 | 0.846 | 0.04 | 0.444 |
| CFM | Experimental duration | 50.73 | **0.000** | 1.88 | **0.000** | 25.95 | **0.000** |
| CFS | | 31.92 | **0.000** | 1.18 | **0.000** | 17.87 | **0.000** |
| CF | | 22.44 | **0.000** | 0.01 | 0.986 | 2.77 | **0.000** |
| UCF | | 9.06 | **0.035** | 0.11 | 0.061 | 0.26 | **0.016** |
| 0-10yr | C input | 6.27 | 0.099 | 0.28 | 0.143 | 0.82 | 0.707 |
| 10-20yr | | 69.92 | **0.000** | 1.34 | **0.000** | 1.23 | **0.000** |
| 20-30yr | | 111.77 | **0.000** | 3.90 | **0.000** | 1.22 | **0.000** |
| 30-40yr | | 31.87 | **0.000** | 1.72 | **0.000** | 0.11 | **0.000** |
| >40yr | | 12.14 | **0.004** | 1.82 | **0.001** | 0.12 | **0.001** |
| 0-10yr | Manure and straw efficiency | - | - | - | - | 6.42 | **0.001** |
| 10-20yr | | - | - | - | - | 0.08 | **0.001** |
| 20-40yr | | - | - | - | - | 0.04 | **0.000** |
| >40yr | | - | - | - | - | 0.00 | 0.797 |

**Table S4** The publication bias of major factors on SOC changes. Boldface for the Kendall's tau rank and Spearman rank indicate significant publication bias at $p < 0.05$.

| Group | Subgroup | n | Kendall's tau rank | Spearman rank |
|---|---|---|---|---|
| Fertilization | | | | |
| UCF | | 207 | 0.135 | 0.146 |
| CF | | 262 | 0.649 | 0.309 |
| CFS | | 620 | 0.650 | 0.367 |
| CFM | | 652 | **0.000** | **0.007** |
| | Climate zone | | | |
| UCF | Cool Temperate | 50 | 0.291 | 0.246 |
| UCF | Warm Temperate | 119 | 0.641 | 0.541 |
| UCF | Tropical | 38 | 0.050 | 0.079 |
| CF | Cool Temperate | 97 | 0.390 | 0.057 |
| CF | Warm Temperate | 89 | 0.059 | **0.045** |
| CF | Tropical | 76 | 0.976 | 0.653 |
| CFS | Cool Temperate | 182 | 0.951 | 0.843 |

| | | | | |
|---|---|---|---|---|
| CFS | Warm Temperate | 324 | 0.268 | 0.543 |
| CFS | Tropical | 114 | 0.947 | 0.884 |
| CFM | Cool Temperate | 251 | **0.003** | **0.007** |
| CFM | Warm Temperate | 209 | 0.440 | 0.589 |
| CFM | Tropical | 192 | **0.023** | 0.082 |
| | Experiment duration | | | |
| UCF | 0-10yr | 44 | **0.048** | 0.082 |
| UCF | 10-20yr | 83 | 0.900 | 0.975 |
| UCF | 20-40yr | 73 | 0.552 | 0.626 |
| UCF | >40yr | 7 | 0.881 | 0.819 |
| CF | 0-10yr | 76 | 0.196 | 0.505 |
| CF | 10-20yr | 60 | 0.293 | 0.258 |
| CF | 20-40yr | 83 | 0.078 | **0.043** |
| CF | >40yr | 43 | 0.917 | 0.726 |
| CFS | 0-10yr | 395 | 0.104 | 0.306 |
| CFS | 10-20yr | 134 | **0.036** | **0.048** |
| CFS | 20-40yr | 77 | 0.286 | 0.233 |
| CFS | >40yr | 14 | 0.139 | 0.409 |
| CFM | 0-10yr | 205 | **0.000** | **0.001** |
| CFM | 10-20yr | 182 | 0.777 | 0.997 |
| CFM | 20-40yr | 200 | 0.756 | 0.352 |
| CFM | >40yr | 65 | **0.011** | **0.038** |
| Experiment duration | C input | | | |
| 0-10yr | 0-2 Mg $ha^{-1}$ $yr^{-1}$ | 215 | **0.000** | **0.001** |
| 0-10yr | 2-4 Mg $ha^{-1}$ $yr^{-1}$ | 215 | **0.003** | 0.086 |
| 0-10yr | 4-6 Mg $ha^{-1}$ $yr^{-1}$ | 115 | **0.000** | **0.000** |
| 0-10yr | >6 Mg $ha^{-1}$ $yr^{-1}$ | 132 | **0.001** | **0.011** |
| 10-20yr | 0-2 Mg $ha^{-1}$ $yr^{-1}$ | 183 | 0.627 | 0.316 |
| 10-20yr | 2-4 Mg $ha^{-1}$ $yr^{-1}$ | 118 | 0.190 | 0.167 |
| 10-20yr | 4-6 Mg $ha^{-1}$ $yr^{-1}$ | 59 | 0.713 | 0.811 |
| 10-20yr | >6 Mg $ha^{-1}$ $yr^{-1}$ | 35 | 0.955 | 0.607 |
| 20-40yr | 0-2 Mg $ha^{-1}$ $yr^{-1}$ | 214 | 0.380 | 0.162 |
| 20-40yr | 2-4 Mg $ha^{-1}$ $yr^{-1}$ | 119 | 0.366 | 0.859 |
| 20-40yr | 4-6 Mg $ha^{-1}$ $yr^{-1}$ | 47 | 0.289 | 0.459 |
| 20-40yr | >6 Mg $ha^{-1}$ $yr^{-1}$ | 26 | 0.496 | 0.584 |
| >40yr | 0-2 Mg $ha^{-1}$ $yr^{-1}$ | 48 | 0.001 | 0.001 |
| >40yr | 2-4 Mg $ha^{-1}$ $yr^{-1}$ | 36 | 0.414 | 0.750 |
| >40yr | 4-6 Mg $ha^{-1}$ $yr^{-1}$ | 13 | 0.393 | 0.459 |

**Table S5** Comparisions of the 95% CI of the last year results and multiple year results. Boldface indicates the 95% CI for the last year results were smaller than the multiple year results.

| Group | 95% CI differences (Last year - Multiple year) | | | 95% CI ranges for the last year results | | | 95% CI ranges for the multiple year results | | |
|---|---|---|---|---|---|---|---|---|---|
| | ΔSOC (g $kg^{-1}$) | RR | RCR (g $kg^{-1}$ $yr^{-1}$) | ΔSOC (g $kg^{-1}$) | RR | RCR (g $kg^{-1}$ $yr^{-1}$) | ΔSOC (g $kg^{-1}$) | RR | RCR (g $kg^{-1}$ $yr^{-1}$) |
| CFM | 0.094 | 0.014 | 0.035 | 0.579 | 0.058 | 0.080 | 0.485 | 0.044 | 0.045 |
| CFS | 0.110 | 0.009 | 0.036 | 0.373 | 0.035 | 0.100 | 0.263 | 0.026 | 0.064 |
| CF | 0.567 | 0.023 | 0.043 | 1.043 | 0.051 | 0.078 | 0.476 | 0.028 | 0.035 |
| UCF | 0.117 | 0.013 | 0.018 | 0.287 | 0.035 | 0.042 | 0.170 | 0.022 | 0.024 |
| CFM_Cool | 0.258 | 0.024 | 0.032 | 1.101 | 0.093 | 0.072 | 0.843 | 0.069 | 0.040 |
| CFM_Warm | 0.348 | 0.037 | 0.093 | 0.909 | 0.103 | 0.210 | 0.561 | 0.066 | 0.117 |
| CFM_Tropical | 0.145 | 0.021 | 0.007 | 0.829 | 0.106 | 0.104 | 0.684 | 0.085 | 0.097 |
| CFS_Cool | 0.074 | 0.006 | 0.077 | 0.695 | 0.066 | 0.180 | 0.621 | 0.060 | 0.103 |
| CFS_Warm | 0.180 | 0.012 | 0.046 | 0.513 | 0.041 | 0.113 | 0.333 | 0.029 | 0.067 |
| CFS_Tropical | 0.425 | 0.044 | 0.146 | 0.877 | 0.103 | 0.348 | 0.452 | 0.059 | 0.202 |
| CF_Cool | 1.758 | 0.026 | 0.028 | 2.705 | 0.063 | 0.053 | 0.947 | 0.036 | 0.025 |
| CF_Warm | 0.422 | 0.041 | 0.105 | 0.826 | 0.085 | 0.198 | 0.404 | 0.044 | 0.093 |
| CF_Tropical | 0.190 | 0.043 | 0.045 | 0.598 | 0.120 | 0.113 | 0.408 | 0.077 | 0.068 |
| UCF_Cool | 0.279 | 0.042 | 0.008 | 0.750 | 0.104 | 0.046 | 0.471 | 0.062 | 0.038 |
| UCF_Warm | 0.134 | 0.015 | 0.024 | 0.314 | 0.038 | 0.047 | 0.180 | 0.023 | 0.023 |
| UCF_Tropical | 0.115 | **-0.001** | 0.024 | 0.667 | 0.063 | 0.152 | 0.552 | 0.064 | 0.128 |
| CFM_0-10yr | 0.331 | 0.047 | 0.097 | 0.869 | 0.104 | 0.225 | 0.538 | 0.058 | 0.128 |
| CFM_10-20yr | 0.165 | 0.017 | 0.012 | 0.742 | 0.073 | 0.050 | 0.577 | 0.056 | 0.038 |

| | | | | | | | | | |
|---|---|---|---|---|---|---|---|---|---|
| CFM_20-40yr | **-0.001** | 0.021 | 0.006 | 0.925 | 0.108 | 0.038 | 0.926 | 0.087 | 0.032 |
| CFM_>40yr | 0.846 | 0.052 | **-0.003** | 3.306 | 0.287 | 0.045 | 2.460 | 0.235 | 0.048 |
| CFS_0-10yr | 0.131 | 0.010 | 0.040 | 0.452 | 0.041 | 0.153 | 0.321 | 0.030 | 0.113 |
| CFS_10-20yr | 0.331 | 0.026 | 0.028 | 0.776 | 0.065 | 0.060 | 0.445 | 0.040 | 0.032 |
| CFS_20-40yr | 0.367 | 0.054 | 0.014 | 1.048 | 0.130 | 0.037 | 0.681 | 0.076 | 0.023 |
| CFS_>40yr | 0.729 | 0.024 | 0.018 | 3.194 | 0.270 | 0.072 | 2.465 | 0.246 | 0.054 |
| CF_0-10yr | 0.438 | 0.069 | 0.138 | 0.847 | 0.128 | 0.251 | 0.409 | 0.059 | 0.113 |
| CF_10-20yr | 0.168 | 0.035 | 0.014 | 0.565 | 0.083 | 0.040 | 0.397 | 0.048 | 0.026 |
| CF_20-40yr | 0.276 | 0.030 | 0.009 | 0.774 | 0.079 | 0.027 | 0.498 | 0.049 | 0.018 |
| CF_>40yr | 3.137 | 0.021 | 0.054 | 5.904 | 0.086 | 0.106 | 2.767 | 0.065 | 0.052 |
| UCF_0-10yr | 0.335 | 0.025 | 0.104 | 0.605 | 0.055 | 0.173 | 0.270 | 0.029 | 0.069 |
| UCF_10-20yr | 0.101 | 0.008 | 0.008 | 0.309 | 0.034 | 0.023 | 0.208 | 0.026 | 0.015 |
| UCF_20-40yr | 0.161 | 0.023 | 0.006 | 0.606 | 0.084 | 0.024 | 0.445 | 0.061 | 0.018 |
| UCF_>40yr | **-0.016** | 0.000 | **-0.001** | 0.600 | 0.063 | 0.013 | 0.616 | 0.063 | 0.014 |
| 0-10yr_0-2Mg $ha^{-1}$ $yr^{-1}$ | 0.363 | 0.038 | 2.239 | 0.726 | 0.079 | 2.374 | 0.363 | 0.040 | 0.135 |
| 0-10yr_2-4Mg $ha^{-1}$ $yr^{-1}$ | 0.178 | 0.029 | 1.661 | 0.484 | 0.065 | 1.760 | 0.306 | 0.037 | 0.099 |
| 0-10yr_4-6Mg $ha^{-1}$ $yr^{-1}$ | 0.290 | 0.027 | 1.995 | 0.812 | 0.077 | 2.179 | 0.522 | 0.051 | 0.184 |
| 0-10yr_>6Mg $ha^{-1}$ $yr^{-1}$ | 0.138 | 0.015 | 2.553 | 1.000 | 0.104 | 2.722 | 0.862 | 0.089 | 0.169 |
| 10-20yr_0-2Mg $ha^{-1}$ $yr^{-1}$ | 0.110 | 0.014 | 0.215 | 0.391 | 0.048 | 0.234 | 0.281 | 0.034 | 0.019 |
| 10-20yr_2-4Mg $ha^{-1}$ $yr^{-1}$ | 0.183 | 0.023 | 0.467 | 0.716 | 0.069 | 0.501 | 0.533 | 0.046 | 0.034 |
| 10-20yr_4-6Mg $ha^{-1}$ $yr^{-1}$ | 0.732 | 0.059 | 1.101 | 1.640 | 0.147 | 1.163 | 0.908 | 0.088 | 0.062 |
| 10-20yr_>6Mg $ha^{-1}$ $yr^{-1}$ | 0.188 | **-0.011** | 1.064 | 1.393 | 0.143 | 1.151 | 1.205 | 0.154 | 0.087 |
| 20-40yr_0-2Mg $ha^{-1}$ $yr^{-1}$ | 0.101 | 0.018 | 0.181 | 0.430 | 0.061 | 0.194 | 0.329 | 0.043 | 0.013 |
| 20-40yr_2-4Mg $ha^{-1}$ $yr^{-1}$ | **-0.050** | 0.018 | 0.440 | 1.058 | 0.112 | 0.475 | 1.108 | 0.094 | 0.035 |
| 20-40yr_4-6Mg $ha^{-1}$ $yr^{-1}$ | 0.519 | 0.059 | 0.785 | 1.500 | 0.180 | 0.825 | 0.981 | 0.121 | 0.040 |

| | | | | | | | | | |
|---|---|---|---|---|---|---|---|---|---|
| 20-40yr_>6Mg $ha^{-1}$ $yr^{-1}$ | 0.139 | 0.030 | 0.226 | 2.410 | 0.390 | 0.319 | 2.271 | 0.360 | 0.093 |
| >40yr_0-2Mg $ha^{-1}$ $yr^{-1}$ | 0.799 | 0.055 | 0.744 | 2.377 | 0.182 | 0.766 | 1.578 | 0.127 | 0.022 |
| >40yr_2-4Mg $ha^{-1}$ $yr^{-1}$ | 1.363 | 0.125 | 0.669 | 4.137 | 0.395 | 0.729 | 2.774 | 0.269 | 0.060 |
| >40yr_4-6Mg $ha^{-1}$ $yr^{-1}$ | 1.407 | 0.065 | 1.591 | 7.333 | 0.601 | 1.634 | 5.926 | 0.536 | 0.043 |

**Table S6** Comparisions of the unweighted and weighted RR and ΔSOC.

| Group | n | Method | RR | | | RR differences (%) | | | ΔSOC (g kg$^{-1}$) | | | ΔSOC differences (%) | | |
|---|---|---|---|---|---|---|---|---|---|---|---|---|---|---|
| | | | Mean | 95%CI Lower | 95%CI Upper | Mean | 95%CI Lower | 95%CI Upper | Mean | 95%CI Lower | 95%CI Upper | Mean | 95%CI Lower | 95%CI Upper |
| CFM | 652 | Unweighted | 1.36 | 1.33 | 1.39 | 0.18 | 0.17 | 0.24 | 3.47 | 3.18 | 3.76 | -0.81 | -0.53 | -1.12 |
| | | Weighted | 1.37 | 1.34 | 1.40 | | | | 3.44 | 3.17 | 3.72 | | | |
| CFS | 620 | Unweighted | 1.20 | 1.18 | 1.21 | 0.08 | 0.08 | 0.07 | 2.06 | 1.88 | 2.25 | -0.97 | -1.23 | -0.85 |
| | | Weighted | 1.20 | 1.18 | 1.21 | | | | 2.04 | 1.85 | 2.23 | | | |
| CF | 262 | Unweighted | 1.15 | 1.13 | 1.18 | 0.14 | 0.12 | 0.15 | 1.66 | 1.19 | 2.24 | -7.67 | -2.45 | -10.95 |
| | | Weighted | 1.16 | 1.13 | 1.18 | | | | 1.53 | 1.16 | 1.99 | | | |
| UCF | 207 | Unweighted | 1.10 | 1.08 | 1.12 | 0.04 | 0.05 | 0.11 | 0.89 | 0.75 | 1.04 | -0.79 | -0.53 | -0.97 |
| | | Weighted | 1.10 | 1.08 | 1.12 | | | | 0.88 | 0.74 | 1.03 | | | |
| CFM_Cool | 251 | Unweighted | 1.30 | 1.25 | 1.34 | 0.19 | 0.23 | 0.17 | 3.54 | 3.01 | 4.10 | -0.85 | -0.43 | -0.73 |
| | | Weighted | 1.30 | 1.26 | 1.35 | | | | 3.51 | 2.99 | 4.07 | | | |
| CFM_Warm | 209 | Unweighted | 1.40 | 1.35 | 1.45 | 2.09 | 1.91 | 2.34 | 3.85 | 3.40 | 4.31 | 0.26 | 0.77 | 0.07 |
| | | Weighted | 1.43 | 1.38 | 1.49 | | | | 3.86 | 3.42 | 4.31 | | | |
| CFM_Tropical | 192 | Unweighted | 1.41 | 1.36 | 1.47 | 1.31 | 0.91 | 1.62 | 2.97 | 2.57 | 3.41 | -1.92 | -1.91 | -2.58 |
| | | Weighted | 1.43 | 1.38 | 1.49 | | | | 2.91 | 2.52 | 3.32 | | | |
| CFS_Cool | 182 | Unweighted | 1.19 | 1.16 | 1.22 | 0.56 | 0.35 | 0.70 | 2.05 | 1.72 | 2.41 | -0.68 | -0.23 | -0.87 |
| | | Weighted | 1.19 | 1.16 | 1.23 | | | | 2.04 | 1.72 | 2.39 | | | |
| CFS_Warm | 324 | Unweighted | 1.17 | 1.15 | 1.20 | 0.20 | 0.15 | 0.23 | 2.17 | 1.91 | 2.43 | -0.88 | -0.84 | -0.95 |
| | | Weighted | 1.18 | 1.16 | 1.20 | | | | 2.15 | 1.90 | 2.41 | | | |
| CFS_Tropical | 114 | Unweighted | 1.27 | 1.22 | 1.32 | 0.22 | 0.09 | 0.08 | 1.77 | 1.34 | 2.24 | -1.70 | -0.89 | -2.19 |
| | | Weighted | 1.27 | 1.22 | 1.32 | | | | 1.74 | 1.33 | 2.19 | | | |

| | | | | | | | | | | | | | | |
|---|---|---|---|---|---|---|---|---|---|---|---|---|---|---|
| CF_Cool | 97 | Unweighted | 1.12 | 1.09 | 1.15 | 0.21 | 0.09 | 0.42 | 2.49 | 1.31 | 4.06 | -13.99 | -5.58 | -17.92 |
| | | Weighted | 1.12 | 1.09 | 1.16 | | | | 2.14 | 1.24 | 3.33 | | | |
| CF_Warm | 89 | Unweighted | 1.16 | 1.12 | 1.20 | 1.23 | 1.03 | 1.43 | 1.37 | 0.95 | 1.79 | 1.32 | 5.60 | -0.11 |
| | | Weighted | 1.17 | 1.13 | 1.22 | | | | 1.38 | 1.00 | 1.79 | | | |
| CF_Tropical | 76 | Unweighted | 1.19 | 1.13 | 1.25 | 2.14 | 1.76 | 2.58 | 0.94 | 0.65 | 1.25 | 0.11 | 0.92 | 0.56 |
| | | Weighted | 1.22 | 1.15 | 1.29 | | | | 0.94 | 0.66 | 1.26 | | | |
| UCF_Cool | 50 | Unweighted | 1.11 | 1.06 | 1.17 | 0.76 | 0.22 | 1.41 | 1.14 | 0.78 | 1.53 | -0.26 | -0.38 | -0.20 |
| | | Weighted | 1.12 | 1.07 | 1.18 | | | | 1.14 | 0.78 | 1.53 | | | |
| UCF_Warm | 119 | Unweighted | 1.10 | 1.08 | 1.12 | 0.29 | 0.27 | 0.30 | 0.83 | 0.67 | 0.98 | -0.24 | 0.30 | -0.20 |
| | | Weighted | 1.10 | 1.08 | 1.12 | | | | 0.83 | 0.67 | 0.98 | | | |
| UCF_Tropical | 38 | Unweighted | 1.09 | 1.06 | 1.12 | -0.38 | -0.48 | -0.21 | 0.76 | 0.45 | 1.12 | -2.76 | -1.56 | -2.60 |
| | | Weighted | 1.09 | 1.06 | 1.12 | | | | 0.74 | 0.44 | 1.09 | | | |
| CFM_0-10yr | 205 | Unweighted | 1.30 | 1.25 | 1.36 | 0.32 | 0.32 | 0.49 | 2.70 | 2.28 | 3.14 | -0.56 | -0.74 | -0.73 |
| | | Weighted | 1.31 | 1.26 | 1.37 | | | | 2.68 | 2.27 | 3.12 | | | |
| CFM_10-20yr | 182 | Unweighted | 1.31 | 1.28 | 1.35 | 0.14 | 0.16 | 0.19 | 2.78 | 2.42 | 3.16 | -1.22 | -0.78 | -1.52 |
| | | Weighted | 1.31 | 1.28 | 1.35 | | | | 2.75 | 2.40 | 3.11 | | | |
| CFM_20-40yr | 200 | Unweighted | 1.42 | 1.36 | 1.47 | 0.28 | 0.29 | 0.23 | 4.08 | 3.62 | 4.56 | -0.29 | 0.19 | -0.64 |
| | | Weighted | 1.42 | 1.37 | 1.48 | | | | 4.07 | 3.63 | 4.53 | | | |
| CFM_>40yr | 65 | Unweighted | 1.55 | 1.41 | 1.70 | -0.08 | 0.14 | -0.25 | 5.96 | 4.38 | 7.68 | -1.28 | -0.05 | -1.28 |
| | | Weighted | 1.55 | 1.42 | 1.70 | | | | 5.88 | 4.37 | 7.58 | | | |
| CFS_0-10yr | 395 | Unweighted | 1.16 | 1.14 | 1.18 | 0.06 | 0.08 | 0.08 | 1.75 | 1.54 | 1.99 | -1.14 | -1.56 | -1.26 |
| | | Weighted | 1.16 | 1.14 | 1.18 | | | | 1.73 | 1.51 | 1.96 | | | |
| CFS_10-20yr | 134 | Unweighted | 1.23 | 1.20 | 1.26 | 0.04 | 0.00 | 0.07 | 2.32 | 1.95 | 2.72 | -1.08 | -0.67 | -1.14 |
| | | Weighted | 1.23 | 1.20 | 1.26 | | | | 2.30 | 1.94 | 2.69 | | | |
| CFS_20-40yr | 77 | Unweighted | 1.29 | 1.23 | 1.36 | 0.31 | 0.41 | 0.49 | 2.65 | 2.14 | 3.20 | -0.38 | -0.56 | -0.81 |

| | | | | | | | | | | | | | | |
|---|---|---|---|---|---|---|---|---|---|---|---|---|---|---|
| | | Weighted | 1.30 | 1.24 | 1.37 | | | | 2.64 | 2.13 | 3.17 | | | |
| CFS_>40yr | 14 | Unweighted | 1.38 | 1.25 | 1.53 | 0.01 | 0.13 | -0.01 | 4.83 | 3.26 | 6.41 | -0.99 | -0.15 | -0.19 |
| | | Weighted | 1.38 | 1.25 | 1.53 | | | | 4.79 | 3.25 | 6.40 | | | |
| CF_0-10yr | 76 | Unweighted | 1.15 | 1.09 | 1.22 | 0.21 | 0.11 | 0.22 | 1.00 | 0.59 | 1.45 | 0.60 | 1.18 | 0.90 |
| | | Weighted | 1.15 | 1.09 | 1.22 | | | | 1.01 | 0.60 | 1.46 | | | |
| CF_10-20yr | 60 | Unweighted | 1.15 | 1.11 | 1.20 | 0.14 | 0.16 | 0.12 | 0.97 | 0.69 | 1.27 | -0.10 | 1.30 | -1.50 |
| | | Weighted | 1.16 | 1.12 | 1.20 | | | | 0.97 | 0.70 | 1.25 | | | |
| CF_20-40yr | 83 | Unweighted | 1.15 | 1.11 | 1.19 | 0.27 | 0.22 | 0.28 | 1.33 | 0.93 | 1.71 | 0.98 | 5.48 | -0.82 |
| | | Weighted | 1.16 | 1.12 | 1.20 | | | | 1.34 | 0.98 | 1.70 | | | |
| CF_>40yr | 43 | Unweighted | 1.16 | 1.12 | 1.21 | -0.18 | -0.06 | -0.43 | 4.45 | 1.89 | 7.73 | -16.42 | -9.20 | -17.39 |
| | | Weighted | 1.16 | 1.12 | 1.20 | | | | 3.72 | 1.72 | 6.38 | | | |
| UCF_0-10yr | 44 | Unweighted | 1.06 | 1.03 | 1.08 | -0.01 | -0.01 | 0.05 | 0.52 | 0.23 | 0.84 | -3.09 | -0.43 | -3.20 |
| | | Weighted | 1.06 | 1.03 | 1.09 | | | | 0.50 | 0.23 | 0.82 | | | |
| UCF_10-20yr | 83 | Unweighted | 1.10 | 1.09 | 1.12 | 0.02 | 0.04 | 0.03 | 0.92 | 0.77 | 1.08 | -0.54 | -0.26 | -0.28 |
| | | Weighted | 1.10 | 1.09 | 1.12 | | | | 0.91 | 0.77 | 1.07 | | | |
| UCF_20-40yr | 73 | Unweighted | 1.12 | 1.09 | 1.17 | 0.12 | -0.02 | 0.03 | 1.10 | 0.81 | 1.41 | -0.27 | 0.00 | -0.35 |
| | | Weighted | 1.13 | 1.09 | 1.17 | | | | 1.10 | 0.81 | 1.40 | | | |
| UCF_>40yr | 7 | Unweighted | 1.09 | 1.06 | 1.12 | 0.00 | -0.03 | 0.01 | 0.77 | 0.48 | 1.08 | -0.65 | -2.08 | -0.83 |
| | | Weighted | 1.09 | 1.06 | 1.12 | | | | 0.77 | 0.47 | 1.07 | | | |
| 0-10yr_0-2Mg $ha^{-1}$ $yr^{-1}$ | 215 | Unweighted | 1.19 | 1.16 | 1.24 | 0.22 | -0.07 | 0.52 | 1.78 | 1.41 | 2.15 | -1.69 | -0.71 | -2.04 |
| | | Weighted | 1.20 | 1.15 | 1.24 | | | | 1.75 | 1.40 | 2.11 | | | |
| 0-10yr_2-4Mg $ha^{-1}$ $yr^{-1}$ | 215 | Unweighted | 1.17 | 1.14 | 1.21 | 1.77 | 1.16 | 2.46 | 1.59 | 1.36 | 1.83 | -1.01 | -1.03 | -1.04 |
| | | Weighted | 1.20 | 1.16 | 1.24 | | | | 1.57 | 1.34 | 1.81 | | | |
| 0-10yr_4-6Mg $ha^{-1}$ $yr^{-1}$ | 115 | Unweighted | 1.14 | 1.11 | 1.18 | 0.75 | 0.38 | 1.19 | 1.65 | 1.26 | 2.07 | -1.69 | -1.50 | -1.06 |
| | | Weighted | 1.15 | 1.11 | 1.20 | | | | 1.62 | 1.25 | 2.05 | | | |

| | | | | | | | | | | | | | | |
|---|---|---|---|---|---|---|---|---|---|---|---|---|---|---|
| 0-10yr_>6Mg ha$^{-1}$ yr$^{-1}$ | 132 | Unweighted | 1.24 | 1.19 | 1.30 | 0.93 | 0.82 | 1.22 | 2.42 | 1.95 | 2.95 | -0.54 | -1.08 | -0.98 |
| | | Weighted | 1.25 | 1.20 | 1.31 | | | | 2.40 | 1.93 | 2.92 | | | |
| 10-20yr_0-2Mg ha$^{-1}$ yr$^{-1}$ | 183 | Unweighted | 1.17 | 1.15 | 1.20 | 0.74 | 0.61 | 0.98 | 1.24 | 1.06 | 1.44 | -0.97 | -0.19 | -1.04 |
| | | Weighted | 1.18 | 1.16 | 1.21 | | | | 1.23 | 1.05 | 1.43 | | | |
| 10-20yr_2-4Mg ha$^{-1}$ yr$^{-1}$ | 118 | Unweighted | 1.24 | 1.21 | 1.28 | 0.91 | 0.66 | 1.12 | 2.35 | 2.01 | 2.74 | -1.15 | -1.34 | -1.68 |
| | | Weighted | 1.25 | 1.22 | 1.29 | | | | 2.32 | 1.98 | 2.69 | | | |
| 10-20yr_4-6Mg ha$^{-1}$ yr$^{-1}$ | 59 | Unweighted | 1.29 | 1.22 | 1.37 | -0.33 | -0.66 | -0.08 | 2.64 | 1.91 | 3.52 | -1.51 | -1.05 | -2.47 |
| | | Weighted | 1.29 | 1.21 | 1.36 | | | | 2.60 | 1.89 | 3.44 | | | |
| 10-20yr_>6Mg ha$^{-1}$ yr$^{-1}$ | 35 | Unweighted | 1.37 | 1.30 | 1.44 | 1.13 | 0.78 | 1.29 | 3.36 | 2.71 | 4.09 | -1.22 | -1.36 | -1.86 |
| | | Weighted | 1.38 | 1.31 | 1.46 | | | | 3.32 | 2.67 | 4.02 | | | |
| 20-40yr_0-2Mg ha$^{-1}$ yr$^{-1}$ | 213 | Unweighted | 1.18 | 1.15 | 1.21 | 1.48 | 1.39 | 1.75 | 1.63 | 1.41 | 1.84 | -0.31 | -0.07 | -0.43 |
| | | Weighted | 1.20 | 1.17 | 1.23 | | | | 1.62 | 1.41 | 1.83 | | | |
| 20-40yr_2-4Mg ha$^{-1}$ yr$^{-1}$ | 119 | Unweighted | 1.31 | 1.26 | 1.37 | 2.42 | 1.96 | 2.96 | 3.02 | 2.50 | 3.56 | 0.66 | 2.24 | 0.42 |
| | | Weighted | 1.35 | 1.28 | 1.41 | | | | 3.04 | 2.56 | 3.57 | | | |
| 20-40yr_4-6Mg ha$^{-1}$ yr$^{-1}$ | 47 | Unweighted | 1.41 | 1.32 | 1.50 | 2.04 | 1.30 | 3.02 | 4.14 | 3.41 | 4.93 | -0.72 | -0.82 | -0.87 |
| | | Weighted | 1.44 | 1.34 | 1.55 | | | | 4.11 | 3.39 | 4.89 | | | |
| 20-40yr_>6Mg ha$^{-1}$ yr$^{-1}$ | 26 | Unweighted | 1.80 | 1.62 | 2.01 | 0.54 | 0.99 | 0.84 | 6.73 | 5.61 | 7.96 | -0.48 | -0.87 | -0.73 |
| | | Weighted | 1.81 | 1.64 | 2.03 | | | | 6.70 | 5.56 | 7.91 | | | |
| >40yr_0-2Mg ha$^{-1}$ yr$^{-1}$ | 48 | Unweighted | 1.24 | 1.16 | 1.34 | -1.15 | -0.23 | -2.59 | 2.67 | 1.64 | 3.98 | -1.20 | -1.16 | -2.11 |
| | | Weighted | 1.22 | 1.15 | 1.31 | | | | 2.63 | 1.62 | 3.89 | | | |
| >40yr_2-4Mg ha$^{-1}$ yr$^{-1}$ | 36 | Unweighted | 1.66 | 1.48 | 1.88 | 0.72 | 1.42 | -0.03 | 6.93 | 4.96 | 9.13 | -1.05 | -1.21 | -1.66 |
| | | Weighted | 1.68 | 1.50 | 1.88 | | | | 6.86 | 4.90 | 8.98 | | | |
| >40yr_4-6Mg ha$^{-1}$ yr$^{-1}$ | 13 | Unweighted | 1.43 | 1.20 | 1.80 | -0.88 | 1.39 | -2.85 | 5.60 | 2.41 | 9.80 | -0.93 | 0.00 | -2.47 |
| | | Weighted | 1.42 | 1.22 | 1.75 | | | | 5.54 | 2.41 | 9.56 | | | |

# 5. References of the databases

## Data from the Web of Science® (1900-2014):


1. Ailincai, C., Jitareanu, G., Bucur, D. & Mercus, A. Influence of tillage practices and fertilization on crop yields and soil properties in long-term crop rotation (soybean-wheat-maize) experiments. *J Food Agr Environ* **9**, 285-289 (2011).
2. Anderson, T. H. & Domsch, K. H. Ratios of microbial biomass carbon to total organic carbon in arable soils. *Soil Biol Biochem* **21**, 471-479 (1989).
3. Angers, D. A., Chantigny, M. H., MacDonald, J. D., Rochette, P. & Cote, D. Differential retention of carbon, nitrogen and phosphorus in grassland soil profiles with long-term manure application. *Nutr Cycl Agroecosys* **86**, 225-229 (2010).
4. Anken, T. *et al.* Long-term tillage system effects under moist cool conditions in Switzerland. *Soil Till Res* **78**, 171-183 (2004).
5. Anyanzwa, H. *et al.* Effects of conservation tillage, crop residue and cropping systems on changes in soil organic matter and maize–legume production: a case study in Teso District. *Nutr Cycl Agroecosys* **88**, 39-47 (2010).
6. Aulakh, M. S., Khera, T. S., Doran, J. W. & Bronson, K. F. Managing crop residue with green manure, urea, and tillage in a rice–wheat rotation. *Soil Sci Soc Am J* **65**, 820-827 (2001).
7. Bahrani, M. J., Kheradnam, M., Emam, Y., Ghadiri, H. & Assad, M. Effects of tillage methods on wheat yield and yield components in continuous wheat cropping. *Exp Agr* **38**, 389-395 (2002).
8. Balesdent, J., Mariotti, A. & Boisgontier, D. Effect of tillage on soil organic carbon mineralization estimated from 13C abundance in maize fields. *J Soil Sci* **41**, 587-596 (1990).
9. Bayer, C., Lovato, T., Dieckow, J., Zanatta, J. & Mielniczuk, J. A method for estimating coefficients of soil organic matter dynamics based on long-term experiments. *Soil Till Res* **91**, 217-226 (2006).
10. Benbi, D. K. & Senapati, N. Soil aggregation and carbon and nitrogen stabilization in relation to residue and manure application in rice–wheat systems in northwest India. *Nutr Cycl Agroecosys* **87**, 233-247 (2010).
11. Bhattacharyya, P. *et al.* Effects of rice straw and nitrogen fertilization on greenhouse gas emissions and carbon storage in tropical flooded soil planted with rice. *Soil Till Res* **124**, 119-130 (2012).
12. Bhattacharyya, T. *et al.* Evaluating the Century C model using long-term fertilizer trials in the Indo-Gangetic Plains, India. *Agr Ecosyst Environ* **122**, 73-83 (2007).
13. Bhogal, A. *et al.* Effects of recent and accumulated livestock manure carbon additions on soil fertility and quality. *Eur J Soil Sci* **62**, 174-181 (2011).
14. Bi, L. *et al.* Long-term effects of organic amendments on the rice yields for double rice cropping systems in subtropical China. *Agr Ecosyst Environ* **129**, 534-541 (2009).
15. Blaise, D. & Ravindran, C. D. Influence of tillage and residue management on growth and yield of cotton grown on a vertisol over 5 years in a semi-arid region of India. *Soil Till Res* **70**, 163-173 (2003).

16. Bragato, G., Leita, L., Figliolia, A. & De Nobili, M. Effects of sewage sludge pre-treatment on microbial biomass and biovailability of heavy metals. *Soil Till Res* **46**, 129-134 (1998).
17. Bruun, S., Christensen, B. T., Hansen, E. M., Magid, J. & Jensen, L. S. Calibration and validation of the soil organic matter dynamics of the Daisy model with data from the Askov long-term experiments. *Soil Biol Biochem* **35**, 67-76 (2003).
18. Buyanovsky, G. A. & Wagner, G. H. Carbon cycling in cultivated land and its global significance. *Global Change Biol* **4**, 131-141 (1998).
19. Campbell, C. A., McConkey, B. G., Zentner, R. P., Selles, F. & Curtin, D. Tillage and crop rotation effects on soil organic C and N in a coarse-textured Typic Haploboroll in southwestern Saskatchewan. *Soil Till Res* **37**, 3-14 (1996).
20. Campbell, C. A., Selles, F., Lafond, G. P., McConkey, B. & Hahn, D. Effect of crop management on C and N in long-term crop rotations after adopting no-tillage management: comparison of soil sampling strategies. *Can J Soil Sci* **78**, 155-162 (1998).
21. Carlgren, K. & Mattsson, L. Swedish soil fertility experiments. *Acta Agr Scand B-s P* **51**, 49-76 (2001).
22. Leite, L. F. C., Sá Mendonça, E. d., Machado, P. L. O. d. A., Inácio Fernandes Filho, E. d. & Lima Neves, J. C. Simulating trends in soil organic carbon of an Acrisol under no-tillage and disc-plow systems using the Century model. *Geoderma* **120**, 283-295 (2004).
23. Chan, K. Y. *et al.* Soil carbon dynamics under different cropping and pasture management in temperate Australia: Results of three long-term experiments. *Soil Res* **49**, 320-328 (2011).
24. Chen, H. *et al.* Effects of 11 years of conservation tillage on soil organic matter fractions in wheat monoculture in Loess Plateau of China. *Soil Till Res* **106**, 85-94 (2009).
25. Chirinda, N., Olesen, J. E., Porter, J. R. & Schjønning, P. Soil properties, crop production and greenhouse gas emissions from organic and inorganic fertilizer-based arable cropping systems. *Agr Ecosyst Environ* **139**, 584-594 (2010).
26. Chung, H., Ngo, K. J., Plante, A. & Six, J. Evidence for carbon saturation in a highly structured and organic-matter-rich soil. *Soil Sci Soc Am J* **74**, 130-138 (2010).
27. Clapp, C. E., Allmaras, R. R., Layese, M. F., Linden, D. R. & Dowdy, R. H. Soil organic carbon and 13 C abundance as related to tillage, crop residue, and nitrogen fertilization under continuous corn management in Minnesota. *Soil Till Res* **55**, 127-142 (2000).
28. Coleman, K. *et al.* Simulating trends in soil organic carbon in long-term experiments using RothC-26.3. *Geoderma* **81**, 29-44 (1997).
29. Curtin, D. & Fraser, P. M. Soil organic matter as influenced by straw management practices and inclusion of grass and clover seed crops in cereal rotations. *Soil Res* **41**, 95-106 (2003).
30. Dao, T. H. Tillage and crop residue effects on carbon dioxide evolution and carbon storage in a Paleustoll. *Soil Sci Soc Am J* **62**, 250-256 (1998).
31. Dass, A., Lenka, N. K., Patnaik, U. S. & Sudhishri, S. Integrated nutrient management for production, economics, and soil improvement in winter vegetables. *Int J Veg Sci* **14**, 104-120 (2008).
32. Davis, R. L. *et al.* Nitrogen balance in the Magruder Plots following 109 years in continuous winter wheat. *J Plant Nutr* **26**, 1561-1580 (2003).
33. De Sanctis, G. *et al.* Long-term no tillage increased soil organic carbon content of rain-fed cereal systems in a Mediterranean area. *Eur J Agron* **40**, 18-27 (2012).
34. Dersch, G. & Böhm, K. Effects of agronomic practices on the soil carbon storage potential in

arable farming in Austria. *Nutr Cycl Agroecosys* **60**, 49-55 (2001).

35. Dick, W. A. *et al.* Impacts of agricultural management practices on C sequestration in forest-derived soils of the eastern Corn Belt. *Soil Till Res* **47**, 235-244 (1998).
36. Doane, T. A. & Horwáth, W. R. Annual dynamics of soil organic matter in the context of long-term trends. *Global Biogeochem Cy* **18**, doi:10.1029/2004GB002252 (2004).
37. Dong, W., Hu, C., Chen, S. & Zhang, Y. Tillage and residue management effects on soil carbon and CO2 emission in a wheat–corn double-cropping system. *Nutr Cycl Agroecosys* **83**, 27-37 (2009).
38. Du, Z., Liu, S., Li, K. & Ren, T. Soil organic carbon and physical quality as influenced by long-term application of residue and mineral fertiliser in the North China Plain. *Soil Res* **47**, 585-591 (2009).
39. Du, Z., Liu, S., Xiao, X., Yang, G. & Ren, T. Soil physical quality as influenced by long-term fertilizer management under an intensive cropping system. *Int J Agr Biol* **2**, 19-27 (2009).
40. Duiker, S. W. & Lal, R. Crop residue and tillage effects on carbon sequestration in a Luvisol in central Ohio. *Soil Till Res* **52**, 73-81 (1999).
41. Ebertseder, T., Gutser, R. & Kilian, A. N2O losses from a long-term compost amended soil. *Applying Compost Benefits and Needs*, Federal Ministry of Agriculture, Forestry, Environment and Water Management, Austria, and European Communities, Brussels. 87-90 (2003).
42. Edmeades, D. C. The long-term effects of manures and fertilisers on soil productivity and quality: a review. *Nutr Cycl Agroecosys* **66**, 165-180 (2003).
43. Falloon, P. & Smith, P. Simulating SOC changes in long-term experiments with RothC and CENTURY: model evaluation for a regional scale application. *Soil Use Manage* **18**, 101-111 (2002).
44. Fan, M. *et al.* Crop yields, internal nutrient efficiency, and changes in soil properties in rice–wheat rotations under non-flooded mulching cultivation. *Plant Soil* **277**, 265-276 (2005).
45. Franko, U., Kuka, K., Romanenko, I. A. & Romanenkov, V. Validation of the CANDY model with Russian long-term experiments. *Reg Environ Change* **7**, 79-91 (2007).
46. Franzluebbers, A. J. & Stuedemann, J. A. Soil-profile organic carbon and total nitrogen during 12 years of pasture management in the Southern Piedmont USA. *Agr Ecosyst Environ* **129**, 28-36 (2009).
47. Gami, S. *et al.* Long-term changes in yield and soil fertility in a twenty-year rice-wheat experiment in Nepal. *Biol Fert Soils* **34**, 73-78 (2001).
48. Gami, S. K., Lauren, J. G. & Duxbury, J. M. Soil organic carbon and nitrogen stocks in Nepal long-term soil fertility experiments. *Soil Till Res* **106**, 95-103 (2009).
49. Gangwar, K. S., Singh, K. K., Sharma, S. K. & Tomar, O. Alternative tillage and crop residue management in wheat after rice in sandy loam soils of Indo-Gangetic plains. *Soil Till Res* **88**, 242-252 (2006).
50. García-Orenes, F. *et al.* Effects of agricultural management on surface soil properties and soil–water losses in eastern Spain. *Soil Till Res* **106**, 117-123 (2009).
51. Gentile, R., Vanlauwe, B., Kavoo, A., Chivenge, P. & Six, J. Residue quality and N fertilizer do not influence aggregate stabilization of C and N in two tropical soils with contrasting texture. *Nutr Cycl Agroecosys* **88**, 121-131 (2010).
52. Ghimire, R., Adhikari, K. R., Chen, Z. S., Shah, S. C. & Dahal, K. R. Soil organic carbon sequestration as affected by tillage, crop residue, and nitrogen application in rice–wheat rotation

system. *Paddy Water Environ* **10**, 95-102 (2012).
53. Ghuman, B. S. & Sur, H. S. Tillage and residue management effects on soil properties and yields of rainfed maize and wheat in a subhumid subtropical climate. *Soil Till Res* **58**, 1-10 (2001).
54. Girma, K., Holtz, S. L., Arnall, D. B., Tubana, B. S. & Raun, W. R. The Magruder plots: untangling the puzzle. *Agron J* **100**, 1191-1198 (2008).
55. Goh, K. M., Bruce, G. E., Daly, M. J. & Frampton, C. Sensitive indicators of soil organic matter sustainability in orchard floors of organic, conventional and integrated apple orchards in New Zealand. *Biol Agric Hortic* **17**, 197-205 (2000).
56. Goyal, S., Chander, K., Mundra, M. C. & Kapoor, K. K. Influence of inorganic fertilizers and organic amendments on soil organic matter and soil microbial properties under tropical conditions. *Biol Fert Soils* **29**, 196-200 (1999).
57. Grandy, A. S., Porter, G. A. & Erich, M. S. Organic amendment and rotation crop effects on the recovery of soil organic matter and aggregation in potato cropping systems. *Soil Sci Soc Am J* **66**, 1311-1319 (2002).
58. Gudmundsson, T., Thorvaldsson, G. & Björnsson, H. The effect of different types of fertilisers on the nutrient status in Icelandic Andosols as found in three long-term experiments. In: Bent, T. C., Jens P. & Margit S. (eds.) *Long-term field experiments-a unique research platform*, Askov, pp. 64-67 (2008).
59. Halpern, M. T., Whalen, J. K. & Madramootoo, C. A. Long-term tillage and residue management influences soil carbon and nitrogen dynamics. *Soil Sci Soc Am J* **74**, 1211-1217 (2010).
60. Hati, K. M., Swarup, A., Dwivedi, A. K., Misra, A. & Bandyopadhyay, K. Changes in soil physical properties and organic carbon status at the topsoil horizon of a vertisol of central India after 28 years of continuous cropping, fertilization and manuring. *Agr Ecosyst Environ* **119**, 127-134 (2007).
61. Haynes, R. J. & Naidu, R. Influence of lime, fertilizer and manure applications on soil organic matter content and soil physical conditions: a review. *Nutr Cycl Agroecosys* **51**, 123-137 (1998).
62. Hemmat, A., Aghilinategh, N., Rezainejad, Y. & Sadeghi, M. Long-term impacts of municipal solid waste compost, sewage sludge and farmyard manure application on organic carbon, bulk density and consistency limits of a calcareous soil in central Iran. *Soil Till Res* **108**, 43-50 (2010).
63. Hernanz, J. L., López, R., Navarrete, L. & Sanchez-Giron, V. Long-term effects of tillage systems and rotations on soil structural stability and organic carbon stratification in semiarid central Spain. *Soil Till Res* **66**, 129-141 (2002).
64. Holeplass, H., Singh, B. R. & Lal, R. Carbon sequestration in soil aggregates under different crop rotations and nitrogen fertilization in an inceptisol in southeastern Norway. *Nutr Cycl Agroecosys* **70**, 167-177 (2004).
65. Houot, S., Molina, J. A. E., Clapp, C. E. & Chaussod, R. Simulation by NCSOIL of net mineralization in soils from the Deherain and 36 Parcelles fields at Grignon. *Soil Sci Soc Am J* **53**, 451-455 (1989).
66. Huang, Y. *et al.* Agro-C: A biogeophysical model for simulating the carbon budget of agroecosystems. *Agr Forest Meteorol* **149**, 106-129 (2009).
67. Jiang, G. *et al.* Soil organic carbon sequestration in upland soils of northern China under variable fertilizer management and climate change scenarios. *Global Biogeochem Cy* **28**, 319-333 (2014).
68. Johnston, A. E., Poulton, P. R. & Coleman, K. Soil organic matter: its importance in sustainable agriculture and carbon dioxide fluxes. *Adv Agron* **101**, 1-57 (2009).

69. Jokela, W. E., Grabber, J. H., Karlen, D. L., Balser, T. C. & Palmquist, D. E. Cover crop and liquid manure effects on soil quality indicators in a corn silage system. *Agron J* **101**, 727-737 (2009).
70. Jun, N., Zhou, J., Wang, H., Xiao-Qin, C. & Chang-Wen, D. Effect of long-term rice straw return on soil glomalin, carbon and nitrogen. *Pedosphere* **17**, 295-302 (2007).
71. Kätterer, T., Bolinder, M. A., Andrén, O., Kirchmann, H. & Menichetti, L. Roots contribute more to refractory soil organic matter than above-ground crop residues, as revealed by a long-term field experiment. *Agr Ecosyst Environ* **141**, 184-192 (2011).
72. Kelly, R. H. *et al.* Simulating trends in soil organic carbon in long-term experiments using the century model. *Geoderma* **81**, 75-90 (1997).
73. Kibunja, C. N., Mwaura, F. B. & Mugendi, D. N. Long-term land management effects on soil properties and microbial populations in a maize-bean rotation at Kabete, Kenya. *Afr J Agr Res* **5**, 108-113 (2010).
74. Kimetu, J. M. & Lehmann, J. Stability and stabilisation of biochar and green manure in soil with different organic carbon contents. *Soil Res* **48**, 577-585 (2010).
75. Kirkby, C. A. & Fattore, A. *Effect of Stubble Burning Versus Retention on Soil Health*, Rural Industries Research and Development Corporation. pp. 23-24 (2006).
76. Koga, N. & Tsuji, H. Effects of reduced tillage, crop residue management and manure application practices on crop yields and soil carbon sequestration on an Andisol in northern Japan. *Soil Sci Plant Nutr* **55**, 546-557 (2009).
77. Körschens, M., Weigel, A. & Schulz, E. Turnover of soil organic matter (SOM) and long‐term balances—tools for evaluating sustainable productivity of soils. *Zeitschrift für Pflanzenernährung und Bodenkunde* **161**, 409-424 (1998).
78. Kushwaha, C. P., Tripathi, S. K. & Singh, K. P. Variations in soil microbial biomass and N availability due to residue and tillage management in a dryland rice agroecosystem. *Soil Till Res* **56**, 153-166 (2000).
79. Lal, R. Soil carbon sequestration to mitigate climate change. *Geoderma* **123**, 1-22 (2004).
80. Lee, C. H., Wu, M., Asio, V. B. & Chen, Z.-S. Using a soil quality index to assess the effects of applying swine manure compost on soil quality under a crop rotation system in Taiwan. *Soil Sci* **171**, 210-222 (2006).
81. Lee, D. K., Owens, V. N. & Doolittle, J. J. Switchgrass and soil carbon sequestration response to ammonium nitrate, manure, and harvest frequency on conservation reserve program land. *Agron J* **99**, 462-468 (2007).
82. Leifeld, J., Reiser, R. & Oberholzer, H. R. Consequences of conventional versus organic farming on soil carbon: results from a 27-year field experiment. *Agron J* **101**, 1204-1218 (2009).
83. Li, C., Li, Y. & Tang, L. The effects of long-term fertilization on the accumulation of organic carbon in the deep soil profile of an oasis farmland. *Plant Soil* **369**, 645-656 (2013).
84. Li, L., Zhang, X., Zhang, P., Zheng, J. & Pan, G. Variation of organic carbon and nitrogen in aggregate size fractions of a paddy soil under fertilisation practices from Tai Lake Region, China. *J Sci Food Agr* **87**, 1052-1058 (2007).
85. Li, Z., Liu, M., Wu, X., Han, F. & Zhang, T. Effects of long-term chemical fertilization and organic amendments on dynamics of soil organic C and total N in paddy soil derived from barren land in subtropical China. *Soil Till Res* **106**, 268–274 (2010).
86. Liu, E. *et al.* Long-term effect of chemical fertilizer, straw, and manure on soil chemical and

biological properties in northwest China. *Geoderma* **158**, 173-180 (2010).

87. Lou, Y., Xu, M., Wang, W., Sun, X. & Zhao, K. Return rate of straw residue affects soil organic C sequestration by chemical fertilization. *Soil Till Res* **113**, 70-73 (2011).
88. Machado, P., Sohi, S. P. & Gaunt, J. L. Effect of no-tillage on turnover of organic matter in a Rhodic Ferralsol. *Soil Use Manage* **19**, 250-256 (2003).
89. Majumder, B. *et al.* Organic amendments influence soil organic carbon pools and rice–wheat productivity. *Soil Sci Soc Am J* **72**, 775-785 (2008).
90. Malhi, S. S. & Lemke, R. Tillage, crop residue and N fertilizer effects on crop yield, nutrient uptake, soil quality and nitrous oxide gas emissions in a second 4-yr rotation cycle. *Soil Till Res* **96**, 269-283 (2007).
91. Malhi, S. S. *et al.* Long-term straw management and N fertilizer rate effects on quantity and quality of organic C and N and some chemical properties in two contrasting soils in Western Canada. *Biol Fert Soils* **47**, 785-800 (2011).
92. Marschner, P., Kandeler, E. & Marschner, B. Structure and function of the soil microbial community in a long-term fertilizer experiment. *Soil Biol Biochem* **35**, 453-461 (2003).
93. Mazzoncini, M., Di Bene, C., Coli, A., Risaliti, R. & Bonari, E. Long-term tillage and nitrogen fertilisation effects on maize yield and soil quality under rainfed Mediterranean conditions: a critical perspective. In: Bent, T. C., Jens P. & Margit S. (eds.) *Long-term field experiments-a unique research platform*, Askov, pp. 13-16 (2008).
94. Michels, K., Sivakumar, M. V. K. & Allison, B. E. Wind erosion control using crop residue I. Effects on soil flux and soil properties. *Field Crop Res* **40**, 101-110 (1995).
95. Monaco, S., Hatch, D. J., Sacco, D., Bertora, C. & Grignani, C. Changes in chemical and biochemical soil properties induced by 11-yr repeated additions of different organic materials in maize-based forage systems. *Soil Biol Biochem* **40**, 608-615 (2008).
96. Monreal, C. M. & Janzen, H. H. Soil organic-carbon dynamics after 80 years of cropping a Dark Brown Chernozem. *Can J Soil Sci* **73**, 133-136 (1993).
97. Morari, F., Lugato, E., Berti, A. & Giardini, L. Long‐term effects of recommended management practices on soil carbon changes and sequestration in north‐eastern Italy. *Soil Use Manage* **22**, 71-81 (2006).
98. Mubarak, A. R. & Rosenani, A. B. Soil organic matter fractions in humid tropics as influenced by application of crop residues. *Commun Soil Sci Plan* **34**, 933-943 (2003).
99. Murata, T. & Goh, K. M. Effects of cropping systems on soil organic matter in a pair of conventional and biodynamic mixed cropping farms in Canterbury, New Zealand. *Biol Fert Soils* **25**, 372-381 (1997).
100. Nafziger, E. D. & Dunker, R. E. Soil organic carbon trends over 100 years in the Morrow plots. *Agron J* **103**, 261-267 (2011).
101. Naser, H. M., Nagata, O., Tamura, S. & Hatano, R. Methane emissions from five paddy fields with different amounts of rice straw application in central Hokkaido, Japan. *Soil Sci Plant Nutr* **53**, 95-101 (2007).
102. Nayak, P., Patel, D., Ramakrishnan, B., Mishra, A. & Samantaray, R. Long-term application effects of chemical fertilizer and compost on soil carbon under intensive rice–rice cultivation. *Nutr Cycl Agroecosys* **83**, 259-269 (2009).
103. Nevens, F. Combining compost and slurry in intensive Flemish silage maize production: fate of nitrogen. *Applying Compost Benefits and Needs*, Federal Ministry of Agriculture, Forestry,

Environment and Water Management, Austria, and European Communities, Brussels. 59-75 (2003).

104. Nguyen, M. L., Haynes, R. J. & Goh, K. M. Nutrient budgets and status in three pairs of conventional and alternative mixed cropping farms in Canterbury, New Zealand. *Agr Ecosyst Environ* **52**, 149-162 (1995).

105. Niu, L., Hao, J., Zhang, B. & Niu, X.-S. Influences of long-term fertilizer and tillage management on soil fertility of the North China Plain. *Pedosphere* **21**, 813-820 (2011).

106. Ouédraogo, E., Mando, A. & Stroosnijder, L. Effects of tillage, organic resources and nitrogen fertiliser on soil carbon dynamics and crop nitrogen uptake in semi-arid West Africa. *Soil Till Res* **91**, 57-67 (2006).

107. Page, K. L. *et al.* Organic carbon stocks in cropping soils of Queensland, Australia, as affected by tillage management, climate, and soil characteristics. *Soil Res* **51**, 596-607 (2014).

108. Parham, J., Deng, S., Raun, W. & Johnson, G. Long-term cattle manure application in soil. *Biol Fert Soils* **35**, 328-337 (2002).

109. Plaza, C., Gollany, H. T., Baldoni, G., Polo, A. & Ciavatta, C. Predicting long-term organic carbon dynamics in organically amended soils using the CQESTR model. *J Soil Sediment* **12**, 486-493 (2012).

110. Prasad, R., Gangaiah, B. & Aipe, K. C. Effect of crop residue management in a rice–wheat cropping system on growth and yield of crops and on soil fertility. *Exp Agr* **35**, 427-435 (1999).

111. Rasmussen, P. E. & Parton, W. J. Long-term effects of residue management in wheat-fallow: I. Inputs, yield, and soil organic matter. *Soil Sci Soc Am J* **58**, 523-530 (1994).

112. Reganold, J. P., Palmer, A. S., Lockhart, J. C. & Macgregor, A. N. Soil quality and financial performance of biodynamic and conventional farms in New Zealand. *Science* **260**, 344-344 (1993).

113. Roldán, A. *et al.* No-tillage, crop residue additions, and legume cover cropping effects on soil quality characteristics under maize in Patzcuaro watershed (Mexico). *Soil Till Res* **72**, 65-73 (2003).

114. Ros, M., Pascual, J. A., Garcia, C., Hernandez, M. & Insam, H. Hydrolase activities, microbial biomass and bacterial community in a soil after long-term amendment with different composts. *Soil Biol Biochem* **38**, 3443-3452 (2006).

115. Saha, R., Mishra, V. K., Majumdar, B., Laxminarayana, K. & Ghosh, P. Effect of integrated nutrient management on soil physical properties and crop productivity under a maize (Zea mays)–mustard (Brassica campestris) cropping sequence in acidic soils of Northeast India. *Commun Soil Sci Plan* **41**, 2187-2200 (2010).

116. Saha, R., Nath, V. & Kumar, D. Effects of farmyard manure on soil organic carbon stock, the pattern of fertility build-up, and plant growth in'Mallika'mango (Mangifera indica L.). *J Hortic Sci Biotech* **85**, 539 (2010).

117. Saha, S., Mina, B. L., Gopinath, K. A., Kundu, S. & Gupta, H. Organic amendments affect biochemical properties of a subtemperate soil of the Indian Himalayas. *Nutr Cycl Agroecosys* **80**, 233-242 (2008).

118. Sainju, U. M., Senwo, Z. N., Nyakatawa, E. Z., Tazisong, I. A. & Reddy, K. C. Tillage, cropping systems, and nitrogen fertilizer source effects on soil carbon sequestration and fractions. *J Environ Qual* **37**, 880-888 (2008).

119. Sainju, U. M., Singh, B. P. & Whitehead, W. F. Long-term effects of tillage, cover crops, and

nitrogen fertilization on organic carbon and nitrogen concentrations in sandy loam soils in Georgia, USA. *Soil Till Res* **63**, 167-179 (2002).

120. Salinas-Garcia, J. R., Baez-Gonzalez, A. D., Tiscareno-Lopez, M. & Rosales-Robles, E. Residue removal and tillage interaction effects on soil properties under rain-fed corn production in Central Mexico. *Soil Till Res* **59**, 67-79 (2001).
121. Sarkar, S., Singh, S. R. & Singh, R. P. The effect of organic and inorganic fertilizers on soil physical condition and the productivity of a rice–lentil cropping sequence in India. *J Agr Sci* **140**, 419-425 (2003).
122. Sharma, K. L. *et al.* Effect of soil and nutrient-management treatments on soil quality indices under cotton-based production system in rainfed semi-arid tropical vertisol. *Commun Soil Sci Plan* **42**, 1298-1315 (2011).
123. Sharma, K. L. *et al.* Long-term soil management effects on crop yields and soil quality in a dryland Alfisol. *Soil Till Res* **83**, 246-259 (2005).
124. Sharma, S. N. *et al.* Crop Diversification and Residue Incorporation for Making Rice–Wheat Cropping Systems Sustainable. *J Sustain Agr* **34**, 342-364 (2010).
125. Shen, M. *et al.* Long-term effects of fertilizer managements on crop yields and organic carbon storage of a typical rice–wheat agroecosystem of China. *Biol Fert Soils* **44**, 187-200 (2007).
126. Shevtsova, L. *et al.* Effect of natural and agricultural factors on long-term soil organic matter dynamics in arable soddy-podzolic soils—modeling and observation. *Geoderma* **116**, 165-189 (2003).
127. Shirato, Y. *et al.* Testing the Rothamsted Carbon Model against data from long‐term experiments on upland soils in Thailand. *Eur J Soil Sci* **56**, 179-188 (2005).
128. Shirato, Y. & Yokozawa, M. Applying the Rothamsted Carbon Model for long-term experiments on Japanese paddy soils and modifying it by simple tuning of the decomposition rate. *Soil Sci Plant Nutr* **51**, 405-415 (2005).
129. Singh, K. N., Prasad, B. & Sinha, S. K. Effect of integrated nutrient management on a Typic Haplaquant on yield and nutrient availability in a rice-wheat cropping system. *Crop Pasture Sci* **52**, 855-858 (2001).
130. Singh, K. P., Suman, A., Singh, P. N. & Srivastava, T. Improving quality of sugarcane-growing soils by organic amendments under subtropical climatic conditions of India. *Biol Fert Soils* **44**, 367-376 (2007).
131. Soon, Y. K. Crop residue and fertilizer management effects on some biological and chemical properties of a Dark Grey Solod. *Can J Soil Sci* **78**, 707-713 (1998).
132. Srinivasarao, C. H. *et al.* Long‐term manuring and fertilizer effects on depletion of soil organic carbon stocks under pearl millet‐cluster bean‐castor rotation in western india. *Land Degrad Dev* **25**, 173-183 (2014).
133. Su, Y., Wang, F., Suo, D., Zhang, Z.-H. & Du, M.-W. Long-term effect of fertilizer and manure application on soil-carbon sequestration and soil fertility under the wheat–wheat–maize cropping system in northwest China. *Nutr Cycl Agroecosys* **75**, 285-295 (2006).
134. Surekha, K., Kumari, A. P., Reddy, M. N., Satyanarayana, K. & Cruz, P. S. Crop residue management to sustain soil fertility and irrigated rice yields. *Nutr Cycl Agroecosys* **67**, 145-154 (2003).
135. Thuithaisong, C. *et al.* Soil-quality indicators for predicting sustainable organic rice production. *Commun Soil Sci Plan* **42**, 548-568 (2011).

136. Tian, K. *et al.* Effects of long-term fertilization and residue management on soil organic carbon changes in paddy soils of China: A meta-analysis. *Agr Ecosyst Environ* **204**, 40-50 (2015).
137. Tirol‐Padre, A., Tsuchiya, K., Inubushi, K. & Ladha, J. K. Enhancing Soil Quality through Residue Management in a Rice‐Wheat System in Fukuoka, Japan. *Soil Sci Plant Nutr* **51**, 849-860 (2005).
138. van Groenigen, K. J. *et al.* Soil C storage as affected by tillage and straw management: An assessment using field measurements and model predictions. *Agr Ecosyst Environ* **140**, 218-225 (2011).
139. Veenstra, J. J., Horwath, W. R. & Mitchell, J. P. Tillage and cover cropping effects on aggregate-protected carbon in cotton and tomato. *Soil Sci Soc Am J* **71**, 362-371 (2007).
140. Verma, T. S. & Bhagat, R. M. Impact of rice straw management practices on yield, nitrogen uptake and soil properties in a wheat-rice rotation in northern India. *Fert Res* **33**, 97-106 (1992).
141. Viaud, V., Angers, D. A., Parnaudeau, V., Morvan, T. & Aubry, S. M. Response of organic matter to reduced tillage and animal manure in a temperate loamy soil. *Soil Use Manage* **27**, 84-93 (2011).
142. Wang, G., Huang, Y., Wang, E., Yu, Y. & Zhang, W. Modeling Soil Organic Carbon Change across Australian Wheat Growing Areas, 1960-2010. *Plos One* **8**, e63324 (2013).
143. Wang, J., Chen, Z., Chen, L., Zhu, A. & Wu, Z. Surface soil phosphorus and phosphatase activities affected by tillage and crop residue input amounts. *Plant Soil Environ* **57**, 251-257 (2011).
144. Yadav, R. *et al.* Yield trends, and changes in soil organic-C and available NPK in a long-term rice–wheat system under integrated use of manures and fertilisers. *Field Crop Res* **68**, 219-246 (2000).
145. Yang, C., Yang, L. & Ouyang, Z. Organic carbon and its fractions in paddy soil as affected by different nutrient and water regimes. *Geoderma* **124**, 133-142 (2005).
146. Yang, X., Yang, Y., Sun, B. & Zhang, S. Long-term fertilization effects on yield trends and soil properties under a winter wheat-summer maize cropping system. *Afr J Agr Res* **6**, 3392-3401 (2011).
147. Yu, H., Ding, W., Luo, J., Geng, R. & Cai, Z. Long-term application of organic manure and mineral fertilizers on aggregation and aggregate-associated carbon in a sandy loam soil. *Soil Till Res* **124**, 170-177 (2012).
148. Yu, Y., Huang, Y. & Zhang, W. Modeling soil organic carbon change in croplands of China, 1980–2009. *Global Planet Change* **82**, 115-128 (2012).
149. Zeleke, T. B., Grevers, M. C. J., Si, B. C., Mermut, A. & Beyene, S. Effect of residue incorporation on physical properties of the surface soil in the South Central Rift Valley of Ethiopia. *Soil Till Res* **77**, 35-46 (2004).
150. Zhang, G., Chan, K., Li, G. & Huang, G. Effect of straw and plastic film management under contrasting tillage practices on the physical properties of an erodible loess soil. *Soil Till Res* **98**, 113-119 (2008).
151. Zhao, Y. *et al.* The effects of two organic manures on soil properties and crop yields on a temperate calcareous soil under a wheat–maize cropping system. *Eur J Agron* **31**, 36-42 (2009).
152. Zhu, H. *et al.* Improving fertility and productivity of a highly-weathered upland soil in subtropical China by incorporating rice straw. *Plant Soil* **331**, 427-437 (2010).
153. Zwart, K. Fate of C and N pools–experience from short and long term compost experiments.

*Applying Compost Benefits and Needs*, Federal Ministry of Agriculture, Forestry, Environment and Water Management, Austria, and European Communities, Brussels. 77-85 (2003).

## Data from China Knowledge Resource Integrated Database (1990-2014):

1. Bi, D., Zhang, R., Wang, J., Wang, X. J. & Cai, L. Q. Effect of different tillage patterns on organic carbon pool and microbial quotient in two sequence rotation system with spring wheat and field pea. *Agr Res Arid Areas* **27**, 11-16,22 (2009).
2. Cai, T. *et al.* Soil labile organic carbon and carbon pool management index as affected by different years no-tilling with straw mulching. *Chin J Ecol* **30**, 1962-1968 (2011).
3. Cai, X. *et al.* Effect of straw returning on the environment of degenerated soil in centr al Tibet. *Plant Nutr Fert Sci* **9**, 411-415 (2003).
4. Chen, H., Han, X., Yang, J., Wu, Z. & Zuo, R. The Correlation of Soil Organic Carbon Content of Long-term Fertilization and Climate Factors. *Chin J Soil Sci* **41**, 622-626 (2010).
5. Chen, J. *et al.* Application of principal component analysis in evaluation of soil quality under different long-term fertilization. *Soils* **42**, 415-420 (2010).
6. Chen, S., Li, J., Lu, P., Wang, Y. & Yu, Q. Soil respiration characteristics in winter wheat field in North China Plain. *Chin J Appl Ecol* **15**, 1552-1560 (2004).
7. Chen, S., Zhu, Z., Liu, D., Shu, L. & Wang, C. Q. Influence of straw mulching with no-till on soil nutrients and carbon pool management index. *Plant Nutr Fert Sci* **14**, 806-809 (2008).
8. Chen, X., Yue, X., Ge, X. & Wang, X. d. Effect of long-term residue return on soil organic carbon storage. *J Nat Res* **27**, 25-32 (2012).
9. Ding, Y., Li, J., Yan, H., Wen-Xiang, H. E. & Tian, X. H. Effects of straw returning on soil enzyme activity. *J Northwest A F Univ* **42**, 137-144 (2014).
10. Dong, L., Li, Y., Pang, H. & Sun, Q. Comparison of the effect of long-term fertilizer application on soil nutrients and wheat yield under different soil types. *J. China Agric. U* **15**, 22-28 (2010).
11. Dong, S. *et al.* Effect of different Nitrogen Application Methods on Soil Organic Matter and Nitrogen Content. *Sotbean Sci* **29**, 836-835 (2010).
12. Dong, Y., Fan, H., Wang, J. & Wang, J. F. Preliminary Report of returning corn straw into soil on soil fertility. *Guangdong Agr Sci* **37**, 77-79 (2010).
13. Duan, H., Niu, Y. & Bian, X. Effects of tillage mode and straw return on soil organic carbon and rice yield in direct seeding rice field. *Bull Soil Water Conserv* **32**, 23-27 (2012).
14. Fan, B. & Liu, Q. Effect of conservation tillage and straw application on the soil microorganism and P-dissolving characteristics. *Chin J Eco-Agr* **13**, 130-132 (2004).
15. Gao, X. *et al.* Effects of Straw Application on Soil Organic Carbon and Active Organic Carbon in Wheat-corn Rotation System. *J Henan Agr Sci* **41**, 63-67 (2012).
16. Gong, L., Sun, W. & Wang, C. Effects of Application Maize Straw on Soil Physical Characteristics and Yield. *J Maiz Sci* **16**, 122-122 (2008).
17. Guo, S., Wu, J. & Dang, T. Effects of Crop Rotation and Fertilization on Aboveground Biomass and Soil Organic C in Semi-arid Region. *Scien Agri Sinica* **41**, 744-751 (2008).
18. Zeng, J., Guo, T., Bao, X. & Sun, W. Effections of soil organic carbon and soil inorganic carbon under long-term fertilization. *Soil Fert Sci Chin*, 11-14 (2008).
19. Han, X., Zhu, L., Yang, M., Qi, Y. U. & Xin-min, B. Effects of Different Amount of Wheat Straw Returning on Rice Growth,Soil Microbial Biomass and Enzyme Activity. *J Agro-Environ Sci* **31**,

2192-2199 (2012).

20. Hao, X. *et al.* Effects of Continuous Straw Returning on Organic Carbon Content in Aggregates and Fertility of Black Soil. *Chin Agr Sci Bull* **29**, 263-269 (2013).
21. He, L. *et al.* PCR-DGGE analysis of soil bacterium community diversity in farmland influenced by biochar. *Acta Ecol. Sin.* **34**, 4288-4294 (2014).
22. Hou, X. *et al.* Effects of Different Fertilization on Soil Organic Carbon in Costal Saline Soil Region. *Soils* **46**, 780-786 (2014).
23. Hu, C., Qian, Y., Li, S., Chen, Y. & Liu, G. Vertical distribution and storage of soil organic carbon under long-term fertili zation (in Chinese with English abstract). *Chin J Eco-Agr* **18**, 689-692 (2010).
24. Jia, W. *et al.* Effects of Long-term Returning Corn Stalks to the Field Combined with Applying Fertilizer in Autumn on Microbial Biomass C,N and Enzyme Activity in Cinnamon Soil. *Acta Agriculturae Boreali-Sinica* **23**, 138-142 (2008).
25. Jiao, L., Li Zhihong, Yin Chengcheng & al., e. Effects of different stalk returned depth on soil humus and soil anzyme in black soil. *Soil Fert Sci Chin*, 17-21 (2015).
26. Jiao, X., Wei, D. & Sui, Y. Effects of Long-term Fertilization on the Soil enzyme Activities and Soil Nutrients of the Black and Dark Brown Soils. *Chin J Soil Sci* **42**, 698-703 (2011).
27. Lei, H. *et al.* Modeling and Applications of Soil Organic Matter in Intensive Cropping in China's Huang-Huai-Hai Plain. *Scien Agri Sinica* **38**, 956-964 (2005).
28. Li, C. *et al.* Effects of Rape Residue Mulch on Greenhouse Gas Emissions and Carbon Sequestration from No-tillage Rice Fields. *J Agro-Environ Sci* **30**, 2362-2367 (2011).
29. Li, L. Effect of the Straw Returned to Field on the Ling-term Improvement of Shajiang Black Soil. *J of Anhui Agr Sci* **29**, 765-766 (2001).
30. Li, L. Effects of straw returning and applying decomposition agent on rice yield and soil fertility. *Agr Technol Serv* **32**, 98 (2015).
31. Li, Q. Effects of long-term fertilization on carbon, nitrogen fractions and microbial diversity in red paddy soil. Master thesis, Fujian Agriculture and Forestry University, Fuzhou. 7-10 (2011).
32. Lin, X., Zhang, Y. & He, N. Effects of straw application methods on crop yield and soil fertility in a triple cropping paddy soil. *J Zhejiang Agr Univ* **23**, 273-276 (1997).
33. Liu, K., Luo, C., Chen, M. & Yi, G. The reddish paddy soil soil fertility evolution in the long-term fertilization experiment. In: Xu M., Liang G., Zhang F. (Eds.). *Evolution of Soil Fertility in China*, China Agricultural Science and Technology Press, Beijing, pp. 137–141 (2006).
34. Liu, P. *et al.* Effects of conservation tillage on soil physicochemical properties in the spring maize area of the Loess Plateau. *Agr Res Arid Areas* **27**, 171-175 (2009).
35. Liu, S. *et al.* Effects of tillage and straw returning on soil fertility and grain yield in a wheat-rice double cropping system. *Transact Chin Soc Agr Eng* **22**, 48-51 (2006).
36. Liu, S., Tong, C., Wu, J. & Jiang, p. Effect of ratio of organic manure/chemical fertilizer in fertilization on rice yield under the same N condition. *Acta Pedol Si* **44**, 106-112 (2007).
37. Liu, S., Zhou, X., Li, L. & Yang, G. l. Effects of corn straw return on rice yield and soil fertility. *Crop Res* **17**, 131-132 (2003).
38. Liu, W., Li, H., Lu, J. & Xiaokun, L. Effects of conservation tillages on soil nutrients,structure and crop yield. *Chin J Soil Sci* **46**, 420-427 (2015).
39. Liu, H., Wang, X., Li, G. & Wang, J. Grey desert soil quality evolution in a long-term fertilization experiment. In: Xu M., Liang G., Zhang F. (Eds.). *Evolution of Soil Fertility in China*, China

Agricultural Science and Technology Press, Beijing, pp. 209-211 (2006).
40. Liu, Y. *et al.* Effects of long-term straw mulch and fertilization on crop yields and soil physical and chemical properties under rice-rapeseed rotation. *J Plant Nutr Fert* **20**, 1450-1459 (2014).
41. Liu, Z. *et al.* Effect of Straw Return on Maize Yield Components and Soil Fertility. *Heilongjiang Agr Sci*, 42-45 (2014).
42. Saha, R., Nath, V. & Kumar, D. Effects of farmyard manure on soil organic carbon stock, the pattern of fertility build-up, and plant growth in'Mallika'mango (Mangifera indica L.). *J Hortic Sci Biotech* **85**, 539 (2010).
43. Ma, L. *et al.* Humus composition and stable carbon isotope natural abundance in paddy soil under long-term fertilization. *Chin J Appl Ecol* **19**, 1951-1958 (2008).
44. Ma, L. *et al.* Effects of Fertilization and Straw Returning on Distribution and Mineralization of Organic Carbon in Paddy Soils in Subtropical China. *Soils* **43**, 883-889 (2011).
45. Mu, P., Zhang, E., Wang, H. & Fang, Y. F. Effects of Continuous Returning Straw to Maize Tilth Soil on Chemical Character and Microbial Biomass. *J Soil Water Conserv* **25**, 81-85 (2011).
46. Mulati, A., Tong, Y., Yang, X. & Hai-Yang, M. A. Effect of Different Fertilization on Soil Organic Carbon and its Fraction in Farmland. *Chin J Soil Sci* **43**, 1461-1466 (2012).
47. Pan, S., Li, J. & Wang, H. Study on changes of soil organic matter in paddy soil under long-term experiments. *Soil Fert Sci Chin*, 8-14 (2011).
48. Qian, H., Han, C., Qian, C. & Yan, G. z. Study on the technology of direct returning of rice and wheat straw. *Soil Fert Sci Chin*, 26-28 (1998).
49. Qin, R., Chen, F. & Gao, J. Long-Term Application of chemical fertilizers and rice straw on soil aluminum toxicity. *Commun Soil Sci Plant Anal* **42**, 66-74 (2010).
50. E, S. *et al.* Long‐term fertilization and manuring effects on physically separated soil organic‐matter pools under continuous wheat cropping at a rainfed semiarid site in China. *J Plant Nutr Soil Sci* **175**, 689-697 (2012).
51. Su, W. The effects and mechanism of straw retaining on winter oilseed rape growth and soil fertility. Ph.D. thesis. Huazhong Agricultural University, Wuhan. 50-63 (2014).
52. Sun, C., Sun, Z., Wei, Z. & Tang, F. Effects of different previous crops with their straw returned to the soil on the soil property and yielld of late-rice. *J Guangxi Agr Col*, 20-27 (1987).
53. Sun, J., Liu, M., Li, L. & Liu, J. H. Influence of non-tillage and stubble on soil microbial biomass and enzyme activities in rain-fed field of Inner Mongolia. *Acta Ecol. Sin.* **29**, 5508-5515 (2009).
54. Sun, T., Li, S. & Shao, M. Effects of long-term fertilization on distribution of organic matters and nitrogen in cinnamon soil aggregates. *Scien Agri Sinica* **38**, 1841-1848 (2005).
55. Sun, W., Yang, B., Qian, H., Wang, S. & Huang, G. Effects of Returning Rice Straw to Fields with Different Ratio of Fertilizer on Rice Yield and Soil Fertility. *J Agr* **2**, 16-21 (2012).
56. Sun, X., Qin, Y., Feng, W. & Tu, S.-h. The calcareous purple soil fertility evolution in the long-term fertilization experiment. In: Xu M., Liang G., Zhang F. (Eds.). *Evolution of Soil Fertility in China*, China Agricultural Science and Technology Press, Beijing, pp. 137–141 (2006).
57. Tian, S. *et al.* Effects of different tillage methods and straw-returning on soil organic carbon content in a winter wheat field. *Chin J Appl Ecol* **21**, 373-378 (2010).
58. Tian, Y. Study of effects of straw returning and reducing fertilizer application on crop yield and soil nutrients. Master thesis, Anhui Agricultural University, Hefei. 14-16, 43 (2012).
59. Wang, D., Zhou, L., Huang, S., Cheng-Fang, L. I. & Cao, C. G. Short-term Effects of Tillage

Practices and Wheat-straw Returned to the Field on Topsoil Labile Organic Carbon Fractions and Yields in Central China. *J Agro-Environ Sci* **32**, 735-740 (2013).

60. Wang, F., Zhang, J., Gao, P. & Tong, Y. A. Short term effect of applying organic materials in improving soil fertility of Weibei rainfed highland. *Chin J Appl Ecol* **21**, 930-936 (2010).
61. Wang, G., Li, L., Hao, M. & Hong, J. Effect of long-term fertilization, stubble mulch and irrigation under different fertilization on soil enzyme and soil nutrients. *J Nucl Agr Sci* **26**, 129-134 (2012).
62. Wang, J., Zhang, H., Xu, Y., Ning, X.-j. & Jie, Y.-w. M. H.-b. C. Effect of different ratios of chicken manure N on organic matter accumulation and acidification of paddy soils. *J Plant Nutr Fert* **20**, 1178-1185 (2014).
63. Wang, S. Relationship between Fertilization Methods and Soil Fertility of Purplish Paddy Soil. *Rur Eco-Environ* **16**, 23-26 (2000).
64. Wang, S., Chen, Y. & Li, S. Balance of Soil organic matter in a long-term triple cropping system in paddy fields. *Acta Pedol Si* **39**, 9-15 (2002).
65. Wang, X., Cai, D., Zhang, J. & Gao, X. Effects of Corn Stover Incorporated in Dry Farmland on Soil Fertility. *Scien Agri Sinica* **33**, 54-61 (2000).
66. Wang, Y. Effect of straw returning and plastic film mulching coupling on maize yield and the balance of organic matter in the Loess Plateau. Ph.D. thesis, Lanzhou University, Lanzhou. 70-72 (2014).
67. Wei, F. & Hao, M. Effects of long-term fertilization on soil water and nutrient with Triticum aestivum L.succession in dryland of Loess Plateau. *Sci Soil Water Conserv* **9**, 104-109 (2011).
68. Wu, J. *et al.* Decomposition characteristics of wheat straw and effects on soil biological properties and nutrient status under different rice cultivation. *Acta Ecol. Sin.* **33**, 565-575 (2013).
69. Wu, R., Wang, Y., Li, F. & Li, X. Effects of coupling film-mulched furrow-ridge cropping with maize straw soil- incorporation on maize yields and soil organic carbon pool at a semiarid loess site of China. *Acta Ecol. Sin.* **32**, 2855-2862 (2012).
70. Wu, Z., Zhang, H., Guangshan, X. & Zhang, Y. Effect of returning corn straw into soil on soil fertility. *Chin J Appl Ecol* **13**, 539-542 (2002).
71. Xu, N., Zhou, L. & Miao, S. Effect of fertilization on organic matter and its composition in mollisols. *Soil Fert Sci Chin*, 14-33 (2012).
72. Xu, Y., Ma, Q., Zhou, H. & al., e. Effects of straw returning and deep loosening on soil physical and chemical properties and maize yields [J]. *Chin J Soil Sci* **46**, 428-432 (2015).
73. Yan, C., Liu, E., He, W., Liu, S. & Liu, Q. Effect of different tillage on soil organic carbon and its fractions in the loess plateau of China. *Soil Fert Sci Chin* **28**, 58-63 (2010).
74. Yan, L. *et al.* Effects of Maize Stems Returning Back to the Field on the Yield of Plants and Soil Fertility. *Chin J Soil Sci* **35**, 143-148 (2004).
75. Yan, X. Application of Straw Returning of Early Rice in Guangxi and Its Effect on Soil Fertility and Yield of Late Rice. Master thesis, Guangxi University, Nanning. 17-20,38 (2013).
76. Yuan, Y., Li, H. & Huang, Q. Effects of Long-Term Fertilization on Dynamics of Soil Organic Carbon in Red Paddy Soil. *Soils* **40**, 237-242 (2008).
77. Zhang, A. & Zhang, M. Organic and inorganic fertilizers affecting the content and composition of soil organic matter-A report of long-term experiment. *Jiangsu Agr Res* **22**, 30-33 (2001).
78. Zhang, D. *et al.* Dynamic Change of Soil Organic Matter Quality as Affected by Different Long-term Fertilization Treatments. *Chin J Soil Sci*, 251-255 (2007).

79. Zhang, J., Wen, X., Liao, Y. & Liu, Y. Effects of different amount of maize straw returning on soil fertility and yield of winter wheat. *Plant Nutr Fert Sci* **16**, 612-619 (2010).
80. Zhang, P. *et al.* Effects of Straw Returning on Soil Organic Carbon and Carbon Mineralization in Semi-arid Areas of Southern Ningxia,China. *J Agro-Environ Sci* **30**, 2518-2525 (2011).
81. Zhang, Y. *et al.* Effect of Mechanized Straw Application on Crop Yields and Soil Labile Carbon Content. *Jiangsu J of Agr Sci* **24**, 833-838 (2008).
82. Zheng, L., Xie, H. & Zhang, W. Effects of different ways of returning straw to the soils on soluble organic carbon. *Ecol Environ* **15**, 80-83 (2006).
83. Zhong, H., Zhang, Y., Lin, C. & Jiang, X. Means of all wheat and rice straw application without chopping and cultivating and its effect on crop yield and soil fertility. *Soils Fert*, 34-37 (2003).
84. Zhou, B., Qiao, M. & Wang, Z. Effects of a long-term located fertilization on soil quality of grey desert soil. *Chin J Eco-Agr* **15**, 33-36 (2007).
85. Zhu, P., Peng, C., Gao, H., Ren, J. & Liu, S. Black soil fertility evolution in the longterm fertilization experiment. In: Xu M., Liang G., Zhang F. (Eds.). *Evolution of Soil Fertility in China*, China Agricultural Science and Technology Press, Beijing, pp. 301–303 (2006).
